# Consumption of sugar sweetened beverages, artificially sweetened beverages and fruit juices and risk of type 2 diabetes, hypertension, cardiovascular disease, and mortality: A meta-analysis

**DOI:** 10.3389/fnut.2023.1019534

**Published:** 2023-03-15

**Authors:** Baoyu Li, Ni Yan, Hong Jiang, Meng Cui, Min Wu, Lina Wang, Baibing Mi, Zhaofang Li, Jia Shi, Yahui Fan, Mougni Mohamed Azalati, Chao Li, Fangyao Chen, Mao Ma, Duolao Wang, Le Ma

**Affiliations:** ^1^School of Public Health, Xi’an Jiaotong University Health Science Center, Xi’an, China; ^2^The First Affiliated Hospital, Xi’an Jiaotong University Health Science Center, Xi’an, China; ^3^Department of Clinical Sciences, Liverpool School of Tropical Medicine, Liverpool, United Kingdom; ^4^Key Laboratory of Environment and Genes Related to Diseases (Xi’an Jiaotong University), Ministry of Education of China, Xi’an, China

**Keywords:** sugar sweetened beverages, artificially sweetened beverages, fruit juices, hypertension, type 2 diabetes, cardiovascular disease, mortality

## Abstract

**Introduction:**

Sugar-sweetened beverage (SSB) intake is associated with an increased risk of cardiometabolic diseases. However, evidence regarding associations of artificially sweetened beverages (ASBs) and fruit juices with cardiometabolic diseases is mixed. In this study, we aimed to investigate the association between the SSB, ASB and fruit juice consumption with the incidence of cardiometabolic conditions and mortality.

**Methods:**

Relevant prospective studies were identified by searching PubMed, Web of Science, Embase, and Cochrane Library until December 2022 without language restrictions. The pooled relative risk (RR) and 95% confidence intervals (CIs) were estimated for the association of SSBs, ASBs, and fruit juices with the risk of type 2 diabetes (T2D), cardiovascular disease (CVD), and mortality by using random-effect models.

**Results:**

A total of 72 articles were included in this meta-analysis study. Significantly positive associations were observed between the consumption of individual beverages and T2D risk (RR: 1.27; 95% CI: 1.17, 1.38 for SSBs; RR: 1.32; 95% CI: 1.11, 1.56 for ASBs; and RR:0.98; 95% CI: 0.93, 1.03 for fruit juices). Moreover, our findings showed that intakes of SSBs and ASBs were significantly associated with risk of hypertension, stroke, and all-cause mortality (RR ranging from 1.08 to 1.54; all *p* < 0.05). A dose-response meta-analysis showed monotonic associations between SSB intake and hypertension, T2D, coronary heart disease (CHD), stroke and mortality, and the linear association was only significant between ASB consumption and hypertension risk. Higher SSB and ASB consumptions were associated with a greater risk of developing cardiometabolic diseases and mortality. Fruit juice intake was associated with a higher risk of T2D.

**Conclusion:**

Therefore, our findings suggest that neither ASBs nor fruit juices could be considered as healthier beverages alternative to SSBs for achieving improved health.

**Systematic Review Registration:** [PROSPERO], identifier [No. CRD42022307003].

## Introduction

The health effects of beverages especially sugar sweetened beverages (SSBs), have received widespread attention from the scientific community and general public over the past decades. Despite public health measures to limit and reduce the consumption of added sugars by introducing sugar tax on SSBs in several countries, SSBs still remain a major source of added sugars (1–6). The most recent 2020–2025 dietary guidelines for Americans carried further forward a recommendation from the 2015 that intake of SSB should be limited (7, 8). SSBs have high carbohydrate content, but poor energy compensation, and induce low satiety effects (9). Evidence from experimental studies have demonstrated that high intake of SSBs has detrimental effect on health benefits, including accelerating chronic inflammation, disordering lipid metabolism, and promoting oxidative stress (10, 11). Therefore, it is important to identify alternatives to SSBs with less adverse health effects.

According to Dietary Guidelines for Americans 2020–2025, everyone can benefit from shifting food and beverage choices to better support healthy dietary patterns (7). Some types of artificially sweetened beverages (ASBs), such as low-and no-calorie sweetened beverages, are considered as the potential replacement of SSBs to displace calories from such beverage by several obesity and T2D organizations (12–18). As another potential alternative to SSBs, fruit juices contain various nutritious vitamins, minerals, and other bioactive phytochemicals such as polyphenols and carotenoid, however, they tend to be rich in naturally occurring sugars. Furthermore, compared with whole fruits, fruit juices contain fewer dietary fibers (19). Although several epidemiological studies have reported that consumption of SSBs was positively associated with cardiometabolic diseases, the associations of ASBs and fruit juices with cardiometabolic disease risk have been less consistent. Some studies found that intake of ASBs was associated with a lower diabetes risk, though some randomized controlled trials have suggested that artificial sweeteners might actually increase glucose intolerance (9, 20, 21). Compared with the literature on T2D, results on the association of ASBs and fruit juice consumption with cardiometabolic diseases (such as hypertension, CHD) or mortality were mixed. In addition, the dose–response relationship between beverage intakes and risk of these diseases or mortality has not been well established. Some studies have demonstrated a monotonic increasing risk with increasing intake of some beverage subtypes, while others have indicated a J-shaped relationship.

Thus, to better illustrate the conflicting results of the previous studies, a dose–response meta-analysis of prospective cohort studies was conducted to comprehensively evaluate the association between the consumption of SSBs, ASBs as well as fruit juices and the risk of cardiometabolic diseases and mortality.

## Materials and methods

The present study followed the Preferred Reporting Items for Systematic Reviews and Meta-analyses (PRISMA) guidelines (22) and recorded in the PROSPERO (CRD42022307003; Supplementary Table 1).

### Literature search strategy and selection criteria

A systematic search of PubMed, EMBASE, Web of Science, and Cochrane Library was performed to identify relevant articles up to December 2022 without language restriction by using MeSH terms and free-text terms (Supplementary Table 2). Reference lists of included studies and relevant articles were also manually searched for potential eligible studies.

Studies meeting the following criteria were eligible for inclusion: (1) the study design was prospective cohort studies in adults aged ≥ 18 years with follow-up of at least 2 years (2) the exposures of interest were consumption of SSBs (beverages containing added caloric sweeteners such as sucrose or high-fructose corn syrup, including soft drinks, fruit-flavored drinks, cola-type beverages, sugar-added fruit juices, sweetened coffees and teas, and other sweetened drinks, not presented as diet or non-caloric beverages), ASBs (beverages sweetened with non-nutritive (noncaloric) sweeteners such as sucralose or aspartame, including diet/sugar-free soft drinks, low-calorie beverages, non-calorie carbonated soft drinks, sports or energy drinks), and fruit juices (including apple, orange, grapefruit, and other 100% or pure fruit juices without added sugars or artificial sweeteners) (8, 3) the endpoints of interest were incidence of T2D (one or more classic symptoms [excessive thirst, polyuria, weight loss, or hunger], or fasting glucose of ≥ 126 mg/dl, or nonfasting glucose of ≥ 200 mg/dl or current use of hypoglycemic medication or self-reported T2D), hypertension (any use of antihypertensive medication taking or systolic or diastolic blood pressures greater than 140 or 90 mmHg or self-reported hypertension), CHD (nonfatal myocardial infarction or fatal CHD), stroke (rapid onset of focal neurological symptoms of presumed vascular origin or lasting > 24 h or resulting in death), and mortality (death from all the cause or CVD; more detailed definitions of diseases are shown in Supplementary Tables 3–8); (4) relative risks (RRs) with 95% confidence intervals (CIs) were available. The data with the longest follow-up period were selected when outcome data were presented at multiple time points. Literature search and further eligibility assessment was conducted by three authors (BL, NY, and LW) independently, with discrepancies resolved by discussion with another reviewer (LM).

### Data extraction and quality assessment

The following information was extracted from each study by three independent authors (BL, NY, and HJ): the first author, year of publication, geographical location, duration of the follow-up, cohort name, number of participants and cases, age, gender, beverage intake categories, method of dietary assessment, methods of outcome ascertainment, type and number of cases or deaths, covariates adjusted in the multivariable analysis, and the RRs with 95% CIs for each category of beverage. If studies provided more than one estimate, the RRs fully adjusted for confounding factors were extracted for analysis.

Study quality was assessed by the same three authors according to the Newcastle–Ottawa quality assessment scale (23). Studies scored 0–3, 4–6, and 7–9 were defined as low, moderate, and high quality, respectively.

To estimate the credibility of evidence for the association between drinks and risk of cardiometabolic diseases, the NutriGrade scoring system was used (24). The calculated score was grouped into four categorizations to interpret the certainty of evidence: very low (0– < 4 points), low (4– < 6 points), moderate (6– < 8 points) and high (≥  8 points).

### Data synthesis and statistical analysis

The combined risk estimates across studies were calculated by using random-effect models for the associations of SSBs, ASBs, and fruit juices with hypertension, T2D, CVD and mortality (25). The *I^2^* statistic (with *I*^2^ < 50% being low and *I*^2^ > 50% being apparent heterogeneity) was used to measure the magnitude of between-study heterogeneity. Meta-regression and subgroup analyses were conducted according to sex (men, women, or both), geographical locations (the United States, Europe, or Asia), follow-up year (< 10 years or ≥ 10 years), assessment method (FFQ, dietary history, or diet record), body mass index (BMI; adjusted or unadjusted) as well as the study quality (high or medium). There were relatively too few studies on the associations of fruit juice and hypertension/CHD/stroke/mortality and the association of ASBs and CHD/stroke to conduct meaningful subgroup analyses, thus, no further stratified analyses were conducted.

We assessed the potential dose–response meta-analyses between the categories of beverage consumption and the chronic diseases mentioned above by using restricted cubic splines with three knots located at 10, 50, and 90th percentiles of the distribution, which was proposed by Greenland and Longnecker (26) and Orsini et al. (27).

If the extreme category of one study was open-ended, it is assumed that its width was the same as that of the adjacent category (28, 29). For better comparison, different SSB, ASB, and fruit juice intakes like per drink, per day/per week or servings/cups were converted into ml/d according to the definition of the serving/cup in each study, or using estimates from studies in the same country if not specified in the article.

Sensitivity analyses in which one study at a time was excluded were performed to assess the robustness of the results and the influence of individual studies on heterogeneity. The significance or direction of the association did not significantly change when omitting one study at a time or excluding a category of studies, indicating the robustness of the results. Further sensitivity analyses were conducted for studies that controlled for important confounders including age, smoking, alcohol drinking, BMI, and physical activity. Potential publication bias was assessed by the application of funnel plots, Egger’ test, and Begg’s test at the *p* < 0.10 level of significance (30, 31). If potential publication bias was indicated, a trim and fill analysis was applied to evaluate the number of missing studies, and recalculation of the pooled relative risk was done after addition of those missing hypothetical studies. Statistical analyses were performed using Stata version 14.0 software (StataCorp, College Station, TX, United States).

## Results

### Literature search and study characteristics

**Figure 1:**
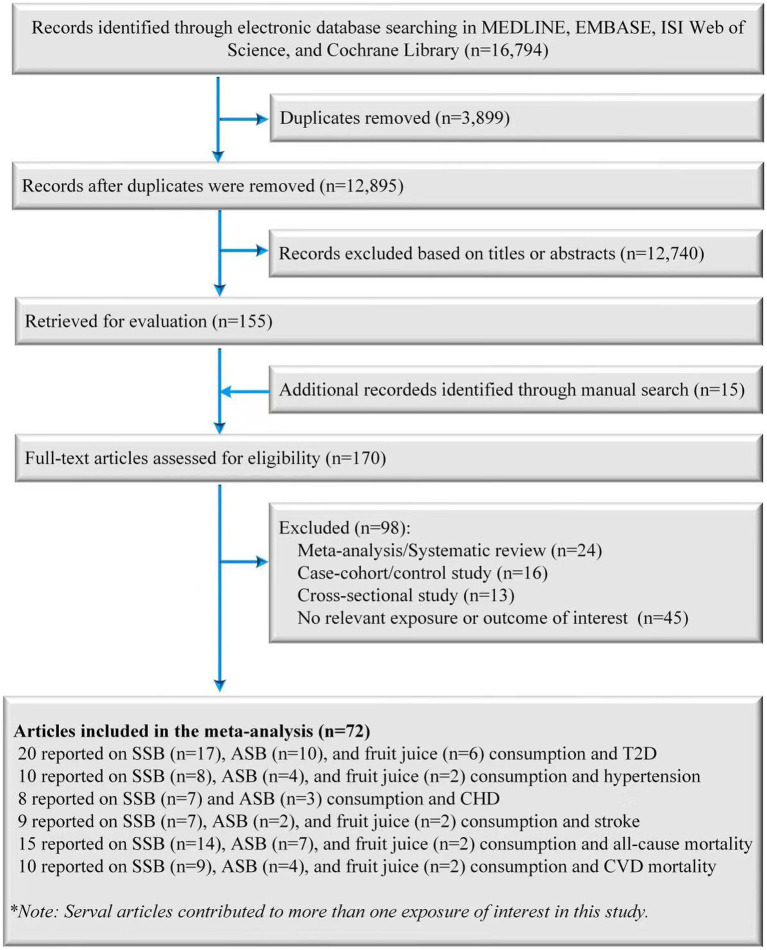
Screening and selection process of studies of SSB, ASB and fruit juice consumption and T2D, hypertension, CHD, stroke, and mortality risk. ASB, artificially sweetened beverage; CHD, coronary heart disease; SSB, sugar-sweetened beverage; T2D, type 2 diabetes.

A total of 12,910 records were retrieved from databases and manual searches after the removal of duplicates, of which 170 were identified for full-text review after preliminarily screening the abstracts and titles. After evaluating the full texts, 98 publications were excluded for the following reasons: (1) 24 records were excluded because they were meta-analyses or systematic reviews; (2) 16 records were excluded because they were case-cohort studies or case control studies; (3) 13 records were excluded because they were cross-sectional studies; (4) 45 records were excluded because the endpoint or exposure was not relevant to the purpose of the present study. Finally, 72 records (71 published original articles and one conference abstract) were included in the final analysis (Figure 1) (32–82).

The main characteristics of the selected studies are presented in Supplementary Tables 3–8. Eleven studies were conducted in Asia, 11 in Europe, and 46 in the United States. The follow-up period ranged from 4.0 to 38.0 years. Dietary assessment of 64 studies was performed using validated FFQs, and 8 studies used diet records. All the studies were adjusted with multivariable models. Most studies controlled for smoking (*n* = 70), physical activity (*n* = 70), age (*n* = 68), body mass index (*n* = 64), and alcohol drinking (*n* = 58). The quality scores of the studies were shown in Supplementary Tables 9–14, with 63 studies rated as high quality, and 9 studies as medium quality. According to the NutriGrade scoring system (Supplementary Tables 15–17), the credibility of evidence on the associations of SSB intake with T2D, CHD, stroke, and all-cause mortality was rated high. The same was true for the association between ASB intake and T2D. Evidence for other associations was of low to moderate quality.

### Association between beverages and T2D

**Figure 2:**
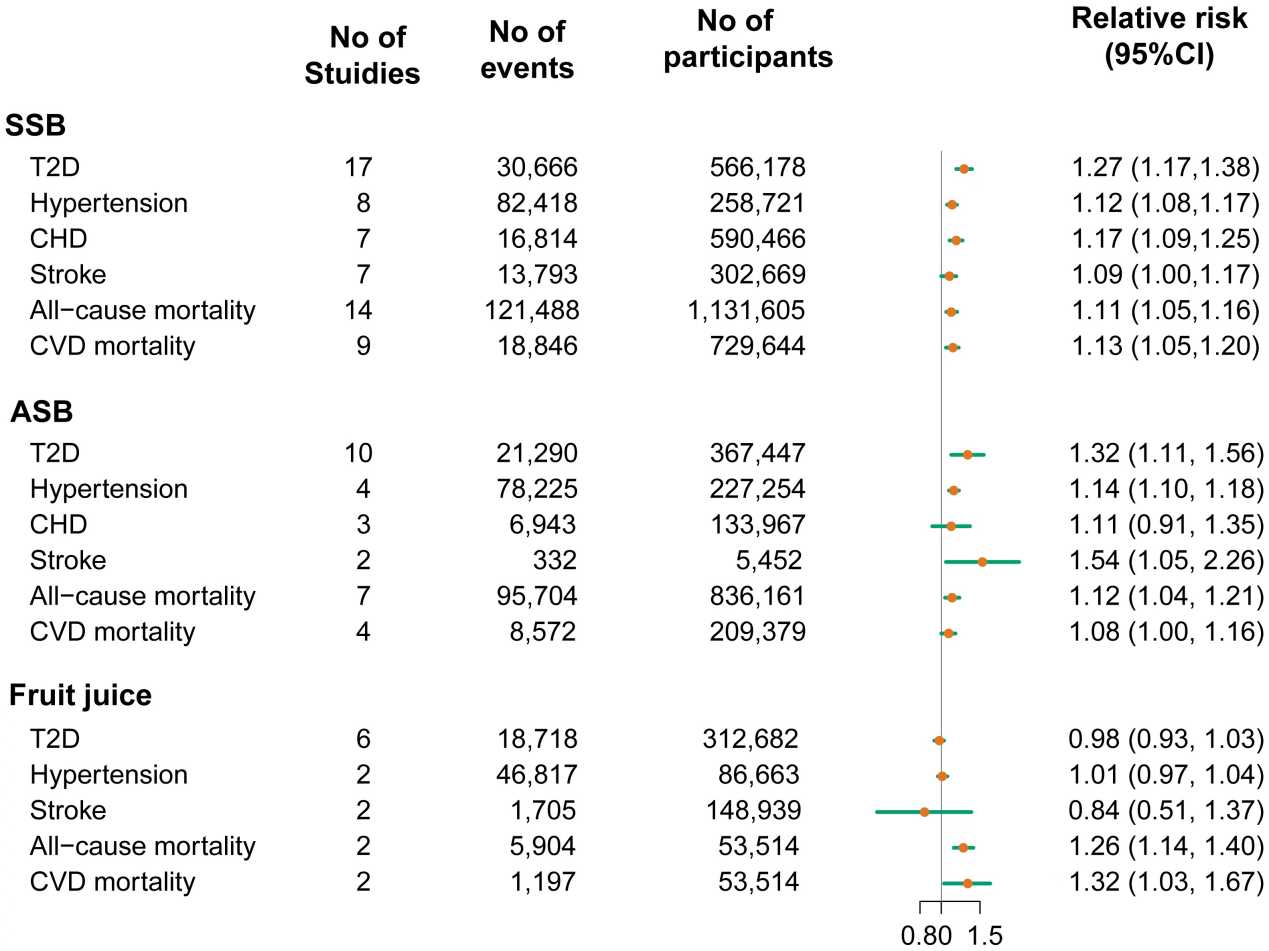
Pooled relative risks of type 2 diabetes, hypertension, coronary heart disease, stroke and mortality comparing the highest with the lowest categories of sugar-sweetened beverage, artificially sweetened beverage and fruit juice consumption. ASB, artificially sweetened beverage; CHD, coronary heart disease; CVD, cardiovascular disease; SSB, sugar-sweetened beverage; T2D, type 2 diabetes.

**Figure 3:**
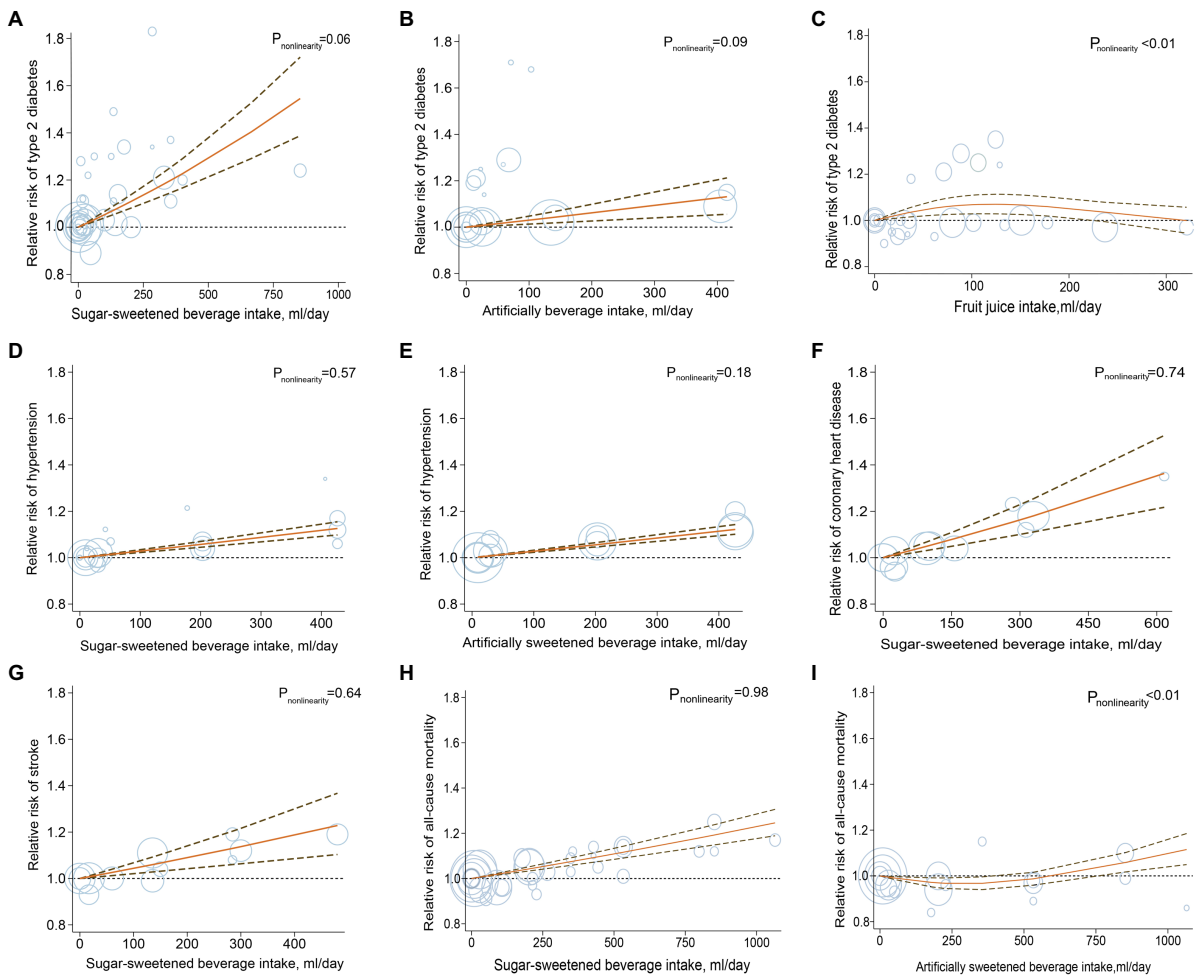
Dose–response analysis for curvilinear association of sugar-sweetened beverages, artificially sweetened beverages, and fruit juices with type 2 diabetes **(A–C)**, hypertension **(D,E)**, coronary heart disease **(F)**, stroke **(G)** and mortality **(H,I)**. Circles represent point estimates plotted over precision measures. The solid line and the dotted lines represent the estimated relative risks and their 95% confidence intervals.

A total of 20 studies with a total of 70,674 T2D cases in 1,246,307 participants investigated the associations between beverage consumption and T2D risk, including 17 studies of SSBs, 10 studies of ASBs, and 6 studies of fruit juices (32–51). The overall effect estimates of T2D comparing the highest to lowest categories were 1.27 for SSBs (95% CI: 1.17, 1.38; *P*_heterogeneity_ < 0.001; *I*^2^ = 69.2%; *n* = 17; Figure 2; Supplementary Figure 1), 1.32 for ASBs (95% CI: 1.11, 1.56, *P*_heterogeneity_ < 0.001; *I*^2^ = 92.7%; *n* = 10; Figure 2; Supplementary Figure 2), and 0.98 for fruit juices (95% CI: 0.93, 1.03; *P*_heterogeneity_ = 0.59; *I*^2^ = 0.0%; *n* = 6; Figure 2; Supplementary Figure 3). For the association between SSB and fruit juice intake and T2D, the pooled risk estimates were similar to those from the full analyses when study results were stratified by prespecified characteristics. In the analysis of ASB intake and T2D, when stratified by sex, the association was stronger among women than men. In the analysis stratified by the adjustment for BMI, a weaker but still significant association was detected among studies with adjustment for BMI (Supplementary Table 18). The dose–response meta-analyses found a linear association between SSB and ASB consumption and T2D risk (RR_SSB_: 1.20, 95% CI: 1.15, 1.25; *P*_nonlinearity_ = 0.06; RR_ASB_: 1.11, 95% CI: 1.05, 1.18; *P*_nonlinearity_ = 0.09; Figure 3). Indication for a nonlinear relationship between fruit juice intake and T2D was detected (*P*_nonlinearity_ = 0.001; Figure 3).

### Association between beverages and CVD

Twenty-seven studies were included in the analyses of beverage consumption and CVD risk. A total of 207,460 cases of hypertension (*n* = 10), 23,757 cases of CHD (*n* = 8), and 15,830 cases due to stroke (*n* = 9) in 1,395,468 participants were included in the present study (32, 33, 52–68). Compared with participants in the bottom category of SSBs, those in the top category had a 12% higher risk of hypertension (RR: 1.12; 95% CI: 1.08, 1.17; *P*_heterogeneity_ = 0.05; *I*^2^ = 50.3%; *n* = 8; Figure 2; Supplementary Figure 4). Similarly, higher ASB consumption was associated with a 14% increase in hypertension risk (RR: 1.14; 95% CI: 1.10, 1.18; *P*_heterogeneity_ = 0.06; *I*^2^ = 60.0%; *n* = 4; Figure 2; Supplementary Figure 5). A significant association was not detected between fruit juice consumption and hypertension risk (RR: 1.01; 95% CI: 0.97, 1.04; *P*_heterogeneity_ = 0.69; *I*^2^ = 0.0%; *n* = 2; Figure 2; Supplementary Figure 6).

When comparing the highest category with the lowest one, the risk of CHD was significantly higher by 17% for SSBs (RR: 1.17, 95% CI: 1.09, 1.25, *P*_heterogeneity_ = 0.83; *I*^2^ = 0.0%; *n* = 6; Figure 2; Supplementary Figure 7). However, ASB consumption was not significantly associated with CHD (RR: 1.11, 95% CI: 0.91, 1.35, *P*_heterogeneity_ = 0.05; *I*^2^ = 65.6%; *n* = 3; Figure 2; Supplementary Figure 8). For stroke, both SSBs and ASBs showed positive associations, with the pooled effect estimates of 1.09 (95% CI: 1.00, 1.17, *P*_heterogeneity_ = 0.38; *I*^2^ = 6.9%; *n* = 7; Figure 2; Supplementary Figure 9) and 1.54 (95% CI: 1.05, 2.26, *P*_heterogeneity_ = 0.28; *I*^2^ = 16.0%; *n* = 2; Figure 2; Supplementary Figure 10), respectively. Fruit juice consumption was not significantly associated with stroke (RR: 0.84, 95% CI: 0.51, 1.37, *P*_heterogeneity_ = 0.007; *I^2^* = 86.1%; n = 2; Figure 2; Supplementary Figure 11). The result of stratified analyses showed that the association between SSB consumption and hypertension incidence was more pronounced in cohort studies following up less than 10 years than in studies with a longer follow-up. Subgroup analyses stratified by sex showed that the association between ASBs and hypertension appeared to be stronger in women than in men (Supplementary Table 19). No source of heterogeneity was found in the subgroup analyses of the associations between SSB intake and CHD and stroke (Supplementary Table 20).

The dose–response analyses indicated that the risk of hypertension increased linearly with the increasing consumption of SSBs and ASBs; and per serving/d higher consumption of these beverages was associated with a 10% higher risk of hypertension (RR: 1.10, 95% CI: 1.08, 1.13, *P*_nonlinearity_ = 0.57; RR: 1.10, 95% CI: 1.08, 1.12, *P*_nonlinearity_ = 0.18; respectively; Figure 3). The dose–response analysis also found that the multivariate RR for one serving/d increment in SSB consumption was 1.20 (*P*_nonlinearity_ = 0.74; RR: 1.20; 95% CI: 1.12, 1.28; Figure 3) for CHD and 1.16 for stroke (RR: 1.16; 95% CI: 1.08, 1.26; *P*_nonlinearity_ = 0.64; Figure 3). Due to the limited numbers of included studies, evaluation for association or dose–response analysis between fruit juices as well as ASBs and risk of CHD or stroke was not performed.

### Association between beverages and mortality

Fifteen studies with 223,096 cases of all-cause mortality (*n* = 15) and 28,615 cases of mortality from CVD (*n* = 10) in 3,013,817 participants were included in the analyses of beverages and mortality (64, 69–82). The pooled RRs for the comparison of the highest to the lowest consumption category for all-cause mortality were 1.11 for SSBs (95% CI: 1.05, 1.16; *P*_heterogeneity_ < 0.001; *I*^2^ = 74.8%; *n* = 14; Figure 2; Supplementary Figure 12), 1.12 for ASBs (95% CI: 1.04, 1.21; *P*_heterogeneity_ < 0.001; *I^2^* = 81.9%; *n* = 7; Figure 2; Supplementary Figure 13) and 1.26 for fruit juices (95% CI: 1.14, 1.40; *P*_heterogeneity_ = 0.65; *I*^2^ = 0.0%; *n* = 2; Figure 2; Supplementary Figure 14). Evidence of statistically significant heterogeneity was observed in subgroups stratified for geographic locations for the association between consumption of SSBs, ASBs, and all-cause mortality, showing stronger associations in European studies compared with studies in the United States (Supplementary Table 21). Moreover, SSB, ASB, and fruit juice intake were positively associated with the risk of CVD mortality (RR _SSB_:1.13, 95% CI: 1.05, 1.20, *P*_heterogeneity_ = 0.32; *I^2^* = 14.0%; *n* = 9; Figure 2; Supplementary Figure 15; RR _ASB_: 1.08, 95% CI: 1.00, 1.16, *P*_heterogeneity_ = 0.15; *I^2^* = 43.8%; *n* = 4; Figure 2; Supplementary Figure 16; RR _fruit juice_:1.32, 95% CI: 1.03, 1.67, *P*_heterogeneity_ = 0.77; *I^2^* = 0.0%; *n* = 2; Figure 2; Supplementary Figure 17). No source of heterogeneity was identified in prespecified stratified analysis for the associations between consumption of SSBs, ASBs, and CVD mortality (Supplementary Table 22). The test for linear dose–response analysis revealed that all-cause mortality increased by 8% for each serving/d increase in SSB intake (RR: 1.08; 95% CI, 1.06, 1.09; *P*_nonlinearity_ = 0.98; Figure 3).

### Sensitivity analyses and publication bias

The sensitivity analyses did not materially alter the statistical significance or direction of the overall results, suggesting that the summary estimates were not strongly influenced by a specific study in the present study. In the sensitivity analysis restricted to studies that adjusted for the important factors associated with beverage intake and/or cardiometabolic disease, a weaker but still significant association was observed in general for these outcomes, except for the associations of SSB intake with hypertension and mortality risk, which showed stronger associations (Supplementary Tables 18–22). No significant publication bias was detected by Egger’s test (all *p* > 0.10), Begg’s test (all *p* > 0.10), or the visual inspection of the funnel plot (Supplementary Figure 18), except for the association of SSB intake and risk of T2D (Egger’s test *p* = 0.001). Six missing studies were imputed by using the trim-and-fill method and the magnitude of the pooled effect size incorporating these hypothetical studies was weaker than the original risk estimate, however, remained to be statistically significant (RR, 1.19; 95% CI: 1.10, 1.29; Supplementary Figure 19).

## Discussion

In the present study, increasing SSB and ASB intake was associated with a higher risk of cardiometabolic diseases and mortality. Furthermore, the increased risk was more pronounced for T2D compared with other cardiometabolic diseases. The results largely persisted in dose–response meta-analyses or subgroup analyses.

Results from the current study reinforce previous concerns regarding the adverse health effects of beverages and support the hypothesis that high dietary intake of SSBs and ASBs increase the risk of several major chronic diseases, which are compatible with and further extend the evidence from previous meta-analyses of prospective observational studies that also found positive associations (83–86). This study reinforces earlier evidence by including the data from several newly published large scale prospective epidemiological studies. For example, for the association between SSBs and CVD mortality, the present study involved approximately twice as many participants as the previous study (87). Similarly, analysis on SSBs and CHD relied on data from seven cohort studies that had about twice as many studies as the recent meta-analysis (88). Moreover, a wide range of subgroup and sensitivity analyses were carried out to assess the robustness of the associations between beverage exposures and cardiometabolic diseases and mortality.

Numbers of possible biological mechanisms might explain the positive associations between the consumption of SSBs and ASBs and risk of T2D and cardiometabolic disease. Consumption of SSBs and ASBs is associated with the risk of obesity, which is an independent risk factor for cardiometabolic disease (84, 89–92). For SSBs, another independent factor may arise from the large amount of rapidly absorbable carbohydrates, such as fructose added to SSBs. The biological mechanisms have been proposed by which large amounts of fructose in SSBs can increase biological pathways related to the occurrence of these diseases, including accelerating inflammation and promoting oxidative stress. In an animal model of fructose administration, Roglans et al. (93) demonstrated that fructose could reduce the activity of hepatic peroxisome proliferator-activated receptor α, which has been implicated in the pathogenesis of obesity and metabolic syndrome. A high-fructose diet in mice conducted in 2019 provoked detrimental effects in mitochondrial function including enhanced ROS production, the partial inhibition of oxidative phosphorylation, decreased complex I activity, and peroxidation of lipids (94). Fructose is also the only sugar that can increase blood uric acid concentrations. High serum uric acid levels may induce vascular oxidative stress, endothelial dysfunction, and reduction of nitric oxide production (95), which may partly explain the association between SSB consumption and cardiometabolic disease risk. Besides, some studies have found that liquid calories from SSBs elicited a weaker satiety response than solid calories, potentially leading to decreases in the compensation for liquid calories and increases in the participants’ dietary consumption of sugars and energy, which inhibits the activity of leptin and thus lead to subsequent energy intake, weight gain, and cardiometabolic morbidity (96, 97).

Although ASBs contain much fewer calories and sugars than SSBs, artificial sweeteners in ASBs are not necessarily biologically inert and may provoke inflammation, insulin resistance (98, 99), appetite dysregulation (100), and disruption of gut microbiota (20). Kim et al. (101) found infiltration of inflammatory cells and production of ROS in the liver and brain were increased in zebrafish fed with aspartame or saccharin. *In vivo*, 6-month saccharin administration in drinking water conducted in 2017 drove the development of liver inflammation in mice by altering the intestinal flora and its metabolic capacity (102). Additionally, the findings for ASBs need to be interpreted more cautiously due to reverse causation or residual confounding since for example, obesity, hypertension, and hypercholesterolemia preceded ASB intake in several studies or participants on these trajectories may choose to drink ASBs aiming at minimizing further weight gain and reducing blood pressure and blood fat levels (72, 74, 77, 103).

In the present study, fruit juice intake was not associated with T2D and hypertension risk. However, it should be noted that a significant association was observed between fruit juice intake and mortality risk, though there were only two studies included to analysis the association of fruit juice intake and mortality. Some cohort studies also have shown that 100% fruit juice, compared with fruit drinks, has neutral (104, 105) or protective (105, 106) associations with incident cardiometabolic disease. The protective association of fruit juices may be due to the vitamins and bioactive phytochemicals within the juices (107). Fruit juices contain fewer dietary fibers which reduce satiety and increase hunger, thereby resulting in increasing food intake (107, 108). Krishnasamy et al. (109) found processing apples to puree or juice reduced the sensation of fullness and satiety by speeding gastric emptying and decreasing postprandial intestinal volumes. Furthermore, 100% fruit juices contain free sugars that are quite similar to that of SSBs leading to the relatively high glycemic load values of fruit juices (110, 111). However, the nutrient content and composition in juices, such as vitamins, minerals, and phytochemicals, are related to components of the raw material and could be affected by the freshness of fruits and processing (112). Therefore, more research is needed to assess the effects of fruit juices on health outcomes.

The results should be interpreted in the context of several potential limitations. First, the definitions of SSBs, ASBs, and fruit juices varied across different studies, which could have resulted in misclassification bias of the exposure variable. However, because of the prospective design for included studies, any misclassification was likely to be nondifferential, which might tend to bias results toward the null. Second, participants with higher levels of beverage intake tended to have other unhealthy lifestyle habits that may increase the risk of cardiometabolic diseases and mortality (52). Although most included studies have adjusted for potential confounding factors, we cannot entirely rule out the possibility of unmeasured confounding or residual confounding that might affect the observed associations. Third, other foods in the diet might interact with the beverage, although we were unable to assess any additive or synergistic effects between beverage consumption and dietary factors in relation to chronic disorder in the present meta-analysis. Further studies are required to examine the potential interactions. Finally, some evidence of possible publication bias was detected between SSB intake and the risk of T2D. Although trim and fill analysis yielded a similar magnitude of effect estimates, the possibility of such bias cannot be completely ruled out.

## Conclusion

Our meta-analysis of existing prospective evidence indicated that consumption of SSBs and ASBs was associated with a higher risk of cardiometabolic diseases and mortality. Our findings support the current recommendations of limiting the intake of SSBs to facilitate the prevention of cardiometabolic diseases, and also add further evidence that suggests ASBs and fruit juices are not necessarily healthier alternatives to SSBs.

## Data availability statement

The original contributions presented in the study are included in the article/Supplementary material, further inquiries can be directed to the corresponding authors.

## Author contributions

LM, DW, MM, BL, NY, and HJ contributed to the refinement of study concept and design. BL, NY, and LW performed the literature search and selected the studies. BL, NY, and HJ performed data extraction from relevant studies and prepared initial figures and data analysis. BL and NY wrote the first draft. BL, NY, HJ, MC, MW, LW, BM, ZL, JS, YF, MA, CL, FC, MM, DW, and LM critically revised subsequent drafts and finalized the final version. MM, DW, and LM are the corresponding authors of the article and assume responsibility for the completeness of the data and the accuracy of the data analysis. All authors contributed to the article and approved the submitted version.

## Funding

This work is supported by grants from the National Natural Science Foundation of China (NSFC-82022062; NSFC-81973025); Nutrition Science Research Foundation of BY-HEALTH (TY0181101); New-star Plan of Science and Technology of Shaanxi Province (2015LJXX-07); and the Fundamental Research Funds for the Central Universities (qngz2016004; xzy032019008). The funders of the study had no role in the study design, implementation, analysis, decision to publish, or reparation of the manuscript.

## Conflict of interest

The authors declare that the research was conducted in the absence of any commercial or financial relationships that could be construed as a potential conflict of interest.

## Publisher’s note

All claims expressed in this article are solely those of the authors and do not necessarily represent those of their affiliated organizations, or those of the publisher, the editors and the reviewers. Any product that may be evaluated in this article, or claim that may be made by its manufacturer, is not guaranteed or endorsed by the publisher.

## References

[1] Global Food Research Program. (2022). Sugary drink taxes around the world. Chapel Hill. Available at: https://www.globalfoodresearchprogram.org/wp-content/uploads/2022/11/Sugary_Drink_Tax_maps_2022_11.pdf

[2] Backholer K Blake M Vandevijvere S. Sugar-sweetened beverage taxation: an update on the year that was 2017. Public Health Nutr. (2017) 20:3219–24. doi: 10.1017/S1368980017003329, PMID: 29160766 PMC10261626

[3] Guthrie JF Morton JF. Food sources of added sweeteners in the diets of Americans. J Am Diet Assoc. (2000) 100:43–51. doi: 10.1016/S0002-8223(00)00018-3 10646004

[4] Azaïs-Braesco V Sluik D Maillot M Kok F Moreno LA. A review of total & added sugar intakes and dietary sources in Europe. Nutr J. (2017) 16:6. doi: 10.1186/s12937-016-0225-2, PMID: 28109280 PMC5251321

[5] Louie JC Moshtaghian H Rangan AM Flood VM Gill TP. Intake and sources of added sugars among Australian children and adolescents. Eur J Nutr. (2016) 55:2347–55. doi: 10.1007/s00394-015-1041-8, PMID: 26377592

[6] Sánchez-Pimienta TG Batis C Lutter CK Rivera JA. Sugar-sweetened beverages are the Main sources of added sugar intake in the Mexican population. J Nutr. (2016) 146:S1888–96. doi: 10.3945/jn.115.220301, PMID: 27511931

[7] U.S. Department of Agriculture, U.S. Department of Health and Human Services. Dietary Guidelines for Americans, 2020–2025. 9th ed. Washington, DC: U.S. Department of Agriculture, U.S. Department of Health and Human Services (2020).

[8] U.S. Department of Health and Human Services, U.S. Department of Agriculture. Dietary Guidelines for Americans, 2015–2020. 8th ed. Washington, DC: U.S. Department of Health and Human Services, U.S. Department of Agriculture (2015).

[9] Cassady BA Considine RV Mattes RD. Beverage consumption, appetite, and energy intake: what did you expect? Am J Clin Nutr. (2012) 95:587–93. doi: 10.3945/ajcn.111.025437, PMID: 22258267 PMC3278240

[10] Zhu Z He Y Wang Z He X Zang J Guo C. The associations between sugar-sweetened beverage intake and cardiometabolic risks in Chinese children and adolescents. Pediatr Obes. (2020) 15:e12634. doi: 10.1111/ijpo.12634, PMID: 32196990

[11] Malik VS Hu FB. Fructose and Cardiometabolic health: what the evidence from sugar-sweetened beverages tells us. J Am Coll Cardiol. (2015) 66:1615–24. doi: 10.1016/j.jacc.2015.08.025, PMID: 26429086 PMC4592517

[12] Gardner C Wylie-Rosett J Gidding SS Steffen LM Johnson RK Reader D. Nonnutritive sweeteners: current use and health perspectives: a scientific statement from the American Heart Association and the American Diabetes Association. Diabetes Care. (2012) 35:1798–808. doi: 10.2337/dc12-9002, PMID: 22778165 PMC3402256

[13] Johnson RK Lichtenstein AH Anderson CAM Carson JA Després JP Hu FB. Low-calorie sweetened beverages and cardiometabolic health: a science advisory from the American Heart Association. Circulation. (2018) 138:e126–40. doi: 10.1161/CIR.0000000000000569, PMID: 30354445

[14] Diabetes Canada. Diabetes Canada 2018 clinical practice guidelines for the prevention and management of diabetes in Canada. Can J Diabetes. (2018) 42:S1–S326. 24070926 10.1016/j.jcjd.2013.01.009

[15] Diabetes UK (2018). Evidence-based nutrition guidelines for the prevention and management of diabetes. Available at: https://diabetes-resources-production.s3.eu-west-1.amazonaws.com/resources-s3/2018-03/1373_Nutrition%20guidelines_0.pdf. Accessed July 31, 2021.

[16] American Diabetes Association. 5. Facilitating behavior change and well-being to improve health outcomes: standards of medical care in Diabetes-2020. Diabetes Care. (2020) 43:S48–65. doi: 10.2337/dc20-S005, PMID: 31862748

[17] Lee JJ Khan TA McGlynn N Malik VS Hill JO Leiter LA. Relation of change or substitution of low-and no-calorie sweetened beverages with cardiometabolic outcomes: a systematic review and meta-analysis of prospective cohort studies. Diabetes Care. (2022) 45:1917–30. doi: 10.2337/dc21-2130, PMID: 35901272 PMC9346984

[18] McGlynn ND Khan TA Wang L Zhang R Chiavaroli L Au-Yeung F. Association of low-and no-calorie sweetened beverages as a replacement for sugar-sweetened beverages with body weight and cardiometabolic risk: a systematic review and meta-analysis. JAMA Netw Open. (2022) 5:e222092. doi: 10.1001/jamanetworkopen.2022.2092, PMID: 35285920 PMC9907347

[19] U.S. Department of Agriculture, U.S. Department of Health and Human Services. USDA National Nutrient Database for Standard Reference, Release 25. (2012) Available from http://www.ars.usda.gov/Services/docs.htm?docid=8964 [Accessed December 12, 2020].

[20] Suez J Korem T Zeevi D Zilberman-Schapira G Thaiss CA Maza O. Artificial sweeteners induce glucose intolerance by altering the gut microbiota. Nature. (2014) 514:181–6. doi: 10.1038/nature13793 25231862

[21] Mace OJ Affleck J Patel N Kellett GL. Sweet taste receptors in rat small intestine stimulate glucose absorption through apical GLUT2. J Physiol. (2007) 582:379–92. doi: 10.1113/jphysiol.2007.130906, PMID: 17495045 PMC2075289

[22] Page MJ McKenzie JE Bossuyt PM Boutron I Hoffmann TC Mulrow CD. The PRISMA 2020 statement: an updated guideline for reporting systematic reviews. BMJ. (2021) 372:n71. doi: 10.1136/bmj.n71, PMID: 33782057 PMC8005924

[23] Wells GA Shea BJ O’Connell D Peterson J Tugwell P (2000). The Newcastle-Ottawa scale (NOS) for assessing the quality of non randomized studies in meta-analyses. Available at http://www.ohri.ca/programs/clinical_epidemiology/oxford.asp. Accessed June 1, 2020.

[24] Schwingshackl L Knuppel S Schwedhelm C Hoffmann G Missbach B Stelmach-Mardas M. Perspective: Nutrigrade: a scoring system to assess and judge the meta-evidence of randomized controlled trials and cohort studies in nutrition research. Adv Nutr. (2016) 7:994–1004. doi: 10.3945/an.116.013052, PMID: 28140319 PMC5105044

[25] Cochran WG. The combination of estimates from different experiments. Biometrics. (1954) 10:101–29. doi: 10.2307/3001666

[26] Greenland S Longnecker MP. Methods for trend estimation from summarized dose-response data, with applications to meta-analysis. Am J Epidemiol. (1992) 135:1301–9. doi: 10.1093/oxfordjournals.aje.a116237, PMID: 1626547

[27] Orsini N Bellocco R Greenland S. Generalized least squares for trend estimation of summarized dose–response data. Stata J. (2006) 6:40–57. doi: 10.1177/1536867X0600600103

[28] Greenwood DC Threapleton DE Evans CE Cleghorn CL Nykjaer C Woodhead C. Association between sugar-sweetened and artificially sweetened soft drinks and type 2 diabetes: systematic review and dose-response meta-analysis of prospective studies. Br J Nutr. (2014) 112:725–34. doi: 10.1017/S0007114514001329, PMID: 24932880

[29] Bechthold A Boeing H Schwedhelm C Hoffmann G Knüppel S Iqbal K. Food groups and risk of coronary heart disease, stroke and heart failure: a systematic review and dose-response meta-analysis of prospective studies. Crit Rev Food Sci Nutr. (2019) 59:1071–90. doi: 10.1080/10408398.2017.1392288, PMID: 29039970

[30] Egger M Davey Smith G Schneider M Minder C. Bias in meta-analysis detected by a simple, graphical test. BMJ. (1997) 315:629–34. doi: 10.1136/bmj.315.7109.629, PMID: 9310563 PMC2127453

[31] Begg CB Mazumdar M. Operating characteristics of a rank correlation test for publication bias. Biometrics. (1994) 50:1088–101. doi: 10.2307/2533446, PMID: 7786990

[32] Nettleton JA Lutsey PL Wang Y Lima JA Michos ED Jr Jacobs DR. Diet soda intake and risk of incident metabolic syndrome and type 2 diabetes in the multi-ethnic study of atherosclerosis (MESA). Diabetes Care. (2009) 32:688–94. doi: 10.2337/dc08-1799, PMID: 19151203 PMC2660468

[33] Auerbach BJ Littman AJ Tinker L Larson J Krieger J Young B. Associations of 100% fruit juice versus whole fruit with hypertension and diabetes risk in postmenopausal women: results from the Women's Health Initiative. Prev Med. (2017) 105:212–8. doi: 10.1016/j.ypmed.2017.08.031, PMID: 28888824 PMC5653413

[34] Paynter NP Yeh HC Voutilainen S Schmidt MI Heiss G Folsom AR. Coffee and sweetened beverage consumption and the risk of type 2 diabetes mellitus: the atherosclerosis risk in communities study. Am J Epidemiol. (2006) 164:1075–84. doi: 10.1093/aje/kwj323, PMID: 16982672

[35] Pereira MP Parker ED Folsom AR. Intake of sugar sweetened beverages, fruit juice, and incidence of type 2 diabetes: a prospective study of postmenopausal women. Diabetes. (2005) 54:A258.

[36] Montonen J Järvinen R Knekt P Heliövaara M Reunanen A. Consumption of sweetened beverages and intakes of fructose and glucose predict type 2 diabetes occurrence. J Nutr. (2007) 137:1447–54. doi: 10.1093/jn/137.6.1447, PMID: 17513405

[37] Palmer JR Boggs DA Krishnan S Hu FB Singer M Rosenberg L. Sugar-sweetened beverages and incidence of type 2 diabetes mellitus in African American women. Arch Intern Med. (2008) 168:1487–92. doi: 10.1001/archinte.168.14.1487, PMID: 18663160 PMC2708080

[38] de Koning L Malik VS Rimm EB Willett WC Hu FB. Sugar-sweetened and artificially sweetened beverage consumption and risk of type 2 diabetes in men. Am J Clin Nutr. (2011) 93:1321–8. doi: 10.3945/ajcn.110.007922, PMID: 21430119 PMC3095502

[39] Pan A Malik VS Schulze MB Manson JE Willett WC Hu FB. Plain-water intake and risk of type 2 diabetes in young and middle-aged women. Am J Clin Nutr. (2012) 95:1454–60. doi: 10.3945/ajcn.111.032698, PMID: 22552035 PMC3349456

[40] Bhupathiraju SN Pan A Malik VS Manson JAE Willett WC van Dam RM. Caffeinated and caffeine-free beverages and risk of type 2 diabetes. Am J Clin Nutr. (2013) 97:155–66. doi: 10.3945/ajcn.112.048603, PMID: 23151535 PMC3522135

[41] Fagherazzi G Vilier A Saes Sartorelli D Lajous M Balkau B Clavel-Chapelon F. Consumption of artificially and sugar-sweetened beverages and incident type 2 diabetes in the etude Epidémiologique auprès des femmes de la Mutuelle Générale de l'Education Nationale–European prospective investigation into cancer and nutrition cohort. Am J Clin Nutr. (2013) 3:517–23. doi: 10.3945/ajcn.112.050997 23364017

[42] Eshak ES Iso H Mizoue T Inoue M Noda M Tsugane S. Soft drink, 100% fruit juice, and vegetable juice intakes and risk of diabetes mellitus. Clin Nutr. (2013) 32:300–8. doi: 10.1016/j.clnu.2012.08.003, PMID: 22917499

[43] Sakurai M Nakamura K Miura K Takamura T Yoshita K Nagasawa SY. Sugar-sweetened beverage and diet soda consumption and the 7-year risk for type 2 diabetes mellitus in middle-aged Japanese men. Eur J Nutr. (2014) 53:1137–8. doi: 10.1007/s00394-014-0681-4, PMID: 24633756

[44] O'Connor L Imamura F Lentjes MA Khaw KT Wareham NJ Forouhi NG. Prospective associations and population impact of sweet beverage intake and type 2 diabetes, and effects of substitutions with alternative beverages. Diabetologia. (2015) 58:1474–83. doi: 10.1007/s00125-015-3572-1, PMID: 25944371 PMC4473082

[45] Huang M Quddus A Stinson L Shikany JM Howard BV Kutob RM. Artificially sweetened beverages, sugar-sweetened beverages, plain water, and incident diabetes mellitus in postmenopausal women: the prospective Women's Health Initiative observational study. Am J Clin Nutr. (2017) 106:614–22. doi: 10.3945/ajcn.116.145391, PMID: 28659294 PMC5525115

[46] Papier K D'Este C Bain C Banwell C Seubsman S Sleigh A. Consumption of sugar-sweetened beverages and type 2 diabetes incidence in Thai adults: results from an 8-year prospective study. Nutr Diabetes. (2017) 7:e283. doi: 10.1038/nutd.2017.27, PMID: 28628126 PMC5519187

[47] Gardener H Moon YP Rundek T Elkind MSV Sacco RL. Diet soda and sugar-sweetened soda consumption in relation to incident diabetes in the northern Manhattan study. Curr Dev Nutr. (2018) 2:2005002. doi: 10.1093/cdn/nzy008, PMID: 29955723 PMC5998368

[48] Hirahatake KM Jacobs DR Shikany JM Jiang L Wong ND Steffen LM. Cumulative intake of artificially sweetened and sugar-sweetened beverages and risk of incident type 2 diabetes in young adults: the coronary artery risk development in Young adults (CARDIA) study. Am J Clin Nutr. (2019) 110:733–41. doi: 10.1093/ajcn/nqz154, PMID: 31374564 PMC6736196

[49] Odegaard AO Koh WP Arakawa K Yu MC Pereira MA. Soft drink and juice consumption and risk of physician-diagnosed incident type 2 diabetes: the Singapore Chinese health study. Am J Epidemiol. (2010) 171:701–8. doi: 10.1093/aje/kwp452, PMID: 20160170 PMC2842218

[50] Scheffers FR Wijga AH Verschuren WMM van der Schouw YT Sluijs I Smit HA. Pure fruit juice and fruit consumption are not associated with incidence of type 2 diabetes after adjustment for overall dietary quality in the European prospective investigation into cancer and nutrition-Netherlands (EPIC-NL) study. J Nutr. (2020) 150:1470–7. doi: 10.1093/jn/nxz340, PMID: 31943054 PMC7269751

[51] Torres-Ibarra L Rivera-Paredez B Hernández-López R Canto-Osorio F Sánchez-Romero LM López-Olmedo N. Regular consumption of soft drinks is associated with type 2 diabetes incidence in Mexican adults: findings from a prospective cohort study. Nutr J. (2020) 19:126. doi: 10.1186/s12937-020-00642-9, PMID: 33218344 PMC7678283

[52] Dhingra R Sullivan L Jacques PF Wang TJ Fox CS Meigs JB. Soft drink consumption and risk of developing cardiometabolic risk factors and the metabolic syndrome in middle-aged adults in the community. Circulation. (2007) 116:480–8. doi: 10.1161/CIRCULATIONAHA.107.689935, PMID: 17646581

[53] Duffey KJ Gordon-Larsen P Steffen LM Jacobs DR Jr Popkin BM. Drinking caloric beverages increases the risk of adverse cardiometabolic outcomes in the coronary artery risk development in Young adults (CARDIA) study. Am J Clin Nutr. (2010) 92:954–9. doi: 10.3945/ajcn.2010.29478, PMID: 20702604 PMC2937591

[54] Cohen L Curhan G Forman J. Association of sweetened beverage intake with incident hypertension. J Gen Intern Med. (2012) 27:1127–34. doi: 10.1007/s11606-012-2069-6, PMID: 22539069 PMC3515007

[55] Sayon-Orea C Martinez-Gonzalez MA Gea A Alonso A Pimenta AM Bes-Rastrollo M. Baseline consumption and changes in sugar-sweetened beverage consumption and the incidence of hypertension: the SUN project. Clin Nutr. (2015) 34:1133–40. doi: 10.1016/j.clnu.2014.11.010, PMID: 25481680

[56] Kwak JH Jo G Chung HK Shin MJ. Association between sugar-sweetened beverage consumption and incident hypertension in Korean adults: a prospective study. Eur J Nutr. (2019) 58:1009–17. doi: 10.1007/s00394-018-1617-1, PMID: 29372311

[57] Siqueira JH Pereira TSS Moreira AD Diniz MFHS Velasquez-Melendez G Fonseca MJM. Consumption of sugar-sweetened soft drinks and risk of metabolic syndrome and its components: results of the ELSA-Brasil study (2008-2010 and 2012-2014). J Endocrinol Investig. (2022) 46:159–71. doi: 10.1007/s40618-022-01895-3, PMID: 35963981

[58] Fung TT Malik V Rexrode KM Manson JE Willett WC Hu FB. Sweetened beverage consumption and risk of coronary heart disease in women. Am J Clin Nutr. (2009) 89:1037–42. doi: 10.3945/ajcn.2008.27140, PMID: 19211821 PMC2667454

[59] de Koning L Malik VS Kellogg MD Rimm EB Willett WC Hu FB. Sweetened beverage consumption, incident coronary heart disease, and biomarkers of risk in men. Circulation. (2012) 125:1735–1741, s1. doi: 10.1161/CIRCULATIONAHA.111.067017, PMID: 22412070 PMC3368965

[60] Gardener H Rundek T Markert M Wright CB Elkind MS Sacco RL. Diet soft drink consumption is associated with an increased risk of vascular events in the northern Manhattan study. J Gen Intern Med. (2012) 27:1120–6. doi: 10.1007/s11606-011-1968-2, PMID: 22282311 PMC3514985

[61] Eshak ES Iso H Kokubo Y Saito I Yamagishi K Inoue M. Soft drink intake in relation to incident ischemic heart disease, stroke, and stroke subtypes in Japanese men and women: the Japan public health Centre-based study cohort I. Am J Clin Nutr. (2012) 96:1390–7. doi: 10.3945/ajcn.112.037903, PMID: 23076619

[62] Warfa K Drake I Wallström P Engström G Sonestedt E. Association between sucrose intake and acute coronary event risk and effect modification by lifestyle factors: Malmö diet and cancer cohort study. Br J Nutr. (2016) 116:1611–20. doi: 10.1017/S0007114516003561, PMID: 27774913

[63] Scheffers FR Boer JMA Verschuren WMM Verheus M van der Schouw YT Sluijs I. Pure fruit juice and fruit consumption and the risk of CVD: the European prospective investigation into cancer and nutrition-Netherlands (EPIC-NL) study. Br J Nutr. (2019) 121:351–9. doi: 10.1017/S0007114518003380, PMID: 30428938 PMC6390400

[64] Keller A O'Reilly EJ Malik V Buring JE Andersen I Steffen L. Substitution of sugar-sweetened beverages for other beverages and the risk of developing coronary heart disease: results from the Harvard pooling project of diet and coronary disease. Prev Med. (2019) 131:105970. doi: 10.1016/j.ypmed.2019.105970, PMID: 31883872

[65] Pacheco LS Lacey JV Jr Martinez ME Lemus H Araneta MRG Sears DD. Sugar-sweetened beverage intake and cardiovascular disease risk in the California teachers study. J Am Heart Assoc. (2020) 9:e014883. doi: 10.1161/JAHA.119.014883.8, PMID: 32397792 PMC7660873

[66] Johansson A Acosta S Mutie PM Sonestedt E Engström G Drake I. Components of a healthy diet and different types of physical activity and risk of atherothrombotic ischemic stroke: a prospective cohort study. Front Cardiovasc Med. (2022) 9:993112. doi: 10.3389/fcvm.2022.993112, PMID: 36312237 PMC9614044

[67] Bernstein AM de Koning L Flint AJ Rexrode M Willett WC. Soda consumption and the risk of stroke in men and women. Am J Clin Nutr. (2012) 95:1190–9. doi: 10.3945/ajcn.111.030205, PMID: 22492378 PMC3325840

[68] Joshipura KJ Ascherio A Manson JE Stampfer MJ Rimm EB Speizer FE. Fruit and vegetable intake in relation to risk of ischemic stroke. JAMA. (1999) 282:1233–9. doi: 10.1001/jama.282.13.1233 10517425

[69] Pase MP Himali JJ Beiser AS Aparicio HJ Satizabal CL Vasan RS. Sugar-and artificially sweetened beverages and the risks of incident stroke and dementia: a prospective cohort study. Stroke. (2017) 48:1139–46. doi: 10.1161/STROKEAHA.116.016027, PMID: 28428346 PMC5405737

[70] Odegaard AO Koh WP Yuan JM Pereira MA. Beverage habits and mortality in Chinese adults. J Nutr. (2015) 145:595–604. doi: 10.3945/jn.114.200253, PMID: 25733477 PMC4336537

[71] Barrington WE White E. Mortality outcomes associated with intake of fast-food items and sugar-sweetened drinks among older adults in the vitamins and lifestyle (VITAL) study. Public Health Nutr. (2016) 19:3319–26. doi: 10.1017/S1368980016001518, PMID: 27338763 PMC10271132

[72] Malik VS Li Y Pan A de Koning L Schernhammer E Willett WC. Long-term consumption of sugar-sweetened and artificially sweetened beverages and risk of mortality in US adults. Circulation. (2019) 139:2113–25. doi: 10.1161/CIRCULATIONAHA.118.037401, PMID: 30882235 PMC6488380

[73] Paganini-Hill A Kawas CH Corrada MM. Non-alcoholic beverage and caffeine consumption and mortality: the leisure world cohort study. Prev Med. (2007) 44:305–10. doi: 10.1016/j.ypmed.2006.12.011, PMID: 17275898 PMC2034362

[74] Mullee A Romaguera D Pearson-Stuttard J Viallon V Stepien M Freisling H. Association between soft drink consumption and mortality in 10 European countries. JAMA Intern Med. (2019) 179:1479–90. doi: 10.1001/jamainternmed.2019.2478, PMID: 31479109 PMC6724165

[75] Collin LJ Judd S Safford M Vaccarino V Welsh JA. Association of Sugary Beverage Consumption with Mortality Risk in US adults: a secondary analysis of data from the REGARDS study. JAMA Netw Open. (2019) 2:e193121. doi: 10.1001/jamanetworkopen.2019.3121, PMID: 31099861 PMC6537924

[76] Ramne S Alves Dias J González-Padilla E Olsson K Lindahl B Engström G. Association between added sugar intake and mortality is nonlinear and dependent on sugar source in 2 Swedish population-based prospective cohorts. Am J Clin Nutr. (2019) 109:411–23. doi: 10.1093/ajcn/nqy268, PMID: 30590448

[77] Anderson JJ Gray SR Welsh P Mackay DF Celis-Morales CA Lyall DM. The associations of sugar-sweetened, artificially sweetened and naturally sweet juices with all-cause mortality in 198, 285 UK biobank participants: a prospective cohort study. BMC Med. (2020) 18:97. doi: 10.1186/s12916-020-01554-5, PMID: 32326961 PMC7181499

[78] Vyas A Rubenstein L Robinson J Seguin RA Vitolins MZ Kazlauskaite R. Diet drink consumption and the risk of cardiovascular events: a report from the Women's Health Initiative. J Gen Intern Med. (2015) 30:462–8. doi: 10.1007/s11606-014-3098-0, PMID: 25515135 PMC4371001

[79] Zhang YB Chen JX Jiang YW Xia PF Pan A. Association of sugar-sweetened beverage and artificially sweetened beverage intakes with mortality: an analysis of US National Health and nutrition examination survey. Eur J Nutr. (2021) 60:1945–55. doi: 10.1007/s00394-020-02387-x, PMID: 32945955

[80] Pacheco LS Lacey JV Jr Martinez ME Lemus H Sears DD Araneta MRG. Association between sugar-sweetened beverage intake and mortality risk in women: the California teachers study. J Acad Nutr Diet. (2022) 122:320–333.e6. doi: 10.1016/j.jand.2021.08.099, PMID: 34389488 PMC9001025

[81] Zhang Z Zeng X Li M Zhang T Li H Yang H. A prospective study of fruit juice consumption and the risk of overall and cardiovascular disease mortality. Nutrients. (2022) 14:2127. doi: 10.3390/nu14102127, PMID: 35631268 PMC9144949

[82] Naomi ND Brouwer-Brolsma EM Buso MEC Soedamah-Muthu SS Harrold JA Halford JCG. Association of sweetened beverages consumption with all-cause mortality risk among Dutch adults: the lifelines cohort study (the SWEET project). Eur J Nutr. (2022) 62:797–806. doi: 10.1007/s00394-022-03023-6, PMID: 36271197 PMC9589708

[83] Yin J Zhu Y Malik V Li X Peng X Zhang FF. Intake of sugar-sweetened and low-calorie sweetened beverages and risk of cardiovascular disease: a meta-analysis and systematic review. Adv Nutr. (2021) 12:89–101. doi: 10.1093/advances/nmaa084, PMID: 32696948 PMC7850046

[84] Qin P Li Q Zhao Y Chen Q Sun X Liu Y. Sugar and artificially sweetened beverages and risk of obesity, type 2 diabetes mellitus, hypertension, and all-cause mortality: a dose-response meta-analysis of prospective cohort studies. Eur J Epidemiol. (2020) 35:655–71. doi: 10.1007/s10654-020-00655-y, PMID: 32529512

[85] Neelakantan N Park SH Chen GC van Dam RM. Sugar-sweetened beverage consumption, weight gain, and risk of type 2 diabetes and cardiovascular diseases in Asia: a systematic review. Nutr Rev. (2021) 80:50–67. doi: 10.1093/nutrit/nuab010, PMID: 33855443

[86] Meng Y Li S Khan J Dai Z Li C Hu X. Sugar-and artificially sweetened beverages consumption linked to type 2 diabetes, cardiovascular diseases, and all-cause mortality: a systematic review and dose-response meta-analysis of prospective cohort studies. Nutrients. (2021) 13:2636. doi: 10.3390/nu13082636, PMID: 34444794 PMC8402166

[87] Pan B Ge L Lai H Wang Q Wang Q Zhang Q. Association of soft drink and 100% fruit juice consumption with all-cause mortality, cardiovascular diseases mortality, and cancer mortality: a systematic review and dose-response meta-analysis of prospective cohort studies. Crit Rev Food Sci Nutr. (2021) 62:8908–19. doi: 10.1080/10408398.2021.1937040, PMID: 34121531

[88] Santos LP Gigante DP Delpino FM Maciel AP Bielemann RM. Sugar sweetened beverages intake and risk of obesity and cardiometabolic diseases in longitudinal studies: a systematic review and meta-analysis with 1.5 million individuals. Clin Nutr ESPEN. (2022) 51:128–42. doi: 10.1016/j.clnesp.2022.08.021, PMID: 36184197

[89] Zheng H Ji H Fan K Xu H Huang Y Zheng Y. Targeting gut microbiota and host metabolism with Dendrobium officinale dietary fiber to prevent obesity and improve glucose homeostasis in diet-induced obese mice. Mol Nutr Food Res. (2022) 66:e2100772. doi: 10.1002/mnfr.202100772, PMID: 35225418

[90] Lumeng CN Bodzin JL Saltiel AR. Obesity induces a phenotypic switch in adipose tissue macrophage polarization. J Clin Invest. (2007) 117:175–84. doi: 10.1172/JCI29881, PMID: 17200717 PMC1716210

[91] Seravalle G Grassi G. Obesity and hypertension. Pharmacol Res. (2017) 122:1–7. doi: 10.1016/j.phrs.2017.05.013 28532816

[92] Ortega FB Lavie CJ Blair SN. Obesity and cardiovascular disease. Circ Res. (2016) 118:1752–70. doi: 10.1161/CIRCRESAHA.115.306883 27230640

[93] Roglans N Vilà L Farré M Alegret M Sánchez RM Vázquez-Carrera M. Impairment of hepatic Stat-3 activation and reduction of PPARalpha activity in fructose-fed rats. Hepatology. (2007) 45:778–88. doi: 10.1002/hep.21499, PMID: 17326204

[94] García-Berumen CI Ortiz-Avila O Vargas-Vargas MA del Rosario-Tamayo BA Guajardo-López C Saavedra-Molina A. The severity of rat liver injury by fructose and high fat depends on the degree of respiratory dysfunction and oxidative stress induced in mitochondria. Lipids Health Dis. (2019) 18:78. doi: 10.1186/s12944-019-1024-5, PMID: 30927921 PMC6441141

[95] Balakumar M Raji L Prabhu D Sathishkumar C Prabu P Mohan V. High-fructose diet is as detrimental as high-fat diet in the induction of insulin resistance and diabetes mediated by hepatic/pancreatic endoplasmic reticulum (ER) stress. Mol Cell Biochem. (2016) 423:93–104. doi: 10.1007/s11010-016-2828-5, PMID: 27699590

[96] Rolls BJ Kim S Fedoroff IC. Effects of drinks sweetened with sucrose or aspartame on hunger, thirst and food intake in men. Physiol Behav. (1990) 48:19–26. doi: 10.1016/0031-9384(90)90254-2 2236270

[97] Malik VS Popkin BM Bray GA Després JP Hu FB. Sugar-sweetened beverages, obesity, type 2 diabetes mellitus, and cardiovascular disease risk. Circulation. (2010) 121:1356–64. doi: 10.1161/CIRCULATIONAHA.109.876185, PMID: 20308626 PMC2862465

[98] Sánchez-Tapia M Martínez-Medina J Tovar AR Torres N. Natural and artificial sweeteners and high fat diet modify differential taste receptors, insulin, and TLR4-mediated inflammatory pathways in adipose tissues of rats. Nutrients. (2019) 11:880. doi: 10.3390/nu11040880, PMID: 31010163 PMC6520815

[99] Margolskee RF Dyer J Kokrashvili Z Salmon KSH Ilegems E Daly K. T1R3 and gustducin in gut sense sugars to regulate expression of Na+−glucose cotransporter 1. Proc Natl Acad Sci U S A. (2017) 104:15075–80. doi: 10.1073/pnas.0706678104, PMID: 17724332 PMC1986615

[100] Ludwig DS. Artifcially sweetened beverages: cause for concern. JAMA. (2009) 302:2477–8. doi: 10.1001/jama.2009.1822 19996404

[101] Kim JY Seo J Cho KH. Aspartame-fed zebrafish exhibit acute deaths with swimming defects and saccharin-fed zebrafish have elevation of cholesteryl ester transfer protein activity in hypercholesterolemia. Food Chem Toxicol. (2011) 49:2899–905. doi: 10.1016/j.fct.2011.08.001, PMID: 21855599

[102] Bian X Tu P Chi L Gao B Ru H Lu K. Saccharin induced liver inflammation in mice by altering the gut microbiota and its metabolic functions. Food Chem Toxicol. (2017) 107:530–9. doi: 10.1016/j.fct.2017.04.045, PMID: 28472674 PMC5647777

[103] Mossavar-Rahmani Y Kamensky V Manson JE Silver B Rapp SR Haring B. Artificially sweetened beverages and stroke, coronary heart disease, and all-cause mortality in the Women's Health Initiative. Stroke. (2019) 50:555–62. doi: 10.1161/STROKEAHA.118.023100, PMID: 30802187 PMC6538252

[104] Xi B Li S Liu Z Tian H Yin X Huai P. Intake of fruit juice and incidence of type 2 diabetes: a systematic review and meta-analysis. PLoS One. (2014) 9:e93471. doi: 10.1371/journal.pone, PMID: 24682091 PMC3969361

[105] D'Elia L Dinu M Sofi F Volpe M Strazzullo P SINU Working Group. 100% fruit juice intake and cardiovascular risk: a systematic review and meta-analysis of prospective and randomised controlled studies. Eur J Nutr. (2021) 60:2449–67. doi: 10.1007/s00394-020-02426-7, PMID: 33150530 PMC8275541

[106] Oude Griep LM Geleijnse JM Kromhout D Ocké MC Verschuren WM. Raw and processed fruit and vegetable consumption and 10-year coronary heart disease incidence in a population-based cohort study in the Netherlands. PLoS One. (2010) 5:e13609. doi: 10.1371/journal.pone.0013609, PMID: 21049053 PMC2963618

[107] Clemens R Drewnowski A Ferruzzi MG Toner CD Welland D. Squeezing fact from fiction about 100% fruit juice. Adv Nutr. (2015) 6:236S–43S. doi: 10.3945/an.114.007328, PMID: 25770266 PMC4352186

[108] Haber GB Heaton KW Murphy D Burroughs LF. Depletion and disruption of dietary fibre. Effects on satiety, plasma-glucose, and serum-insulin. Lancet. (1977) 2:679–82. doi: 10.1016/s0140-6736(77)90494-9, PMID: 71495

[109] Krishnasamy S Lomer MCE Marciani L Hoad CL Pritchard SE Paul J. Processing apples to puree or juice speeds gastric emptying and reduces postprandial intestinal volumes and satiety in healthy adults. J Nutr. (2020) 150:2890–9. doi: 10.1093/jn/nxaa191, PMID: 32805050

[110] Pepin A Stanhope KL Imbeault P. Are fruit juices healthier than sugar-sweetened beverages? Rev. Nutr. (2019) 11:1006. doi: 10.3390/nu11051006, PMID: 31052523 PMC6566863

[111] University of Sydney (n.d). Online glycemic index database. Available at: http://www.glycemicindex.com. Accessed May 2, 2011.

[112] Zheng J Zhou Y Li S Zhang P Zhou T Xu DP. Effects and mechanisms of fruit and vegetable juices on cardiovascular diseases. Int J Mol Sci. (2017) 18:555. doi: 10.3390/ijms18030555, PMID: 28273863 PMC5372571

