# Supplementary material for: Consumption of sugar sweetened beverages, artificially sweetened beverages and fruit juices and risk of type 2 diabetes, hypertension, cardiovascular disease, and mortality: A meta-analysis
Source: Front Nutr. 2023 Mar 15;10:1019534. doi: 10.3389/fnut.2023.1019534 (PMC10050372; doi:10.3389/fnut.2023.1019534)
Supplement: Supplementary file 1 [file Data_Sheet_1.docx]

**Online-Only Supplemental Material**

**Supplementary tables**

**Supplementary Table 1.PRISMA Checklist**

| **Section and Topic** | **Item #** | **Checklist item** | **Location where item is reported** |
| --- | --- | --- | --- |
| **TITLE** | | |  |
| Title | 1 | Identify the report as a systematic review. | Page 1 |
| **ABSTRACT** | | |  |
| Abstract | 2 | See the PRISMA 2020 for Abstracts checklist. | Page 3 |
| **INTRODUCTION** | | |  |
| Rationale | 3 | Describe the rationale for the review in the context of existing knowledge. | Page 5 |
| Objectives | 4 | Provide an explicit statement of the objective(s) or question(s) the review addresses. | Page 6 |
| **METHODS** | | |  |
| Eligibility criteria | 5 | Specify the inclusion and exclusion criteria for the review and how studies were grouped for the syntheses. | Page 7 |
| Information sources | 6 | Specify all databases, registers, websites, organisations, reference lists and other sources searched or consulted to identify studies. Specify the date when each source was last searched or consulted. | Page 6 |
| Search strategy | 7 | Present the full search strategies for all databases, registers and websites, including any filters and limits used. | Page 6; Supplemental Table 2 |
| Selection process | 8 | Specify the methods used to decide whether a study met the inclusion criteria of the review, including how many reviewers screened each record and each report retrieved, whether they worked independently, and if applicable, details of automation tools used in the process. | Page 7 |
| Data collection process | 9 | Specify the methods used to collect data from reports, including how many reviewers collected data from each report, whether they worked independently, any processes for obtaining or confirming data from study investigators, and if applicable, details of automation tools used in the process. | Page 7 |
| Data items | 10a | List and define all outcomes for which data were sought. Specify whether all results that were compatible with each outcome domain in each study were sought (e.g. for all measures, time points, analyses), and if not, the methods used to decide which results to collect. | Supplemental Table 3-8 |
|  | 10b | List and define all other variables for which data were sought (e.g. participant and intervention characteristics, funding sources). Describe any assumptions made about any missing or unclear information. | No report |
| Study risk of bias assessment | 11 | Specify the methods used to assess risk of bias in the included studies, including details of the tool(s) used, how many reviewers assessed each study and whether they worked independently, and if applicable, details of automation tools used in the process. | Page 9 |
| Effect measures | 12 | Specify for each outcome the effect measure(s) (e.g. risk ratio, mean difference) used in the synthesis or presentation of results. | Page 8 |
| Synthesis methods | 13a | Describe the processes used to decide which studies were eligible for each synthesis (e.g. tabulating the study intervention characteristics and comparing against the planned groups for each synthesis (item #5)). | No report |
|  | 13b | Describe any methods required to prepare the data for presentation or synthesis, such as handling of missing summary statistics, or data conversions. | No report |
|  | 13c | Describe any methods used to tabulate or visually display results of individual studies and syntheses. | Page 8 |
|  | 13d | Describe any methods used to synthesize results and provide a rationale for the choice(s). If meta-analysis was performed, describe the model(s), method(s) to identify the presence and extent of statistical heterogeneity, and software package(s) used. | Page 9 |
|  | 13e | Describe any methods used to explore possible causes of heterogeneity among study results (e.g. subgroup analysis, meta-regression). | Page 8,9 |
|  | 13f | Describe any sensitivity analyses conducted to assess robustness of the synthesized results. | Page 9 |
| Reporting bias assessment | 14 | Describe any methods used to assess risk of bias due to missing results in a synthesis (arising from reporting biases). | Page 9 |
| Certainty assessment | 15 | Describe any methods used to assess certainty (or confidence) in the body of evidence for an outcome. | Page 8 |
| **RESULTS** | | |  |
| Study selection | 16a | Describe the results of the search and selection process, from the number of records identified in the search to the number of studies included in the review, ideally using a flow diagram. | Page 10; Figure 1 |
|  | 16b | Cite studies that might appear to meet the inclusion criteria, but which were excluded, and explain why they were excluded. | No report |
| Study characteristics | 17 | Cite each included study and present its characteristics. | Page 10; Supplemental Table 3-8 |
| Risk of bias in studies | 18 | Present assessments of risk of bias for each included study. | Page 11-13 |
| Results of individual studies | 19 | For all outcomes, present, for each study: (a) summary statistics for each group (where appropriate) and (b) an effect estimate and its precision (e.g. confidence/credible interval), ideally using structured tables or plots. | Page 11-13; Supplementary Figure 1-16 |
| Results of syntheses | 20a | For each synthesis, briefly summarise the characteristics and risk of bias among contributing studies. | Page 11-13; Supplementary Figure 17 |
|  | 20b | Present results of all statistical syntheses conducted. If meta-analysis was done, present for each the summary estimate and its precision (e.g. confidence/credible interval) and measures of statistical heterogeneity. If comparing groups, describe the direction of the effect. | Figure 2 |
|  | 20c | Present results of all investigations of possible causes of heterogeneity among study results. | Page 11-13; Supplementary Table 18-22 |
|  | 20d | Present results of all sensitivity analyses conducted to assess the robustness of the synthesized results. | Page 11-13 |
| Reporting biases | 21 | Present assessments of risk of bias due to missing results (arising from reporting biases) for each synthesis assessed. | Page 11-13; Supplementary Figure 16. |
| Certainty of evidence | 22 | Present assessments of certainty (or confidence) in the body of evidence for each outcome assessed. | Page 10; Supplemental Table 15-17 |
| **DISCUSSION** | | |  |
| Discussion | 23a | Provide a general interpretation of the results in the context of other evidence. | Page 13 |
|  | 23b | Discuss any limitations of the evidence included in the review. | Page 16,17 |
|  | 23c | Discuss any limitations of the review processes used. | Page 16,17 |
|  | 23d | Discuss implications of the results for practice, policy, and future research. | Page 17 |
| **OTHER INFORMATION** | | |  |
| Registration and protocol | 24a | Provide registration information for the review, including register name and registration number, or state that the review was not registered. | Page 6 |
|  | 24b | Indicate where the review protocol can be accessed, or state that a protocol was not prepared. | Page 6 |
|  | 24c | Describe and explain any amendments to information provided at registration or in the protocol. | No report |
| Support | 25 | Describe sources of financial or non-financial support for the review, and the role of the funders or sponsors in the review. | Page 18 |
| Competing interests | 26 | Declare any competing interests of review authors. | Page 18 |
| Availability of data, code and other materials | 27 | Report which of the following are publicly available and where they can be found: template data collection forms; data extracted from included studies; data used for all analyses; analytic code; any other materials used in the review. | No report |

**Supplementary Table 2. Search strategy**

| 1. “sugar-sweetened beverages” |
| --- |
| 2. “sugar beverages” |
| 3. “sweetened beverage” |
| 4. “sweet drink” |
| 5. “sugary drink” |
| 6. “non-alcoholic drinks” |
| 7. “soft drink” |
| 8. “beverage” |
| 9. “sport drink” |
| 10. “cold drink” |
| 11. “carbonated beverage” |
| 12. “carbonated soft drink” |
| 13. “carbonated sugar-sweetened beverages” |
| 14. “energy drink” |
| 15. “artificially sweetened beverage” |
| 16. “fruit drinks” |
| 17. “energy and vitamin water drinks” |
| 18. “fruit punch” |
| 19. “lemonade” |
| 20. “soda” |
| 21. “soda-pop” |
| 22. “other fruit drinks” |
| 1. “fruit juice” |
| 24. (1 OR 2 OR 3 OR 4 OR 5 OR 6 OR 7 OR 8 OR 9 OR 10 OR 11 OR 12 OR 13 OR 14 OR 15 OR 16 OR 17 OR 18 OR 19 OR 20 OR 21 OR 22 OR 23) |
| 25. “hypertension” |
| 26. “HBP” |
| 27. “high blood pressure” |
| 28. “blood pressure” |
| 29. “cardiovascular disease” |
| 30. “vascular diseases” |
| 31. “cardiovascular” |
| 32. “coronary heart disease” |
| 33. “ischemic heart disease” |
| 34. “angina” |
| 35. “myocardial infarction” |
| 36. “carotid artery diseases” |
| 37. “heart” |
| 38. “cerebrovascular accident” |
| 39. “intracranial hemorrhages” |
| 40. “stroke” |
| 41. “cerebrovascular disorders” |
| 42. “brain ischemia” |
| 43. “intracranial hemorrhages” |
| 44. “intracranial embolism and thrombosis” |
| 45. “vascular dementia” |
| 46. “diabetes mellitus” |
| 47. “metabolic diseases” |
| 48. “metabolic syndrome” |
| 49. “hyperglycemia” |
| 50. “type 2 diabetes” |
| 51. “impaired glucose” |
| 52. “impaired fasting insulin” |
| 53. “glucose” |
| 54. “insulin” |
| 55. “mortality” |
| 56. “all-cause mortality” |
| 57. “total mortality” |
| 58. “death” |
| 59. “fatal” |
| 60. “survival” |
| 61. “sudden death” |
| 62. “ determination of death” |
| 63. “cause of death” |
| 64. “hospital mortality” |
| 65. “near-death experience” |
| 66. (25 OR 26 OR 27 OR 28 OR 29 OR 30 OR 31 OR 32 OR 33 OR 34 OR 35 OR 36 OR 37 OR 38 OR 39 OR 40 OR 41 OR 42 OR 43 OR 44 OR 45 OR 46 OR 47 OR 48 OR 49 OR 50 OR 51 OR 52 OR 53 OR 54 OR 55 OR 56 OR 57 OR 58 OR 59 OR 60 OR 61 OR 62 OR 63 OR 64 OR 65) |
| 67. “cohorts” |
| 68. “prospective studies” |
| 69. “follow-up studies” |
| 70. “longitudinal” |
| 71. “follow-up” |
| 72. “relative risk” |
| 73. “population-based” |
| 74. “odds ratio” |
| 75. “hazard ratio” |
| 76. “incidence rate ratio” |
| 77. (67 OR 68 OR 69 OR 70 OR 71 OR 72 OR 73 OR 74 OR 75 OR 76) |
| 78. 24 AND 66 AND 77 |

**Supplementary Table 3. Summary of prospective studies on beverage and type 2 diabetes (n=20)**

| **Author,**  **publication year,**  **country** | **Characteristics of the study** | | | **Characteristics of the participant** | | | **Characteristics of the exposure** | | | **Characteristics of the outcome** | | | **Adjustment for confounding factors** | **Study quality^*^** |
| --- | --- | --- | --- | --- | --- | --- | --- | --- | --- | --- | --- | --- | --- | --- |
|  | **Study name** | **Baseline survey year** | **Follow up**  **(year)** | **No.** | **Age range (year)** | **Men (%)** | **Assay method** | **Exposure** | **Definition** | **Ascertainment method** | **Definition** | **Cases (n)** |  |  |
| Mark A. Pereira et al, 2005, USA  (postmenopausal women) | Iowa WHS | 1986 | 11.0 | 31,489 | 55-69 | 0.0 | FFQ | SSB | Soda and other sugar-based drinks | Self-report | No report | 1,561 | Age, smoking, alcohol drinking, education, BMI, physical activity, waist-hip ratio, estrogen use, dietary energy intake, fatty acids, cereal fiber, milk, and coffee intake | High |
| Nina P. Paynter et al, 2006,  USA | ARIC | 1987-89 | 9.0  (maximum) | 12,204 | 45-64 | 44.4 | FFQ | SSB | Fruit punch, nondiet soda, and orange or grapefruit juice | Self-report,  Biomarkers | Fasting glucose of ≥126 mg/dl, nonfasting glucose of ≥200 mg/dl, current use of hypoglycemic medication, or a positive response to the question, “Has a doctor ever told you that you had diabetes (sugar in the blood)?” | 1,437 | Age, race, smoking, alcohol drinking, education, BMI, physical activity, waist-hip ratio, family history of diabetes and hypertension, dietary energy intake, and dietary fiber intake | High |
| Jukka Montonen et al, 2007, Finland | FMCHES | 1967-72 | 12.0 | 4,304 | 40-60 | 53.1 | FFQ | SSB | No report | Records | The medical certificates of all the cases were checked and all met the WHO diagnostic criteria for type 2 diabetes | 177 | Age, sex, geographic area, smoking, BMI, physical activity, blood pressure, history of infarction, angina pectoris and cardiac failure, family history of diabetes, dietary energy intake, prudent dietary pattern score, conservative pattern score, and serum cholesterol | High |
| Julie R. Palmer et al, 2008, USA | BWHS | 1995 | 10.0 | 43,960 | 21-69 | 0.0 | FFQ | SSB | Regular soft drinks (not diet soda) fruit juice orange juice or grapefruit  juice not other fruit juices, fortified fruit drinks, Kool-Aid | Self-report | Reported diabetes on any of the follow-up questionnaires and had not previously reported a diagnosis of diabetes. | 2,713 | Age, smoking, physical activity, education, family history of diabetes, dietary other types of drinks, red meat, processed meats, cereal fiber, coffee intake, and glycemic index | High |
| Jennifer A. Nettleton et al, 2009, USA | MESA | 2000-02 | 5.8  (median) | 5,011 | 45-84 | 47.4 | FFQ | ASB | Diet soft drinks, unsweetened mineral water | Self-report,  Biomarkers | Self-reported type 2 diabetes, fasting glucose >126 mg/dl at any examination, or use of hypoglycemic medication | 413 | Age, sex, study site, race, smoking, education, BMI, waist circumference, physical activity, dietary energy intake, and supplement use | High |
| Andrew O. Odegaard et al, 2010, Singapore | SCHS | 1993-98 | 5.7  (mean) | 43,580 | 45-74 | 42.9 | FFQ | SSB | Soft drinks such as Coca-Cola (The Coca-Cola Company, Atlanta, Georgia) and 7UP (Dr. Pepper Snapple Group, Plano, Texas) | Self-report | Symptoms, diagnostic tests, and hypoglycemic therapy during a telephone interview | 2,273 | Age, sex, interview year, smoking, alcohol drinking, education, BMI, weight gain continuously, physical activity, dietary energy intake, saturated fat intake, dietary fiber intake, dairy intake, juice or soft drink intake depending on model, and coffee intake | High |
| Lawrence de Koning et al, 2011, USA | HPFS | 1986 | > 20 | 40,389 | 40-75 | 100.0 | FFQ | SSB, ASB | SSB: caffeinated colas, caffeine-free colas, other carbonated sugar-sweetened beverages, and noncarbonated sugar-sweetened beverages (fruit punches, lemonades, or other fruit drinks)  ASB: caffeinated, caffeine-free, and noncarbonated low-calorie beverages | Records | One or more classic symptoms (excessive thirst, polyuria, weight loss, or hunger) plus fasting plasma glucose concentrations 7.8 mmol/L or random plasma glucose concentrations 11.1 mmol/L; 2 elevated plasma glucose concentrations on different occasions (fasting concentrations 7.8 mmol/L, random plasma glucose concentrations 11.1 mmol/L, and/or concentrations 11.1 mmol/L after 2 h shown by oral-glucose-tolerance testing) in the absence of symptoms; or treatment with hypoglycemic medication (insulin or oral hypoglycemic agent). | 2,680 | Age, smoking, alcohol drinking, physical activity, diuretic use, history of hypertension and hypercholesterolemia, family history of type 2 diabetes, and multivitamin use | High |
| An Pan et al, 2012, USA | NHS II | 1991 | 18.0 | 82,902 | 26-45 | 0.0 | FFQ | SSB, ASB | SSB: Coke, Pepsi, or other cola with sugar; caffeine-free Coke, Pepsi, or other cola with sugar; other carbonated beverages with sugar; and Hawaiian Punch, lemonade, or other noncarbonated  fruit drinks  ASB: low-calorie cola with caffeine, low-calorie caffeinefree cola, and other low-calorie beverages | Self-report | One or more classic symptoms (excessive thirst, polyuria, weight loss, or hunger) plus fasting plasma glucose concentrations 7.8 mmol/L or random plasma glucose concentrations 11.1 mmol/L; 2 elevated plasma glucose concentrations on different occasions (fasting concentrations 7.8 mmol/L, random plasma glucose concentrations 11.1 mmol/L, and/or concentrations 11.1 mmol/L after 2 h shown by oral-glucose-tolerance testing) in the absence of symptoms; or treatment with hypoglycemic medication (insulin or oral hypoglycemic agent). | 2,718 | Age, race, smoking, alcohol drinking, BMI, physical activity, hormone use, oral contraceptive use, menopausal status, family history of diabetes, and Alternative Healthy Eating Index | High |
| Shilpa N Bhupathiraju et al, 2012, USA | NHS | 1984 | 24.0 | 74,749 | 30-55 | 0.0 | FFQ | SSB, ASB | SSB: Coke, Pepsi, or other cola with sugar; caffeine-free Coke, Pepsi, or other cola with sugar; other carbonated beverages with sugar; and Hawaiian Punch, lemonade, or other noncarbonated  fruit drinks  ASB: low-calorie cola with caffeine, low-calorie caffeinefree cola, and other low-calorie beverages | Self-report | One or more classic symptoms (excessive thirst, polyuria, weight loss, or hunger) plus fasting plasma glucose concentrations 7.8 mmol/L or random plasma glucose concentrations 11.1 mmol/L; 2 elevated plasma glucose concentrations on different occasions (fasting concentrations 7.8 mmol/L, random plasma glucose concentrations 11.1 mmol/L, and/or concentrations 11.1 mmol/L after 2 h shown by oral-glucose-tolerance testing) in the absence of symptoms; or treatment with hypoglycemic medication (insulin or oral hypoglycemic agent). | 7,370 | Smoking, alcohol drinking, BMI, weight change, physical activity, postmenopausal hormone use, history of hypertension and hypercholesterolemia, family history of diabetes, dietary energy intake, other beverage intake, Alternate Healthy Eating Index, and adherence to a low-calorie diet | Medium |
| Guy Fagherazzi et al, 2013, France | E3N | 1993 | 14.0 | 66,118 | 52.6 | 0.0 | FFQ | SSB, ASB, Fruit juice | Soda or water with added fruit Syrup, 100%fruit juice soda or fruit drinks  were sugar or artificially sweetened | Records | fasting glucose concentration >7.0 mmol/L or random glucose concentration >11.1 mmol/L) or if women reported taking diabetes drugs or their last values of fasting glucose or glycated  hemoglobin concentrations were reported to be >7.0 mmol/L or >7% respectively | 1,369 | Smoking, alcohol drinking, education, BMI, physical activity, hormone replacement therapy, antidiabetic drugs use, history of hypertension and hypercholesterolemia, family history of diabetes, dietary energy intake, omega-3 fatty acid intake, carbohydrate, coffee, fruit, vegetables, processed-meat intake, and dietary pattern | Medium |
| Ehab S. Eshak et al, 2013, Japan | JPHC | 1990 | 10.0 | 27,585 | 40-59 | 44.0 | FFQ | SSB, Fruit juice | SSB:cola, flavored juices, and non 100% fruit juices  Fruit juice: 100% fruit juices | Self-report | The World Health Organization (1985) criteria, or a high casual plasma glucose level (≥11mmol/l), or use of diabetic medication (insulin or oral hypoglycaemic agent) | 824 | Age, smoking, alcohol drinking, education, job, BMI, physical activity, history of hypertension, family history of diabetes mellitus, dietary energy intake, coffee, green tea, magnesium, calcium, vitamin D, rice, and fiber intake | High |
| M. Sakurai et al, 2014, Japan | Occupational cohort | 2003 | 5.5  (mean) | 2,037 | 35-55 | 100.0 | FFQ | SSB, ASB | SSB: regular soft drinks, sugar-sweetened soda, and sports drinks, excluding 100 % fruit juice  ASB: non-calorie carbonated soft drinks | Biomarkers | A fasting  plasma glucose concentration >126 mg/dL; or HbA1c value >6.5 %; and treatment with insulin or oral  hypoglycemic agent | 170 | Age, smoking, alcohol drinking, BMI, physical activity, diet treatment for chronic disease, history of hypertension and dyslipidemia, family history of diabetes, dietary energy intake, fiber, SSB (for diet soda), diet soda (for SSB), fruit juice, vegetable juice and coffee intake | High |
| Laura O’Connor et al, 2015, UK | EPIC  -Norfolk | 1993-97 | 10.8 | 24,653 | 40-79 | 45.3 | 7-day diet record | SSB, ASB, Fruit juice | SSB: soft drinks,  sweetened-milk beverages, sweetened tea/coffee  ASB: diet/sugar-free soft drinks, sports/energy drinks and Squashes  Fruit juice: 100% fruit juice and nectars | Records | Selfreport of doctor-diagnosed diabetes, selfreport of diabetes-specific medication | 847 | Age, sex, smoking, alcohol drinking, education, social class, season, BMI, waist circumference, physical activity, family history of diabetes, dietary energy intake, and other sweet beverage intake | Medium |
| Mengna Huang  et al, 2017, USA  (postmenopausal women) | WHI OS | 1993-98 | 8.4  (mean) | 64,850 | 50-79 | 0.0 | FFQ | SSB, ASB | SSB: regular soft drinks (not diet)fruit juice, and  fruit drinks  ASB: diet drinks | Self-report | Use pills for diabetes or insulin shots for diabetes | 4,675 | Age, race, smoking, alcohol drinking, education, marital status, income, insurance status, BMI, BMI change, physical activity, sitting time, waist-to-hip ratio, systolic blood pressure, antihypertensive use, antihyperlipidemic use, hormone replacement therapy use, cardiovascular, hysterectomy, family history of diabetes, dietary energy intake, SSB (for ASB), ASB (for SSB), Alternate Healthy Eating Index, glycemic  load, and glycemic index | High |
| Brandon J. Auerbach  et al, 2017, USA  (postmenopausal women） | WHI | 1993-98 | 7.8  (mean) | 114,219 | 50-79 | 0.0 | FFQ | Fruit juice | 100% orange and grapefruit juices, and all other 100% fruit juice types | Self-report | Self-reported incident diabetes；medication to treat diabetes | 11,488 | Age, race, smoking, education, BMI, physical activity, hormone replacement therapy status, study arm and dietary energy intake | High |
| K Papier et al, 2017, Thailand | TCS | 2005 | 8.0 | 39,175 | 22-40 | 45.0 | FFQ | SSB | Any carbonated  sweetened beverage or soda | Self-report | Confirmed diagnosis from a doctor | 695 | Age, residence, smoking, alcohol drinking, education, income, BMI, physical activity, history of hypertension, fruit and vegetable intake and deep-fried food intake | Medium |
| Hannah Gardener et al, 2018, USA | NOMAS | 1993-2001 | 11  (mean) | 2,019 | 69.0 | 36.0 | FFQ | SSB, ASB | SSB: regular soft drinks  ASB: diet soft drinks | Self-report | Diagnosed with diabetes or high blood sugar or take insulin or oral hypoglycemics | 368 | Age, sex, race, smoking, alcohol drinking, BMI, physical activity, history of hypertension and hypercholesterolemia, dietary energy intake, and Mediterranean diet | High |
| Kristin M Hirahatake  et al,2019,  USA | CARDIA | 1985-86 | 30.0 | 4,719 | 18-30 | 45.3 | FFQ | SSB, ASB | SSB: sugar-sweetened soft drinks and fruit drinks  ASB:soft drinks and fruit drinks sweetened with non-nutritive (noncaloric) sweeteners | Biomarkers | Use of diabetes  medication, a fasting blood glucose concentration of ≥7 mmol/L (126 mg/dL), 2-hour post-challenge glucose ≥11.1 mmol/L (200 mg/dL), and/or a HbA1c ≥48 mmol/mol (6.5%) | 680 | Age, sex, race, CARDIA center, smoking, education, BMI, weight change, physical activity, dietary energy intake, SSB (for ASB), ASB (for SSB), dieting behavior, and Mediterranean diet score | High |
| Floor R Scheffers et al, 2020, Netherlands | EPIC-NL | 1993-1997 | 14.6 (mean) | 36,147 | 49.0  (mean) | 25.5 | FFQ | fruit juice | Both freshly squeezed and bottled juice and never contains added sugars, artificial sweeteners | self-report, records | Self-report whether the strip had turned purple after 10 s, indicating glucosuria, validated by consulting the general practitioner or the pharmacist | 1,477 | Age, sex, smoking, alcohol drinking, education, BMI, physical activity, waist circumference, family history of diabetes, DHD15-index, coffee, sugar-sweetened beverages, and fruit | Medium |
| Leticia Torres-Ibarra et al,2020,  Mexico | HWCS | 2004-07 | 6.7  (median) | 1,445 | 19-94 | 24.4 | FFQ | SSB | Cola soft drinks and flavored carbonated soft drinks | Self-report,  Biomarkers | self-report of physician-diagnosed type 2 diabetes, new use of hypoglycemic medication, or fasting glucose > 126 mg/ dL during the examination | 109 | Age, sex, smoking, alcohol drinking, education, BMI, abdominal obesity, physical activity, history of hypertension, family history of diabetes, and dietary energy intake | High |

ASB, artificially sweetened beverage; ARIC, Atherosclerosis Risk in Communities Study; BWHS, the Black Women’s Health Study; BMI, body mass index; CARDIA, Coronary Artery Risk Development in Young Adults Study; CSM, Cohort of Swedish Men; EPIC-NL, European Prospective Investigation into Cancer and Nutrition–Netherlands; EPIC-Norfolk, European Prospective Investigation into Cancer and Nutrition-Norfolk study; FFQ, food frequency questionnaires; FMCHES, Finnish Mobile Clinic Health Examination Survey; FOS, Framingham Offspring Study; HPFS, Health Professional Follow-up Study; HWCS, Health Workers Cohort Study; JPHC, Japan Public Health Center-based Prospective Study; MESA: Multi-Ethnic Study of Atherosclerosis; NHS: Nurses’ Health Study; NOMAS, Northern Manhattan Study; SCHS: Singapore Chinese Health Study; SSB, sugar-sweetened beverage; TCS, Thai Cohort Study; WHS: Women’s Health Study; WHI OS, Women’s Health Initiative observational study; WHI, Women’s Health Initiative.

Biomarkers included any of fasting glucose, two-hour glucose by oral glucose tolerance test, and glycated hemoglobin. Records included medical records or other records from registry, not including self-reported information.

^*^ Study quality was assessed with the Newcastle-Ottawa Scale.

**Supplementary Table 4. Summary of prospective studies on beverage and hypertension (n=10)**

| **Author, publication year, country** | **Characteristics of the study** | | | **Characteristics of the participant** | | | **Characteristics of the exposure** | | | **Characteristics of the outcome** | | | **Adjustment for confounding factors** | **Study quality^*^** |
| --- | --- | --- | --- | --- | --- | --- | --- | --- | --- | --- | --- | --- | --- | --- |
|  | **Study name** | **Baseline survey year** | **Follow up (year)** | **No.** | **Age range (year)** | **Men (%)** | **Assay method** | **Exposure** | **Definition** | **Ascertainment method** | **Definition** | **Cases (n)** |  |  |
| Ravi Dhingra et al, 2007, USA | FHS | 1987-91 | 4.0  (mean) | 64,499 | 52.9  (mean) | 42.5 | FFQ | SSB | Regular soft drinks | Self-report | Blood pressure ≥130/85 mmHg or antihypertensive treatment | 1,377 | Age, sex, smoking, physical activity, dietary energy intake, saturated fat, trans fat, fiber and magnesium intake, glycemic index | High |
| Jennifer A Nettleton et al, 2009, USA | MESA | 2000-02 | 5.8  (median) | 3,363 | 45-84 | 47.4 | FFQ | ASB | Diet soft drinks, unsweetened mineral water | Measure | Blood pressure ≥130/85 mmHg or antihypertensive treatment | 843 | Age, sex, race, smoking, education, BMI, waist circumference, physical activity, dietary energy intake | High |
| Kiyah J Duffey et al, 2010, USA | CARDIA | 1985-86 | 20.0 | 2,639 | 18-30 | 46.5 | FFQ | SSB | Sugar-sweetened soda and fruit drinks | Measure | Blood pressure ≥130/85 mmHg or antihypertensive treatment | 609 | Age, sex, race, CARDIA exam center, smoking, alcohol drinking, weight, physical activity, dietary energy intake, the 3 other beverages energy intake | Medium |
| Lisa Cohen et al, 2012, USA | NHS I | 1976-80 | 38.0 | 88,540 | 30-55 | 0.0 | FFQ | SSB, ASB | Sugar-sweetened cola, sugar-sweetened caffeine-free cola, sugar-sweetened non-cola, and fruit punch or other  sugar-sweetened fruit drink with naturally sugar or artificial sugar. | Self-report | Medical record review confirmed a documented systolic and diastolic BP>140 and 90 mmHg, | 42,022 | Age, race, smoking, alcohol drinking, BMI, weight change between surveys, physical activity, oral contraceptive use, non-narcotic analgesic use, family history of HTN, dietary energy, calcium, magnesium, vitamin D, cereal fiber, trans-fat, carbohydrate, and fructose intake, SSB (for ASB), ASB (for SSB), DASH-style diet | High |
| Lisa Cohen et al, 2012, USA | NHS II | 1989-91 | 16.0 | 97,991 | 25-42 | 0.0 | FFQ | SSB, ASB | Sugar-sweetened cola, sugar-sweetened caffeine-free cola, sugar-sweetened non-cola, and fruit punch or other  sugar-sweetened fruit drink with naturally sugar or artificial sugar. | Self-report | Medical record review confirmed a documented systolic and diastolic BP>140 and 90 mmHg, | 21,873 | Age, race, smoking, alcohol drinking, BMI, weight change between surveys, physical activity, oral contraceptive use, non-narcotic analgesic use, family history of HTN, dietary energy intake, calcium, magnesium, vitamin D, cereal fiber, trans-fat, carbohydrate and fructose intake, SSB (for ASB), ASB (for SSB), DASH-style diet | High |
| Lisa Cohen et al, 2012, USA | HPFS | 1986 | 22.0 | 37,360 | 40-75 | 100.0 | FFQ | SSB, ASB | Sugar-sweetened cola, sugar-sweetened caffeine-free cola, sugar-sweetened non-cola, and fruit punch or other  sugar-sweetened fruit drink with naturally sugar or artificial sugar. | Self-report | Medical record review confirmed a documented systolic and diastolic BP>140 and 90 mmHg, | 13,439 | Age, race, smoking, alcohol drinking, BMI, weight change between surveys, physical activity, non-  narcotic analgesic use, family history of HTN, dietary energy intake, calcium, magnesium, vitamin D, cereal fiber, trans-fat, carbohydrate and fructose intake, SSB (for ASB), ASB (for SSB), DASH-style diet | High |
| Carmen Sayon-Orea et al, 2015, Spain | SUN | 1999-  2010 | 8.1  (median) | 13,843 | 36.4  (mean) | 37.8 | FFQ | SSB. | Sugar sweetened carbonated colas and fruit-flavored carbonated sugar soft drinks | Self-report | Medical diagnosis of hypertension, a systolic blood pressure 140 mmHg, a diastolic blood pressure 90 mmHg, or any use of antihypertensive medication | 1,308 | Age, sex, smoking, alcohol drinking, education, BMI, physical activity, hypercholesterolemia, family history of hypertension, dietary energy intake, sodium, potassium, low fat dairy, olive oil, fruit, vegetables, cereals, legumes, meat, whole fat dairy and fish intake | High |
| Brandon J Auerbach et al, 2017, USA  (postmenopausal women) | WHI | 1993-98 | 7.8  (mean) | 80,539 | 50-79 | 0.0 | FFQ | Fruit juice | 100% orange and grapefruit juices, and all other 100% fruit juice types | Self-report | self-reported incident hypertension, medication to treat hypertension | 46,202 | Age, race, smoking, education, BMI, physical activity, hormone replacement therapy status, study arm and dietary energy intake | High |
| Jung Hyun Kwak et al, 2018, Korea | KoGES | 2001-02 | 8.0  (mean) | 5,775 | 40-69 | 45.6 | FFQ | SSB | soft drinks (coke or sprite) and other sweetened drinks (sweetened rice drink and sweetened citrus tea) | Measure | taking blood pressure medicines, or with measured systolic or diastolic blood pressures greater than 140 or 90 mmHg | 1,175 | Age, sex, smoking, alcohol drinking, education, income, physical activity, dietary energy intake, whole grains, dairy, fish, sodium and potassium intake | High |
| J. H. Siqueira et al, 2022, Brazil | ELSA | 2008-2010 | 4 | 6,124 | 50  (mean) | 40.9 | FFQ | SSB | Sugar-sweetened carbonated beverages | Measure | Systolic blood pressure ≥ 130 mmHg and/or diastolic blood pressure ≥ 85 mmHg or drug treatment (antihypertensive drugs) | 1,267 | Age, sex, race, smoking, alcohol drinking, income, physical activity, waist circumference, food groups (fruits, vegetables, processed meats, unprocessed meats, fish and seafood, milk and dairy products, and whole grains), and calorie intake | Medium |

ASB, artificially sweetened beverage; BMI, body mass index; CARDIA, Coronary Artery Risk Development in Young Adults; DASH, Dietary Approaches to Stop Hypertension; ELSA, Brazilian Longitudinal Adult Health Study; FFQ, food frequency questionnaire; FHS, Framingham Heart Study; HPFS, Health Professionals Follow-Up Study; HTN, hypertension; KoGES, Korean Genome and Epidemiologic Study; MESA, Multi-Ethnic Study of Atherosclerosis; NHS I, Nurses’ Health Study; NHS II, Nurses’ Health Study II; SSB, sugar-sweetened beverage; SUN, Seguimiento Universidad de Navarra; WHI, Women’s Health Initiative.

^*^ Study quality was assessed with the Newcastle-Ottawa Scale.

**Supplementary Table 5. Summary of prospective studies on beverage and coronary heart disease (n=8)**

| **Author,**  **publication year,**  **country** | **Characteristics of the study** | | | **Characteristics of the participant** | | | **Characteristics of the exposure** | | | **Characteristics of the outcome** | | | **Adjustment for confounding factors** | **Study quality^*^** |
| --- | --- | --- | --- | --- | --- | --- | --- | --- | --- | --- | --- | --- | --- | --- |
|  | **Study name** | **Baseline survey year** | **Follow up (year)** | **No.** | **Age range (year)** | **Men (%)** | **Assay method** | **Exposure** | **Definition** | **Ascertainment method** | **Definition** | **Cases (n)** |  |  |
| Teresa T Fung et al, 2009, USA | NHS | 1980 | 24.0 | 88,520 | 30-55 | 0.0 | FFQ | SSB, ASB | SSB: caffeinated and noncaffeinated colas [eg, Coke (Coca-Cola, Atlanta, GA), Pepsi (Persico Inc, Purchase, NY), and other colas with sugar], other carbonated beverages with sugar[eg,7-Up(DrPepperSnappleGroup,Plano, TX)], noncarbonated sweetened beverages [ie, Hawaiian  Punch (Dr Pepper Snapple Group), lemonade, and other noncarbonated fruit drinks].  ASB: artificially sweetened  beverages consisted of all types of low-calorie sweet carbonated  beverages, such as diet colas and other diet carbonated beverages | Records | CHD includes nonfatal  myocardial infarction (MI) or fatal CHD  MI was classified as confirmed if the criteria of the World Health Organization were met,specifically,symptoms and either electrocardiograph-detected changes or elevated cardiac enzyme concentrations  Fatal CHD was confirmed by hospital records or through an autopsy or if CHD was listed as the cause of death on the death certificate | 3,105 | Age, smoking, alcohol drinking, physical activity, history of hypertension and high blood cholesterol, family history of myocardial infarction before age 60 y, aspirin use, menopausal status and postmenopausal hormone use, and the Alternate Healthy Eating Index | High |
| Lawrence de Koning et al, 2012, USA | HPFS | 1986 | 22.0 | 42,883 | 40-75 | 100.0 | FFQ | SSB, ASB | SSB: fruit punches, lemonades or other fruit drinks  ASB: caffeinated low-calorie beverages and non-carbonated low-calorie beverage | Records | Incident CHD was defined as non-fatal or fatal myocardial infarction WHO criteria, which required clinical symptoms and diagnostic changes on electrocardiogram or elevated cardiac enzymes. Death ascertainment was performed by searching the National Death Index | 3,683 | Age, smoking, alcohol drinking, BMI, weight change, physical activity, history of T2D, high triglycerides, high cholesterol and high blood pressure, family history of CHD, dietary energy intake, diet quality (aHEI), and multivitamin use | High |
| Hannah Gardener et al, 2012, USA | NOMAS | 1993-01 | 9.8  (mean) | 2,564 | 68.6 | 36.0 | FFQ | SSB, ASB | SSB: regular soft drinks  ASB: regular soft drinks | Records | myocardial infarction (MI): No report | 155 | Age, sex, race/ethnicity, smoking, alcohol drinking, education, BMI, physical activity, peripheral vascular disease, diabetes, cardiac disease, hypertension, hypercholesterolemia, metabolic syndrome, dietary energy intake, protein, carbohydrates, total fat, saturated fat and sodium intake | High |
| Ehab S Eshak et al, 2012, Japan | JPHC I | 1990 | 18.0 | 39,786 | 40-59 | 47.4 | FFQ | SSB | Beverages that contained added caloric sweeteners  such as sucrose or high-fructose corn syrup; these soft drinks  included cola-type beverages, flavored juices, and non-100%  fruit juices | Records | No report | 453 | Age, smoking, alcohol drinking, job, BMI, physical activity, hypertension, diabetes, dietary energy intake, seafood, meat, fruit and sodium intake | High |
| K. Warfa et al, 2016, Sweden | MDC | 1991-96 | 17.0  (mean) | 26,190 | 44-73 | 38.0 | 7-day diet record | SSB | No report | Records | Fatal or non-fatal MI (International Classification of Diseases (ICD)-9 codes:  410A-410X; ICD-10: I21) or death attributable to ischaemic heart disease (ICD-9 codes: 410–414; ICD-10: I20-I25). | 2,493 | Age, sex, smoking, alcohol drinking, education, data collection method and season, physical activity, waist circumference, dietary energy intake, fruits, vegetables, wholegrains, coffee, fermented milk, meat and fish intake | High |
| Floor R. Scheffers et al, 2019, Netherlands | EPIC-NL | 1993-1997 | 14.6 (mean) | 34,560 | 48.8  (mean) | 26.0 | FFQ | Fruit juice | 100 % fruit juice without added sugars or artificial sweeteners and other components as artificial colours or preservatives. | Records | No report | 2,135 | Age, sex, smoking, alcohol drinking, education, BMI, physical activity, waist circumference, systolic blood pressure, energy intake, DHD15-index (indicator for dietary habits), fruit consumption, and total choleste | High |
| Amélie Keller et al, 2019, USA | HPP | 1980-1993 | 8.2 (median) | 284,345 | 54.3  (mean) | 23.9 | FFQ | SSB | Carbonated/non-carbonated and caffeinated/non-caffeinated sodas, sport drinks and fruit drinks with any type of added sugar. | Records | No report | 4,248 | Age, smoking, alcohol drinking, education, BMI, physical activity, hypertension, high cholesterol, diet, quintiles of cereal fibers; quintiles of trans-fat; quintiles of poly-unsaturated fat/saturated fat ratio, total energy, and the calendar year | Medium |
| Lorena S. Pacheco et al, 2020, USA | CTS | 1995-96 | 20.0 | 106,178 | 52.1 | 0.0 | FFQ | SSB | caloric soft drinks, sweetened bottled waters or teas, and fruit drinks | Records. | MI and CVD definitions followed the International Statistical Classification  of Diseases, Ninth Revision (ICD-9), and International  Statistical Classification of Diseases, Tenth Revision  (ICD-10), coding system | 2,677 | Age, race, smoking, alcohol drinking, income, BMI, physical activity, aspirin use, menopausal status and menopausal hormone therapy use, oral contraceptive use, history of hypertension, family history of cardiovascular disease, dietary energy intake, fruit and vegetable intake, and multivitamin use | High |

ASB, artificially sweetened beverage; aHEI, alternative healthy eating index; BMI, body mass index, CHD, coronary heart disease; CTS, California Teachers Study; EPIC-NL, European Prospective Investigation into Cancer and Nutrition–Netherlands; FFQ, food frequency questionnaire; HPFS, Health Professionals Follow-Up Study; HPP, Harvard Pooling Project; JPHC I, Japan Public Health Centre–based study cohort I; MDC, Malmö Diet and Cancer Cohort; MI, myocardial infarction; NHS, Nurses’ Health Study; NOMAS, Northern Manhattan Study; SSB, sugar-sweetened beverage.

^*^ Study quality was assessed with the Newcastle-Ottawa Scale.

**Supplementary Table 6. Summary of prospective studies on beverage and stroke (n=9)**

| **Author,**  **publication year,**  **country** | **Characteristics of the study** | | | **Characteristics of the participant** | | | **Characteristics of the exposure** | | | **Characteristics of the outcome** | | | **Adjustment for confounding factors** | **Study quality^*^** |
| --- | --- | --- | --- | --- | --- | --- | --- | --- | --- | --- | --- | --- | --- | --- |
|  | **Study name** | **Baseline survey year** | **Follow up (year)** | **No.** | **Age range (year)** | **Men (%)** | **Assay method** | **Exposure** | **Definition** | **Ascertainment method** | **Definition** | **Cases (n)** |  |  |
| Kaumudi J. Joshipura et al, 1999, USA | Combination of NHS and HPFS | NHS: 1980  HPFS:1986 | NHS: 14  HPFS:8 | 114,279 | NHS: 34-59  HPFS:40-75 | 33.8 | FFQ | Fruit juice | Citrus fruit juice | Records | No report | 570 | Age, smoking, alcohol drinking, BMI, physical activity, hypertension, hypercholesterolemia, family history of myocardial infarction, time period, aspirin use, postmenopausal hormone use, dietary energy intake, multivitamin supplement use, vitamin E use | High |
| Hannah Gardener et al, 2012, USA | NOMAS | 1993-01 | 9.8  (mean) | 2,564 | 69.0  (mean) | 36.0 | FFQ | SSB, ASB | SSB: regular soft drinks  ASB: diet soft drinks | Records | No report | 225 | Age, sex, race, smoking, alcohol drinking, education, BMI, physical activity, peripheral vascular disease, diabetes, cardiac disease, hypertension, hypercholesterolemia, metabolic syndrome, dietary energy intake, protein, carbohydrates, total fat, saturated fat and sodium intake | High |
| Ehab S Eshak et al, 2012, Japan | JPHC I | 1990 | 18.0 | 39,786 | 40-59 | 47.4 | FFQ | SSB. | Beverages that contained added caloric sweeteners such as sucrose or high-fructose corn syrup; these soft drinks included cola-type beverages, flavored juices, and non-100% fruit juices | Records | Stroke was confirmed if the criteria of the National Survey of Stroke (20) were met，, specifically if the presence offocal neurological deficits of sudden or rapid onset >24h,or until death | 1,922 | Age, smoking, alcohol drinking, job, BMI, physical activity, history of hypertension, diabetes, dietary energy intake, seafood, meat, fruit and sodium intake | High |
| Adam M Bernstein et al, 2012, USA | NHS | 1980 | 28.0 | 84,085 | 30-55 | 0.0 | FFQ | SSB | Sugar-sweetened cola with caffeine (eg, Coke, Pepsi), sugar sweetened cola without caffeine (eg, caffeine-free Coke, caffeinefree Pepsi), and other carbonated beverages with sugar (eg, 7-Up, Mountain Dew, Surge, Dr Pepper). | Records | Stroke was classified as ischemic (thrombotic, embolic, or nonhemorrhagic), hemorrhagic (intraparenchymal hemorrhage or subarachnoid hemorrhage), or of  unknown type, as per criteria in the National Survey of Stroke | 2,938 | BMI, weight change, and dietary energy intake | High |
| Adam M Bernstein et al,2012, USA | HPFS | 1986 | 22.0 | 43,371 | 40-75 | 100.0 | FFQ | SSB | Sugar-sweetened cola with caffeine (eg, Coke, Pepsi), sugar sweetened cola without caffeine (eg, caffeine-free Coke, caffeinefree Pepsi), and other carbonated beverages with sugar (eg, 7-Up, Mountain Dew, Surge, Dr Pepper). | Records | Stroke was classified as ischemic (thrombotic, embolic, or nonhemorrhagic), hemorrhagic (intraparenchymal hemorrhage or subarachnoid hemorrhage), or of  unknown type, as per criteria in the National Survey of Stroke | 1,416 | BMI, weight change, and dietary energy intake | High |
| Matthew P. Pase et al, 2017, USA | FHS | 1998-01 | 10.0 | 2,888 | 62.0  (mean) | 45.0 | FFQ | SSB, ASB | SSB: high-sugar carbonated beverages such as cola  ASB: sugar-free carbonated beverages such as diet cola | Records | Stroke as the rapid onset of focal neurological symptoms of presumed vascular origin, lasting >24 hours or resulting in  death. | 97 | Age, sex, smoking, physical activity, waist to hip ratio, treatment of hypertension, history of prevalent cardiovascular disease, atrial fibrillation, left ventricular hypertrophy, diabetes mellitus and systolic blood pressure, dietary energy intake, total cholesterol, high-density lipoprotein cholesterol, the dietary guidelines adherence index | High |
| Floor R. Scheffers et al, 2019, Netherlands | EPIC-NL | 1993-1997 | 14.6 (mean) | 34560 | 48.8 | 26.0 | FFQ | Fruit juice | 100 % fruit juice without added sugars or artificial sweeteners and other components as artificial colours or preservatives. | Records | No report | 1135 | Age, sex, smoking, alcohol drinking, education, BMI, physical activity, waist circumference, systolic blood pressure, energy intake, DHD15-index (indicator for dietary habits), fruit consumption, and total choleste | High |
| Lorena S. Pacheco et al, 2020, USA | CTS | 1995-96 | 20.0 | 106,178 | 52.1  (mean) | 0.0 | FFQ | SSB | caloric soft drinks, sweetened bottled waters or teas, and fruit drinks | Records | Stroke and CVD definitions followed the International Statistical Classification of Diseases, Ninth Revision (ICD-9), and International Statistical Classification of Diseases, Tenth Revision (ICD-10), coding system | 5,258 | Age, race, smoking, alcohol drinking, income, BMI, physical activity, aspirin use, menopausal status and menopausal hormone therapy use, oral contraceptive use, history of hypertension, family history of cardiovascular disease, dietary energy intake, fruit and vegetable intake, and multivitamin use | High |
| Anna Johansson et al, 2022, Swedish | MDCS | 1991-96 | 21.5  (median) | 23,797 | 58  (mean) | 36.8 | FFQ | SSB | No report | Records | Swedish National Patient register and the Cause of Death Register | 1,937 | Age, sex, smoking, education, BMI, physical activity and stroke heredity score, dyslipidemia, hypertension | High |

ASB, artificially sweetened beverage; BMI, body mass index; CTS, California Teachers Study; FFQ, food frequency questionnaire; FHS, Framingham Heart Study; HPFS, Health Professionals Follow-Up Study; HPP, Harvard Pooling Project; JPHC I, Japan Public Health Centre–based study cohort I;MDCS, Malmö Diet and Cancer Study; NHS, Nurses’ Health Study; NOMAS, Northern Manhattan Study; SSB, sugar-sweetened beverage.

^*^ Study quality was assessed with the Newcastle-Ottawa Scale.

**Supplementary Table 7. Summary of prospective studies on beverage and all-cause mortality (n=15)**

| **Author,**  **publication year,**  **country** | **Characteristics of the study** | | | **Characteristics of the participant** | | | **Characteristics of the exposure** | | | **Characteristics of the outcome** | | | **Adjustment for confounding factors** | **Study quality^*^** |
| --- | --- | --- | --- | --- | --- | --- | --- | --- | --- | --- | --- | --- | --- | --- |
|  | **Study name** | **Baseline survey year** | **Follow up (year)** | **No.** | **Age range (year)** | **Men (%)** | **Assay method** | **Exposure** | **Definition** | **Ascertainment method** | **Definition** | **Cases (n)** |  |  |
| Annlia Paganini-Hill et al, 2007, USA | LWCS | 1981 | 23.0 | 13,624 | 74.0  (median) | 36.6 | FFQ | SSB, ASB | SSB: cola beverages with sugar, other soft drinks with sugar  ASB: cola beverages artificially sweetened, other soft drinks artificially sweetened | Death records, National Death Index, death certificates | The International Classification of Diseases, Ninth Revision (ICD-9) was used to define the diagnoses of interest: hip fracture, code 820; acute myocardial infarction, code 410; and cancer, codes 140-208, excluding 196-198 | 11,386 | Age, sex, smoking, alcohol drinking, BMI, physical activity, history of hypertension, angina, heart attack, stroke, diabetes, rheumatoid arthritis and cancer | High |
| Ankur Vyas et al, 2014,USA | WHI OS | 1993-98 | 8.7  (mean) | 59,614 | 62.8  (mean) | 0.0 | FFQ | SSB, ASB | SSB: regular soft drinks (not diet)fruit juice, and fruit drinks  ASB: diet drinks | National Death Index | No report | 4,437 | Age, race, smoking, alcohol drinking, education, income, BMI, physical activity, hormone therapy, history of diabetes, hypertension and hyperlipidemia, dietary energy inake, salt intake, and SSB (for ASB), ASB (for SSB) | High |
| Andrew O Odegaard et al, 2015, Singapore | SCHS | 1993-98 | 16.3  (median) | 52,584 | 45-74 | 50.2 | FFQ | SSB | Soft drinks | Death records | International Classification of Diseases version 9 codes  Deaths from cardiovascular disease (CVD) (codes 394.0–459.0) | 10,029 | Age, sex, dialect, smoking, education, year of interview, BMI, physical activity, sleep, hypertension, dietary energy intake, nonbeverage vegetable-fruit-soy-rich dietary pattern score | High |
| Wendy E Barrington et al, 2016, USA | VITAL | 2000-02 | 6.9  (mean) | 69,582 | 50-76 | 48.8 | FFQ | SSB | Sugar-sweetened soda (not diet), fruit drinks not including  juice (e.g. Hi-C, Gatorade, lemonade) and cranberry juice | Death records, National Death Index, social security | Causes of death were  categorized as due to CVD (I00–I99) or cancer (C00–D48) | 4,187 | Age, sex, race, smoking, alcohol drinking, education, marital status, income, BMI, change in BMI, physical activity, morbidity score, self-rated health, cholesterol-lowering medication use, aspirin use and non-aspirin non-steroidal anti-inflammatory drug use in last 10 years, years of oestrogen therapy and oestrogen plus progestin therapy, age at menopause, death of father and death of mother, mammogram and prostrate-specific antigen test in the last 2 years, sigmoidoscopy in the last 10 years, dietary energy intake, fruits and vegetables intake | High |
| Vasanti S Malik et al, 2019, USA | NHS | 1980 | 34.0 | 80,647 | 30-55 | 0.0 | FFQ | SSB, ASB | SSB:caffeinated colas, caffeine-free colas, other  (ie, noncola) carbonated sugar-sweetened beverages  ASB: caffeinated caffeinefree, and noncarbonated low-calorie or diet beverages. | Death records  National Death Index, death certificates | Deaths caused by CVD (ICD codes 390–458 and cancer (ICD codes 140–207 ICD code 174 for breast cancer mortality, ICD code 162 for  lung cancer mortality, and ICD codes 153 and 154 for colon cancer mortality | 23,432 | Age, race, smoking, alcohol drinking, BMI, physical activity, postmenopausal hormone use, aspirin use, history of hypertension and hypercholesterolemia, family history of diabetes, myocardial infarction and cancer, dietary energy intake, whole grains, fruit, vegetables, red and processed meat and multivitamin intake | High |
| Vasanti S Malik et al, 2019, USA | HPFS | 1986 | 28.0 | 37,716 | 40-75 | 100.0 | FFQ | SSB, ASB | SSB:caffeinated colas, caffeine-free colas, other  (ie, noncola) carbonated sugar-sweetened beverages  ASB: caffeinated caffeinefree, and noncarbonated low-calorie or diet beverages | Death records  National Death Index, death certificates | Deaths caused by CVD (ICD codes 390–458 and cancer (ICD codes 140–207 ICD code 174 for breast cancer mortality, ICD code 162 for  lung cancer mortality, and ICD codes 153 and 154 for colon cancer mortality | 13,004 | Age, race, smoking, alcohol drinking, BMI, physical activity, postmenopausal hormone use, aspirin use, history of hypertension and hypercholesterolemia, family history of diabetes, myocardial infarction and cancer, dietary energy intake, whole grains, fruit, vegetables, red and processed meat and multivitamin intake | High |
| Amy Mullee et al, 2019, European | EPIC | 1992-  2000 | 16.4  (mean) | 451,743 | 50.8  (median) | 28.9 | FFQ | SSB, ASB | SSB: fizzy soft drinks, eg cola, lemonade, fruit squash or cordia  ASB: low calorie or diet fizzy soft drinks | Death records. | nternational Statistical Classification of Diseases and Related Health Problems, Tenth Revision (ICD-10) codes were used to classify the underlying cause of death  Cerebrovascular disease (ICD-10 codes I60-I69); ischemic heart disease (ICD-10 codes I20-I25) | 41,693 | Age, sex, EPIC center, smoking, alcohol drinking, education, BMI, physical activity, menopausal hormone and contraceptive pilluse use, menopausal status, dietary energy intake, red and processed meat, fruits, vegetables, coffee, fruit and vegetable juice intake | High |
| Lindsay J Collin et al, 2019, USA | REGARDS | 2003-07 | 6.0  (mean) | 13,440 | 63.6  (mean) | 59.3 | FFQ | SSB, Fruit juice | SSB: sodas, soft drinks, or fruit-flavored drinks  Fruit juice: naturally sweet 100% fruit juices | Death records, National Death Index, death certificates | No report | 1,000 | Age, sex, race, smoking, alcohol drinking, education, BMI, physical activity and diet | High |
| Stina Ramne et al, 2019, Swedish | MDCS | 1991-1996 | 20(mean) | 24,272 | 57.6 | 38.6 | Food diary,FFQ | SSB | No report | Death records | No report | 6,309 | Age, sex, somking, alcohol drinking, education, BMI, physical activity, energy intake (and season and screening date in the MDCS), and dietary habits (fruit and vegetables, processed meat, coffee, SFAs, and fiber density) | High |
| Stina Ramne et al, 2019, Swedish | NSHDS | 1991-1996 | 20(mean) | 23,375 | 48.6 | 46.3 | FFQ | SSB | No report | Death records | No report | 2,881 | Age, sex, somking, alcohol drinking, education, BMI, physical activity, energy intake (and season and screening date in the MDCS), and dietary habits (fruit and vegetables, processed meat, coffee, SFAs, and fiber density) | High |
| Jana J Anderson et al, 2020, UK | UKBS | 2007-10 | 7.0  (mean) | 161,415 | 40-69 | 44.0 | 24-h diet record | SSB, ASB | Sugar- and artificially sweetened beverages (e.g. squash, cordial or fizzy drinks) | Death certificates | Death certificates held by the National Health Service (NHS) Information Centre for England and Wales, and the NHS Central Register for Scotland. | 2,311 | Age, sex, race, smoking ,alcohol drinking, education, income, BMI, physical activity, sedentary behaviour, dietary energy intake, fresh fruit, vegetable, fibre, red meat and processed meat intake | High |
| Yan-Bo Zhang et al, 2021, USA | NHANES | 1999-  2014 | 7.9  (mean) | 31,402 | 46.7  (mean) | 50.5 | 24-h diet  record | SSB, ASB | SSB: Sodas, fruit-favored drinks, sugar-added fruit juices, sweetened cofees and teas, sport and energy drinks, and other sweetened drinks.  ASB: sugarfree soft drinks and carbonated water | National Death Index | Deaths from all causes, heart disease (codes I00-I09, I11, I13, and I20-I51) | 3,878 | Age, sex, race, smoking, alcohol drinking, education, marital status, income, BMI, physical activity, prevalent high cholesterol level, history of hypertension, diabetes, cardiovascular disease and cancer, dietary energy intake, SSB (for ASB), ASB (for SSB), healthy eating index score-2015 | High |
| Lorena S Pacheco et al, 2022, USA | CTS | 1995-96 | 20.0 | 100,314 | 22-84 | 0.0 | FFQ | SSB | Rregular soft drinks (not diet soda), Snapple, Calistoga, sweetened bottled waters or iced teas, and Kool-Aid, Hi-C, or other drinks with added vitamin C | Death records | Using the International Classification of Diseases (ICD) 9th18 and 10th19 Revision codes  CVD-specific mortality (ICD-9 codes 390-398, 402, 404, 410-429, and 430-438  and ICD-10 codes I00 to I09, I11, I13, and I20 to I51, I60 to I69) that includes diseases of the heart, hypertension, atherosclerosis, and cerebrovascular diseases | 14,143 | Age, race, smoking, alcohol drinking, marital status, income, BMI, physical activity, aspirin use, antihypertension medication use, menopausal hormone therapy use, oral contraceptive use, menopausal status, history of hypertension, dietary energy intake, fruit and vegetable, red meat, processed meat, fish, refined carbohydrates, dietary fiber, coffee/tea beverages and multivitamin intake | High |
| Zhuang Zhang  et al, 2022, USA | NHANES | 1999-2000 | 7.8  (median) | 40,074 | 47.3  (mean) | 46.8 | 24-h diet  record | fruit juice | 100% fruit juice | National Death Index | Deaths from all causes, heart disease (codes I00-I09, I11, I13, and I20-I51) | 4,904 | Age, sex, race, smoking, alcohol drinking, education, marital status, income, BMI, physical activity, diabetes, baseline of cancer, dyslipidemia, hypertension, other CVDs, total energy intake, and HEI-2015 | High |
| Novita D Naomi et al, 2022, UK | Lifelines Cohort Study | 2006-  2013 | 9.8  (mean) | 118,707 | 45  (mean) | 40.0 | FFQ | SSB, ASB | SSB: all soft drinks or lemonades with sugar, such as coke and orange-favored soft  drinks, or lemonade with syrup  ASB: all diet soft drinks or lemonades where sugar was replaced by low/non-calorie sweeteners. | National Personal Records | No report | 2,852 | Age, sex, smoking, alcohol drinking, education, BMI, physical activity, sedentary behavior, energy intake, consumptions of grain, potatoes, vegetables, fruit, meat and processed meat, cofee, tea, legumes, nuts, fats and oils, sugary foods, mutual adjustment for other beverages (SSB, LNCB, and  fruit juice) | High |

ASB, artificially sweetened beverage; BMI, body mass index; CTS, California Teachers Study; EPIC, European Prospective Investigation into Cancer and Nutrition; FFQ, food frequency questionnaire; HPFS, Health Professional’s Follow-up study; LWCS, Leisure World Cohort Study; MDCS, Malmö Diet and Cancer Study; NHANES, National Health and Nutrition Examination Survey; NHS, Nurses’ Health study; NSHDS, Northern Swedish Health and Disease Study; REGARDS, Reasons for Geographic and Racial Differences in Stroke; SCHS, Singapore Chinese Health Study; SSB, sugar-sweetened beverage; UKBS, UK Biobank study; VITAL, Vitamins and Lifestyle; WHIOS, Women’s Health Initiative Observational Study.

^*^ Study quality was assessed with the Newcastle-Ottawa Scale.

**Supplementary Table 8. Summary of prospective studies on beverage and cardiovascular disease mortality (n=10)**

| **Author,**  **publication year,**  **country** | **Characteristics of the study** | | | **Characteristics of the participant** | | | **Characteristics of the exposure** | | | **Characteristics of the outcome** | | | **Adjustment for confounding factors** | **Study quality^*^** |
| --- | --- | --- | --- | --- | --- | --- | --- | --- | --- | --- | --- | --- | --- | --- |
|  | **Study name** | **Baseline survey year** | **Follow up (year)** | **No.** | **Age range (year)** | **Men (%)** | **Assay method** | **Exposure** | **Definition** | **Ascertainment method** | **Definition** | **Cases (n)** |  |  |
| Ankur Vyas et al, 2014, USA | WHI OS | 1993-98 | 8.7  (mean) | 59,614 | 62.8  (mean) | 0.0 | FFQ | SSB, ASB | SSB: regular soft drinks (not diet)fruit juice, and fruit drinks  ASB: diet drinks | National Death Index | No report | 942 | Age, race, smoking, alcohol drinking, education, income, BMI, physical activity, hormone therapy, history of diabetes, hypertension and hyperlipidemia, dietary energy intake, salt intake, SSB (for ASB) and ASB (for SSB) | High |
| Andrew O Odegaard et al, 2015, Singapore | SCHS | 1993-98 | 16.3  (median) | 52,584 | 45-74 | 50.2 | FFQ | SSB | Soft drinks | Records | International Classification of Diseases version 9 codes  Deaths from cardiovascular disease (CVD) (codes 394.0–459.0) | 3,097 | Age, sex, dialect, year of interview, smoking, education, BMI, physical activity, sleep, history of hypertension, dietary energy intake, and non-beverage vegetable-fruit-soy-rich dietary pattern score | High |
| Wendy E Barrington et al, 2016, USA | VITAL | 2000-02 | 6.9  (mean) | 69,582 | 50-76 | 48.8 | FFQ | SSB | Sugar-sweetened soda (not diet), fruit drinks not including  juice (e.g. Hi-C, Gatorade, lemonade) and cranberry juice | Death records, National Death Index, social security | Causes of death were  categorized as due to CVD (I00–I99) or cancer (C00–D48) | 1,066 | Age, sex, race, smoking, alcohol drinking, education, marital status, income, BMI, change in BMI, physical activity, morbidity score, self-rated health, cholesterol-  lowering medication use, aspirin use and non-aspirin non-steroidal anti-inflammatory drug use in last 10 years, years of oestrogen therapy and oestrogen plus progestin therapy, age at menopause, death of father and death of mother, mammogram and prostrate-specific antigen test in the last 2 years, sigmoidoscopy in the last 10 years, dietary energy intake, fruits and vegetables intake | High |
| Vasanti S. Malik et al, 2019, USA | NHS | 1980 | 34.0 | 80,647 | 30-55 | 0.0 | FFQ | SSB, ASB | SSB:caffeinated colas, caffeine-free colas, other  (ie, noncola) carbonated sugar-sweetened beverages  ASB: caffeinated caffeinefree, and noncarbonated low-calorie or diet beverages. | Death records  National Death Index, death certificates | Deaths caused by CVD (ICD codes 390–458 and cancer (ICD codes 140–207 ICD code 174 for breast cancer mortality, ICD code 162 for  lung cancer mortality, and ICD codes 153 and 154 for colon cancer mortality | 4,139 | Age, race, smoking, alcohol drinking, BMI, physical activity, postmenopausal hormone use, aspirin use, multivitamin use, history of hypertension and hypercholesterolemia, family history of diabetes, myocardial infarction and cancer, dietary energy intake, whole grains, fruit, vegetables, red and processed meat intake | High |
| Vasanti S. Malik et al, 2019, USA | HPFS | 1986 | 28.0 | 37,716 | 40-75 | 100.0 | FFQ | SSB, ASB | SSB:caffeinated colas, caffeine-free colas, other  (ie, noncola) carbonated sugar-sweetened beverages  ASB: caffeinated caffeinefree, and noncarbonated low-calorie or diet beverages | Death records  National Death Index, death certificates | Deaths caused by CVD (ICD codes 390–458 and cancer (ICD codes 140–207 ICD code 174 for breast cancer mortality, ICD code 162 for  lung cancer mortality, and ICD codes 153 and 154 for colon cancer mortality | 3,757 | Age, race, smoking, alcohol drinking, BMI, physical activity, postmenopausal hormone use,aspirin use, history of hypertension and hypercholesterolemia, family history of myocardial infarction and cancer, dietary energy intake, whole grains, fruit, vegetables, red and processed meat intake, multivitamin use | High |
| Lindsay J Collin et al, 2019, USA | REGARDS | 2003-07 | 6.0  (mean) | 13,440 | 63.6  (mean) | 59.3 | FFQ | SSB, fruit juice | SSB: sodas, soft drinks, or fruit-flavored drinks  Fruit juice: naturally sweet 100% fruit juices | Death records, National Death Index, death certificates | No report | 168 | Age, sex, race, smoking, alcohol drinking, education, BMI, physical activity and diet | High |
| Amélie Keller et al, 2019, USA | HPP | 1980-1993 | 8.2 (median) | 284,345 | 54.3(mean) | 23.9 | FFQ | SSB | Carbonated/non-carbonated and caffeinated/non-caffeinated sodas, sport drinks and fruit drinks with any type of added sugar. | Death records, autopsy reports, death certificates | No report | 1,630 | Age, smoking, alcohol drinking, education, BMI, physical activity, hypertension, high cholesterol, diet, quintiles of cereal fibers; quintiles of trans-fat; quintiles of poly-unsaturated fat/saturated fat ratio, total energy, and the calendar year | Medium |
| Yan-Bo Zhang et al, 2021, USA | NHANES | 1999-14 | 7.9  (mean) | 31,402 | >20 | 50.45 | 1. h diet record | SSB, ASB | SSB: Sodas, fruit-favored drinks, sugar-added fruit juices, sweetened cofees and teas, sport and energy drinks, and other sweetened drinks.  ASB: sugarfree soft drinks and carbonated water | National Death Index | Deaths from all causes, heart disease (codes I00-I09, I11, I13, and I20-I51) | 676 | Age, sex, race, smoking, alcohol drinking, education, marital status, income, BMI, physical activity, prevalent high cholesterol level, history of hypertension, diabetes, cardiovascular disease and cancer, dietary energy intake, healthy eating index score-2015,SSB (for ASB) and ASB (for SSB) | High |
| Zhuang Zhang  et al, 2022, USA | NHANES | 1999-2000 | 7.8  (median) | 40,074 | 47.3  (mean) | 46.8 | 24-h diet  record | fruit juice | 100% fruit juice | National Death Index | Deaths from all causes, heart disease (codes I00-I09, I11, I13, and I20-I51) | 1,029 | Age, sex, race, smoking, alcohol drinking, education, marital status, income, BMI, physical activity, diabetes, baseline of cancer, dyslipidemia, hypertension, other CVDs, total energy intake, and HEI-2015 | High |
| Lorena Sonia Pacheco et al, 2022, USA | CTS | 1995-96 | 20.0 | 100,314 | 22-84 | 0.0 | FFQ | SSB | Regular soft drinks (not diet soda), Snapple, Calistoga, sweetened bottled waters or iced teas, and Kool-Aid, Hi-C, or other drinks with added vitamin C | Death records | Using the International Classification of Diseases (ICD) 9th18 and 10th19 Revision codes  CVD-specific mortality (ICD-9 codes 390-398, 402, 404, 410-429, and 430-438  and ICD-10 codes I00 to I09, I11, I13, and I20 to I51, I60 to I69) that includes diseases of the heart, hypertension, atherosclerosis, and cerebrovascular diseases | 4,313 | Age, race, smoking, alcohol drinking, marital status, income, BMI, physical activity, aspirin use, menopausal status, multivitamin use, antihypertension medication use, menopausal hormone therapy use, oral contraceptive use, history of hypertension, dietary energy intake, fruit and vegetable, red meat, processed meat, fish, refined carbohydrates, dietary fiber and coffee/tea beverages intake | High |

ASB, artificially sweetened beverage; BMI, body mass index; CTS, California Teachers Study; HPFS, Health Professional’s Follow-up study; HPP, Harvard Pooling Project; NHANES, National Health and Nutrition Examination Survey; NHS, Nurses’ Health study; SCHS, Singapore Chinese Health Study; REGARDS, Reasons for Geographic and Racial Differences in Stroke; SSB, sugar-sweetened beverage; VITAL, Vitamins and Lifestyle; WHI OS, Women’s Health Initiative Observational Study; NR, not report.

^*^ Study quality was assessed with the Newcastle-Ottawa Scale.

**Supplementary Table 9. Newcastle Ottawa Scale assessments for prospective studies on beverage and type 2 diabetes**

| **First author, year** | **Study Selection** | **Comparability** | **Ascertainment of outcome** | **Total Score** | **Quality^*^** |
| --- | --- | --- | --- | --- | --- |
| Pereira et al, 2005 | 3 | 2 | 2 | 7 | High |
| Paynter et al, 2006 | 4 | 2 | 3 | 9 | High |
| Montonen et al, 2007 | 4 | 2 | 3 | 9 | High |
| Palmer et al, 2008 | 3 | 2 | 2 | 7 | High |
| Nettleton et al, 2009 | 4 | 2 | 3 | 9 | High |
| Odegaard et al, 2010 | 3 | 2 | 3 | 8 | High |
| Koning et al, 2011 | 3 | 2 | 2 | 7 | High |
| Pan et al, 2012 | 3 | 2 | 2 | 7 | High |
| Bhupathiraju et al, 2012 | 3 | 1 | 2 | 6 | Medium |
| Fagherazzi et al, 2013 | 3 | 1 | 2 | 6 | Medium |
| Eshak et al, 2013 | 4 | 2 | 2 | 8 | High |
| Sakurai et al, 2014 | 3 | 2 | 3 | 8 | High |
| Connor et al, 2015 | 3 | 2 | 1 | 6 | Medium |
| Huang et al, 2017 | 3 | 2 | 2 | 7 | High |
| Auerbach et al, 2017 | 3 | 2 | 1 | 6 | Medium |
| Papier et al, 2017 | 3 | 2 | 1 | 6 | Medium |
| Gardener et al, 2018 | 4 | 2 | 2 | 8 | High |
| Hirahatake et al, 2019 | 4 | 2 | 2 | 8 | High |
| Scheffers et al, 2020 | 3 | 1 | 2 | 6 | Medium |
| Torres-Ibarra et al, 2020 | 3 | 2 | 2 | 7 | High |

Study quality was assessed based on the nine-star Newcastle–Ottawa Scale (NOS) using pre-defined criteria namely: selection (population representativeness), comparability (adjustment for confounders), and ascertainment of outcome. The NOS assigns a maximum of four points for selection, two points for comparability, and three points for outcome. Nine points on the NOS reflects the highest study quality.

**Supplementary Table 10. Newcastle Ottawa Scale assessments for prospective studies on beverage and hypertension**

| **First author, year** | **Study Selection** | **Comparability** | **Ascertainment of outcome** | **Total Score** | **Quality*** |
| --- | --- | --- | --- | --- | --- |
| Dhingra et al, 2007 | 4 | 2 | 2 | 8 | High |
| Nettleton et al, 2009 | 4 | 2 | 3 | 9 | High |
| Duffey et al, 2010 | 3 | 2 | 1 | 6 | Medium |
| Cohen et al, 2012 (NHS I) | 3 | 2 | 2 | 7 | High |
| Cohen et al, 2012 (NHS II) | 3 | 2 | 2 | 7 | High |
| Cohen et al, 2012 (HPFS) | 3 | 2 | 2 | 7 | High |
| Sayon-Orea et al, 2014 | 3 | 2 | 2 | 7 | High |
| Auerbach et al, 2017 | 4 | 2 | 2 | 8 | High |
| Kwak et al, 2018 | 3 | 2 | 3 | 8 | High |
| Siqueira et al, 2022 | 1 | 2 | 2 | 5 | Medium |

Study quality was assessed based on the nine-star Newcastle–Ottawa Scale (NOS) using pre-defined criteria namely: selection (population representativeness), comparability (adjustment for confounders), and ascertainment of outcome. The NOS assigns a maximum of four points for selection, two points for comparability, and three points for outcome. Nine points on the NOS reflects the highest study quality.

HPFS, Health Professionals Follow-Up Study; NHS I, Nurses’ Health Study; NHS II, Nurses’ Health Study II.

**Supplementary Table 11. Newcastle Ottawa Scale assessments for prospective studies on beverage and coronary heart disease**

| **First author, year** | **Study Selection** | **Comparability** | **Ascertainment of outcome** | **Total Score** | **Quality*** |
| --- | --- | --- | --- | --- | --- |
| Fung et al, 2009 | 3 | 2 | 3 | 8 | High |
| Koning et al, 2012 | 3 | 2 | 3 | 8 | High |
| Gardener et al, 2012 | 3 | 2 | 2 | 7 | High |
| Eshak et al, 2012 | 3 | 2 | 3 | 8 | High |
| Warfa et al, 2016 | 3 | 2 | 3 | 8 | High |
| Scheffers et al, 2019 | 3 | 2 | 2 | 7 | High |
| Keller et al, 2019 | 1 | 2 | 2 | 5 | Medium |
| Pacheco et al, 2020 | 3 | 2 | 2 | 7 | High |

Study quality was assessed based on the nine-star Newcastle–Ottawa Scale (NOS) using pre-defined criteria namely: selection (population representativeness), comparability (adjustment for confounders), and ascertainment of outcome. The NOS assigns a maximum of four points for selection, two points for comparability, and three points for outcome. Nine points on the NOS reflects the highest study quality.

**Supplementary Table 12. Newcastle Ottawa Scale assessments for prospective studies on beverage and stroke**

| **First author, year** | **Study Selection** | **Comparability** | **Ascertainment of outcome** | **Total Score** | **Quality*** |
| --- | --- | --- | --- | --- | --- |
| Joshipura et al, 1999 | 3 | 2 | 3 | 8 | High |
| Gardener et al, 2012 | 3 | 2 | 2 | 7 | High |
| Eshak et al, 2012 | 3 | 2 | 3 | 8 | High |
| Bernstein et al, 2012 (NHS) | 3 | 2 | 3 | 8 | High |
| Bernstein et al, 2012 (HPFS) | 3 | 2 | 3 | 8 | High |
| Pase et al, 2017 | 3 | 2 | 3 | 8 | High |
| Keller et al, 2019 | 1 | 2 | 2 | 5 | Medium |
| Pacheco et al, 2020 | 3 | 2 | 2 | 7 | High |
| Johansson et al, 2022 | 3 | 2 | 3 | 8 | High |

Study quality was assessed based on the nine-star Newcastle–Ottawa Scale (NOS) using pre-defined criteria namely: selection (population representativeness), comparability (adjustment for confounders), and ascertainment of outcome. The NOS assigns a maximum of four points for selection, two points for comparability, and three points for outcome. Nine points on the NOS reflects the highest study quality.

CSM, Cohort of Swedish Men; HPFS, Health Professionals Follow-Up Study; NHS, Nurses’ Health Study; SMC, Swedish Mammography Cohort.

**Supplementary Table 13. Newcastle Ottawa Scale assessments for prospective studies on beverage and all-cause mortality**

| **First author, year** | **Study Selection** | **Comparability** | **Ascertainment of outcome** | **Total Score** | **Quality*** |
| --- | --- | --- | --- | --- | --- |
| Paganini-Hill et al, 2007 | 2 | 2 | 3 | 7 | High |
| Vyas et al, 2014 | 3 | 2 | 2 | 7 | High |
| Odegaard et al, 2015 | 3 | 2 | 3 | 8 | High |
| Barrington1 et al,2016 | 3 | 2 | 3 | 8 | High |
| Malik et al, 2019 (NHS) | 3 | 2 | 3 | 8 | High |
| Malik et al, 2019 (HPFS) | 3 | 2 | 3 | 8 | High |
| Mullee et al, 2019 | 3 | 2 | 2 | 7 | High |
| Collin et al, 2019 | 3 | 2 | 2 | 7 | High |
| Ramne et al, 2019 (MDCS) | 3 | 2 | 2 | 7 | High |
| Ramne et al, 2019 (NSHDS) | 3 | 2 | 2 | 7 | High |
| Anderson et al, 2020 | 4 | 2 | 2 | 8 | High |
| Zhang et al, 2021 | 4 | 2 | 2 | 8 | High |
| Pacheco et al, 2022 | 3 | 2 | 3 | 8 | High |
| Zhang et al, 2022 | 4 | 2 | 2 | 8 | High |
| Naomi et al, 2022, | 3 | 2 | 2 | 7 | High |

Study quality was assessed based on the nine-star Newcastle–Ottawa Scale (NOS) using pre-defined criteria namely: selection (population representativeness), comparability (adjustment for confounders), and ascertainment of outcome. The NOS assigns a maximum of four points for selection, two points for comparability, and three points for outcome. Nine points on the NOS reflects the highest study quality.

HPFS, Health Professionals Follow-Up Study; NHS, Nurses’ Health Study.

**Supplementary Table 14. Newcastle Ottawa Scale assessments for prospective studies on beverage and cardiovascular disease mortality**

| **First author, year** | **Study Selection** | **Comparability** | **Ascertainment of outcome** | **Total Score** | **Quality*** |
| --- | --- | --- | --- | --- | --- |
| Vyas et al, 2014 | 3 | 2 | 2 | 7 | High |
| Odegaard et al, 2015 | 3 | 2 | 3 | 8 | High |
| Barrington et al, 2016 | 3 | 2 | 3 | 8 | High |
| Malik et al, 2019 (NHS) | 3 | 2 | 3 | 8 | High |
| Malik et al, 2019 (HPFS) | 3 | 2 | 3 | 8 | High |
| Collin et al, 2019 | 3 | 2 | 2 | 7 | High |
| Keller et al, 2019 | 1 | 2 | 2 | 5 | Medium |
| Zhang et al, 2021 | 4 | 2 | 2 | 8 | High |
| Zhang et al, 2022 | 4 | 2 | 2 | 8 | High |
| Pacheco et al, 2022 | 3 | 2 | 3 | 8 | High |

Study quality was assessed based on the nine-star Newcastle–Ottawa Scale (NOS) using pre-defined criteria namely: selection (population representativeness), comparability (adjustment for confounders), and ascertainment of outcome. The NOS assigns a maximum of four points for selection, two points for comparability, and three points for outcome. Nine points on the NOS reflects the highest study quality.

HPFS, Health Professionals Follow-Up Study; NHS, Nurses’ Health Study.

**Supplemental Table 15** Quality of meta-analysis for SSBs: the NutriGrade scoring system^1^

| Items | The association between SSB intakes | | | | | |
| --- | --- | --- | --- | --- | --- | --- |
|  | T2D | Hypertension | CHD | Stroke | all-cause mortality | cardiovascular disease mortality |
| Risk of bias/ study quality/ study limitations^2^ | Average NOS score=7.29 (2 P) | Average NOS score=6.88 (1 P) | Average NOS score=7.29 (2 P) | Average NOS score=7.71 (2 P) | Average NOS score=7.50 (2 P) | Average NOS score=7.44 (2 P) |
| Precision^3^ | 30,666 events; RR: 1.27; 95% CI: 1.17, 1.38 (1 P) | 82,418 events; RR: 1.12; 95% CI: 1.08, 1.17 (1 P) | 16,814 events; RR: 1.17; 95% CI: 1.09, 1.25 (1 P) | 13,793events; RR: 1.09; 95% CI: 1.00, 1.17 (1 P) | 121,488 events; RR: 1.11; 95% CI: 1.05, 1.16 (1 P) | 18,864 events; RR: 1.12; 95% CI: 1.06, 1.19 (1 P) |
| Heterogeneity^4^ | 17 cohorts; *I*^2^=69.2%, *P<*0.001; random-effects model with subgroup analysis (1 P) | 8 cohorts; *I*^2^=50.3%, *P*=0.05; random-effects model with subgroup analysis (0.5 P) | 7 cohorts; *I*^2^=0.0%, *P*=0.83; random-effects model with subgroup analysis (0.8 P) | 7 cohorts; *I*^2^=6.9%, *P*=0.38 random-effects model with subgroup analysis (0.8 P) | 14 cohorts; *I*^2^=74.8%, *P<*0.001; random-effects model with subgroup analysis (1 P) | 9 cohorts; *I*^2^=14.0%, *P*=0.32; random-effects model with subgroup analysis (0.8 P) |
| Directness^5^ | General populations; all studies investigated the association with dietary intakes of SSBs with T2D (1 P) | General populations; all studies investigated the association with dietary intakes of SSBs with hypertension (1 P) | General populations; all studies investigated the association with dietary intakes of SSBs with CHD (1 P) | General populations; all studies investigated the association with dietary intakes of SSBs with stroke (1 P) | General populations; all studies investigated the association with dietary intakes of SSBs with all-cause mortality (1 P) | General populations; all studies investigated the association with dietary intakes of SSBs with cardiovascular disease mortality (1 P) |
| Publication bias^6^ | 17 cohorts; no publication bias (1 P) | 8 cohorts; no publication bias (0.5 P) | 7 cohorts; no publication bias (0.5 P) | 7 cohorts; no publication bias (0.5 P) | 14 cohorts; no publication bias (1 P) | 9 cohorts; no publication bias (0.5 P) |
| Funding bias^7^ | Academic institutions (1 P) | Academic institutions (1 P) | Academic institutions (1 P) | Academic institutions (1 P) | Academic institutions (1 P) | Academic institutions (1 P) |
| Effect size^8^ | RR: 1.20; 95% CI: 1.12, 1.28 (1 P) | RR: 1.10; 95% CI: 1.08, 1.13 (0 P) | RR: 1.20; 95% CI: 1.12, 1.28 (1 P) | RR: 1.16; 95% CI: 1.08, 1.26 (1 P) | RR: 1.08; 95% CI: 1.06, 1.09 (0 P) | NC (0 P) |
| Dose-response^9^ | Linear dose-response relationship (1 P) | Linear dose-response relationship (1 P) | Linear dose-response relationship (1 P) | Linear dose-response relationship (1 P) | Linear dose-response relationship (1 P) | NO dose-response relationship (0 P) |
| Overall score | 9.0 P | 6.0 P | 8.3 P | 8.3 P | 8.0 P | 6.3 P |

^1^ CHD, coronary heart disease; NOS, Newcastle-Ottawa Scale; NC, not calculated; P, points; T2D, type 2 diabetes; SSB, sugar-sweetened beverage.

^2^NOS score (mean): ≥7 (2 P); 4-6.9 (1 P); 0-3.9 (0 P).

^3^<500 events OR ≥500 events but 95% CI overlaps the null, and includes important benefit (RR: <0.8) or harm (RR: >1.2) (0 P); ≥500 events and the 95% CI excludes the null values; ≥500 events but 95% CI overlaps the null, and excludes important benefit (RR: <0.8) or harm (RR: >1.2) (1 P).

^4^≤5 studies (0 P); 6-9 studies (if ≥10 studies; multiply by 2): *I*^2^ (H^2^ and/or tau^2^) (0.1 P), CIs for *I*^2^ (0.1 P), if *I*^2^ <40% (0.3 P), modelling detected heterogeneity (*I*^2^ ≥40%) with random effects model (0.1 P), exploring detected heterogeneity with subgroup analysis or meta-regression (0.1 P), sensitivity analyses with higher levels of heterogeneity (0.1 P).

^5^Differences in population; differences in intervention; surrogate markers; network meta-analysis (0 P); no important differences in population or intervention; hard clinical outcome (1 P).

^6^<5 studies OR evidence for severe bias with test or plot OR publication bias not assessed (0 P); no evidence for publication bias with test or plot (5-9 studies) OR evidence for moderate/small amount of publication bias with test or plot (0.5 P); no evidence for publication bias with test or plot (≥10 studies) (1 ).

^7^Industry funding OR conflict of interest (0 P); private institutions, foundations, non-governmental organizations (0.5 P); academic institutions, research institutions (1 P).

^8^Predicted RRs from restricted cubic spline. No effect (RR: 0.80-1.20) (0 P); moderate effect size (RR: <0.80-0.50 or >1.2-2.00) (1 P); large effect size (RR: <0.50 or >2.00) (2 P).

^9^No dose-response relationship (corresponding statistical test non- significant) (0 P); linear and/ or non-linear dose-response relationship (corresponding statistical test significant) (1 P)

**Supplemental Table 16** Quality of meta-analysis for ASBs: the NutriGrade scoring system^1^

| Items | The association between ASB intakes | | | | | |
| --- | --- | --- | --- | --- | --- | --- |
|  | T2D | Hypertension | CHD | Stroke | all-cause mortality | cardiovascular disease mortality |
| Risk of bias/ study quality/ study limitations^2^ | Average NOS score=7.20 (2 P) | Average NOS score=7.50 (2 P) | Average NOS score=7.67 (2 P) | Average NOS score=7.50 (2 P) | Average NOS score=7.50 (2 P) | Average NOS score=7.75 (2 P) |
| Precision^3^ | 21,290 events; RR: 1.32; 95% CI: 1.11, 1.56 (1 P) | 78,225 events; RR: 1.14; 95% CI: 1.10, 1.18 (1 P) | 6,943 events; RR: 1.11; 95% CI: 0.91, 1.35 (1 P) | 322 events; RR: 1.54; 95% CI: 1.05, 2.26 (0 P) | 95,704 events; RR: 1.12; 95% CI: 1.04, 1.21 (1 P) | 8,572 events; RR: 1.08; 95% CI: 1.00, 1.16 (1 P) |
| Heterogeneity^4^ | 10 cohorts; *I*^2^=92.7%, *P<*0.001; random-effects model with subgroup analysis (1 P) | 4 cohorts; *I*^2^=60.0%, *P*=0.06; random-effects model with subgroup analysis (0 P) | 3 cohorts; *I*^2^=65.6%, *P*=0.05; random-effects model without subgroup analysis (0 P) | 2 cohorts; *I*^2^=16.0%, *P*=0.28; random-effects model without subgroup analysis (0 P) | 7 cohorts; *I*^2^=81.9%, *P<*0.001; random-effects model with subgroup analysis (0.5 P) | 4 cohorts; *I*^2^=43.8%, *P*=0.15; random-effects model without subgroup analysis (0 P) |
| Directness^5^ | General populations; all studies investigated the association with dietary intakes of ASBs with T2D (1 P) | General populations; all studies investigated the association with dietary intakes of ASBs withhypertension (1 P) | General populations; all studies investigated the association with dietary intakes of ASBs with CHD (1 P) | General populations; all studies investigated the association with dietary intakes of ASBs with stroke (1 P) | General populations; all studies investigated the association with dietary intakes of ASBs with all-cause mortality (1 P) | General populations; all studies investigated the association with dietary intakes of ASBs with cardiovascular disease mortality (1 P) |
| Publication bias^6^ | 10 cohorts; no publication bias (1 P) | 4 cohorts; no publication bias (0 P) | 3 cohorts; no publication bias (0 P) | 2 cohorts; no publication bias (0 P) | 7 cohorts; no publication bias (0.5 P) | 4 cohorts; no publication bias (0 P) |
| Funding bias^7^ | Academic institutions (1 P) | Academic institutions (1 P) | Academic institutions (1 P) | Academic institutions (1 P) | Academic institutions (1 P) | Academic institutions (1 P) |
| Effect size^8^ | RR: 1.11; 95% CI: 1.05, 1.18 (0 P) | RR: 1.10; 95% CI: 1.08, 1.12 (0 P) | NC (0 P) | NC (0 P) | NC (0 P) | NC (0 P) |
| Dose-response^9^ | Linear dose-response relationship (1 P) | Linear dose-response relationship (1 P) | No dose-response relationship (0 P) | No dose-response relationship (0 P) | No dose-response relationship (0 P) | No dose-response relationship (0 P) |
| Overall score | 8.0 P | 6.0 P | 5.0 P | 4.0 P | 6.0 P | 5.0 P |

^1^ASB, artificially sweetened beverage; CHD, coronary heart disease; NOS, Newcastle-Ottawa Scale; NC, not calculatedP, points; T2D, type 2 diabetes.

^2^NOS score (mean): ≥7 (2 P); 4-6.9 (1 P); 0-3.9 (0 P).

^3^<500 events OR ≥500 events but 95% CI overlaps the null, and includes important benefit (RR: <0.8) or harm (RR: >1.2) (0 P); ≥500 events and the 95% CI excludes the null values; ≥500 events but 95% CI overlaps the null, and excludes important benefit (RR: <0.8) or harm (RR: >1.2) (1 P).

^4^≤5 studies (0 P); 6-9 studies (if ≥10 studies; multiply by 2): *I*^2^ (H^2^ and/or tau^2^) (0.1 P), CIs for *I*^2^ (0.1 P), if *I*^2^ <40% (0.3 P), modelling detected heterogeneity (*I*^2^ ≥40%) with random effects model (0.1 P), exploring detected heterogeneity with subgroup analysis or meta-regression (0.1 P), sensitivity analyses with higher levels of heterogeneity (0.1 P).

^5^Differences in population; differences in intervention; surrogate markers; network meta-analysis (0 P); no important differences in population or intervention; hard clinical outcome (1 P).

^6^<5 studies OR evidence for severe bias with test or plot OR publication bias not assessed (0 P); no evidence for publication bias with test or plot (5-9 studies) OR evidence for moderate/small amount of publication bias with test or plot (0.5 P); no evidence for publication bias with test or plot (≥10 studies) (1 ).

^7^Industry funding OR conflict of interest (0 P); private institutions, foundations, non-governmental organizations (0.5 P); academic institutions, research institutions (1 P).

^8^Predicted RRs from restricted cubic spline. No effect (RR: 0.80-1.20) (0 P); moderate effect size (RR: <0.80-0.50 or >1.2-2.00) (1 P); large effect size (RR: <0.50 or >2.00) (2 P).

^9^No dose-response relationship (corresponding statistical test non- significant) (0 P); linear and/ or non-linear dose-response relationship (corresponding statistical test significant) (1 P).

**Supplemental Table 17** Quality of meta-analysis for fruit juices: the NutriGrade scoring system^1^

| Items | The association between fruit juice intakes | | | | |
| --- | --- | --- | --- | --- | --- |
|  | T2D | Hypertension | Stroke | all-cause mortality | cardiovascular disease mortality |
| Risk of bias/ study quality/ study limitations^2^ | Average NOS score=6.50 (1 P) | Average NOS score=6.50 (1 P) | Average NOS score=6.50 (1 P) | Average NOS score=7.50 (2 P) | Average NOS score=7.50 (2 P) |
| Precision^3^ | 18,718 events; RR: 0.98; 95% CI: 0.93, 1.03 (0 P) | 46,817 events; RR: 1.01; 95% CI: 0.97, 1.04 (0 P) | 1,705 events; RR: 0.84; 95% CI: 0.51, 1.37 (0 P) | 5,904 events; RR: 1.26; 95% CI: 1.14, 1.40 (0 P) | 1,197 events; RR: 1.32; 95% CI: 1.03, 1.67 (0 P) |
| Heterogeneity^4^ | 6 cohorts; *I*^2^=0.0%, *P*=0.59; random-effects model with subgroup analysis (0.5 P) | 2 cohorts; *I*^2^=0.0%, *P*=0.69; random-effects model without subgroup analysis (0 P) | 2 cohorts; *I*^2^=86.1%, *P*=0.007; random-effects model without subgroup analysis (0 P) | 2 cohorts; *I*^2^=0.0%, *P*=0.65; random-effects model without subgroup analysis (0 P) | 2 cohorts; *I*^2^=0.0%, *P*=0.77; random-effects model without subgroup analysis (0 P) |
| Directness^5^ | General populations; all studies investigated the association with dietary intakes of fruit juices with T2D (1 P) | General populations; all studies investigated the association with dietary intakes of fruit juices with hypertension (1 P) | General populations; all studies investigated the association with dietary intakes of fruit juices with stroke (1 P) | General populations; all studies investigated the association with dietary intakes of fruit juices with all-cause mortality (1 P) | General populations; all studies investigated the association with dietary intakes of fruit juices with cardiovascular disease mortality (1 P) |
| Publication bias^6^ | 10 cohorts; no publication bias (1 P) | 2 cohorts; no publication bias (0 P) | 2 cohorts; no publication bias (0 P) | 2 cohorts; no publication bias (0 P) | 2 cohorts; no publication bias (0 P) |
| Funding bias^7^ | Academic institutions (1 P) | Academic institutions (1 P) | Academic institutions (1 P) | Academic institutions (1 P) | Academic institutions (1 P) |
| Effect size^8^ | NC (0 P) | NC (0 P) | NC (0 P) | NC (0 P) | NC (0 P) |
| Dose-response^9^ | Nonlinear dose-response relationship (1 P) | NO dose-response relationship (0 P) | NO dose-response relationship (0 P) | NO dose-response relationship (0 P) | NO dose-response relationship (0 P) |
| Overall score | 5.5 P | 3.0 P | 3.0 P | 4.0 P | 4.0 P |

^1^NOS, Newcastle-Ottawa Scale; NC, not calculated; P, points; T2D, type 2 diabetes.

^2^NOS score (mean): ≥7 (2 P); 4-6.9 (1 P); 0-3.9 (0 P).

^3^<500 events OR ≥500 events but 95% CI overlaps the null, and includes important benefit (RR: <0.8) or harm (RR: >1.2) (0 P); ≥500 events and the 95% CI excludes the null values; ≥500 events but 95% CI overlaps the null, and excludes important benefit (RR: <0.8) or harm (RR: >1.2) (1 P).

^4^≤5 studies (0 P); 6-9 studies (if ≥10 studies; multiply by 2): *I*^2^ (H^2^ and/or tau^2^) (0.1 P), CIs for *I*^2^ (0.1 P), if *I*^2^ <40% (0.3 P), modelling detected heterogeneity (*I*^2^ ≥40%) with random effects model (0.1 P), exploring detected heterogeneity with subgroup analysis or meta-regression (0.1 P), sensitivity analyses with higher levels of heterogeneity (0.1 P).

^5^Differences in population; differences in intervention; surrogate markers; network meta-analysis (0 P); no important differences in population or intervention; hard clinical outcome (1 P).

^6^<5 studies OR evidence for severe bias with test or plot OR publication bias not assessed (0 P); no evidence for publication bias with test or plot (5-9 studies) OR evidence for moderate/small amount of publication bias with test or plot (0.5 P); no evidence for publication bias with test or plot (≥10 studies) (1 ).

^7^Industry funding OR conflict of interest (0 P); private institutions, foundations, non-governmental organizations (0.5 P); academic institutions, research institutions (1 P).

^8^Predicted RRs from restricted cubic spline. No effect (RR: 0.80-1.20) (0 P); moderate effect size (RR: <0.80-0.50 or >1.2-2.00) (1 P); large effect size (RR: <0.50 or >2.00) (2 P).

^9^No dose-response relationship (corresponding statistical test non- significant) (0 P); linear and/ or non-linear dose-response relationship (corresponding statistical test significant) (1 P).

**Supplementary Table 18.** **Stratified analysis of the association between consumption of SSB, ASB, fruit juice and type 2 diabetes**

|  | **SSB** | | | | | **ASB** | | | | | **Fruit juice** | | | | |
| --- | --- | --- | --- | --- | --- | --- | --- | --- | --- | --- | --- | --- | --- | --- | --- |
|  | **n** | **RR (95% CI)** | ***I^2^*** | ***Ph*^*^** | ***Ph*^†^** | **n** | **RR (95% CI)** | ***I^2^*** | ***Ph*^*^** | ***Ph*^†^** | **n** | **RR (95% CI)** | ***I^2^*** | ***Ph*^*^** | ***Ph*^†^** |
| **All studies** | 17 | 1.27 (1.17, 1.38) | 69.2 | <0.001 |  | 10 | 1.32 (1.11, 1.56) | 92.7 | <0.001 |  | 6 | 0.98 (0.93, 1.03) | 0.0 | 0.59 |  |
| **Sex** |  |  |  |  | 0.38 |  |  |  |  | <0.001 |  |  |  |  | 0.61 |
| Both | 9 | 1.22 (1.09, 1.38) | 67.5 | 0.002 |  | 4 | 1.20 (1.00, 1.44) | 60.9 | 0.05 |  | 3 | 1.02 (0.89, 1.17) | 0.0 | 0.5 |  |
| Men | 2 | 1.21 (1.08, 1.36) | 0.0 | 0.74 |  | 2 | 1.93 (1.75, 2.13) | 0.0 | 0.58 |  | 0 | NA | NA | NA |  |
| Women | 6 | 1.33 (1.21, 1.47) | 33.6 | 0.18 |  | 4 | 1.15 (1.02, 1.29) | 67.8 | 0.03 |  | 3 | 0.98 (0.92, 1.04) | 3.4 | 0.36 |  |
| **Global region** |  |  |  |  | 0.36 |  |  |  |  | 0.47 |  |  |  |  | 0.40 |
| United States | 10 | 1.25 (1.12, 1.38) | 75.1 | <0.001 |  | 7 | 1.27 (1.05, 1.55) | 94.9 | <0.001 |  | 2 | 1.01 (0.89, 1.13) | 41.9 | 0.19 |  |
| Europe | 3 | 1.21 (1.05, 1.40) | 0.0 | 0.40 |  | 2 | 1.37 (0.96, 1.94) | 65.1 | 0.09 |  | 3 | 0.96 (0.86, 1.08) | 0.0 | 0.88 |  |
| Asia | 4 | 1.36 (1.22, 1.52) | 0.0 | 0.49 |  | 1 | 1.71 (1.11, 2.63) | NA | NA |  | 1 | 1.26 (0.86, 1.85) | NA | NA |  |
| **Follow-up** |  |  |  |  | 0.30 |  |  |  |  | 0.99 |  |  |  |  | 0.47 |
| <10y | 6 | 1.35 (1.17, 1.56) | 46.8 | 0.09 |  | 3 | 1.30 (1.11, 1.53) | 28.7 | 0.25 |  | 1 | 0.97 (0.91, 1.03) | NA | NA |  |
| ≥10y | 11 | 1.23 (1.13, 1.35) | 67.5 | 0.001 |  | 7 | 1.30 (1.05, 1.61) | 95.0 | <0.001 |  | 5 | 1.01 (0.92, 1.11) | 0.0 | 0.52 |  |
| **Assessment method** |  |  |  |  | 0.22 |  |  |  |  | 0.38 |  |  |  |  | 0.94 |
| FFQ | 16 | 1.28 (1.18, 1.40) | 71.1 | <0.001 |  | 9 | 1.34 (1.12, 1.60) | 93.5 | <0.001 |  | 5 | 0.98 (0.93, 1.04) | 0.0 | 0.44 |  |
| Diet record | 1 | 1.13 (0.94, 1.36) | NA | NA |  | 1 | 1.17 (0.93, 1.48) | NA | NA |  | 1 | 0.99 (0.80, 1.22) | NA | NA |  |
| **Body mass index** |  |  |  |  | 0.45 |  |  |  |  | <0.001 |  |  |  |  | 0.20 |
| Adjustment | 15 | 1.29 (1.17, 1.42) | 71.9 | <0.001 |  | 9 | 1.17 (1.07, 1.28) | 63.0 | 0.01 |  | 5 | 0.97 (0.92, 1.03) | 0.0 | 0.73 |  |
| Unadjustment | 2 | 1.22 (1.11, 1.34) | 0.0 | 0.81 |  | 1 | 1.94 (1.75, 2.15) | NA | NA |  | 1 | 1.11 (0.92, 1.34) | NA | NA |  |
| **Study quality score** |  |  |  |  | 0.95 |  |  |  |  |  |  |  |  |  | 0.41 |
| High | 13 | 1.27 (1.15, 1.40) | 73.0 | <0.001 |  | 7 | 1.35 (1.07, 1.70) | 94.7 | <0.001 | 0.51 | 3 | 1.03 (0.91, 1.17) | 39.5 | 0.19 |  |
| Medium | 4 | 1.26 (1.10, 1.46) | 40.6 | 0.17 |  | 3 | 1.22 (0.99, 1.49) | 65.2 | 0.06 |  | 3 | 0.96 (0.86, 1.08) | 0.0 | 0.88 |  |
| **Important confounders^#^** |  |  |  |  | 0.59 |  |  |  |  | 0.23 |  |  |  |  | 0.61 |
| Control | 7 | 1.24 (1.11, 1.37) | 21.5 | 0.27 |  | 4 | 1.17 (1.02, 1.34) | 59.5 | 0.06 |  | 3 | 0.98 (0.92, 1.04) | 3.4 | 0.36 |  |
| Not control | 10 | 1.29 (1.16, 1.44) | 78.3 | <0.001 |  | 6 | 1.40 (1.08, 1.81) | 95.6 | <0.001 |  | 3 | 1.02 (0.89, 1.17) | 0.0 | 0.50 |  |

^*^ P for heterogeneity within each subgroup;

^†^ P for heterogeneity between subgroups with a meta-regression analysis;

^#^ Important confounders included age, smoking, alcohol drinking, body mass index, physical activity, family history or past history;

ASB, artificially sweetened beverage; FFQ, food frequency questionnaire; NA, not applicable (because only 1 study); NC, not calculated; RR, relative risk; SSB, sugar-sweetened beverage; 95% CI, 95% confidence intervals.

**Supplementary Table 19.** **Stratified analysis of the association between consumption of SSBs, ASBs, and hypertension**

|  | **SSB** | | | | | **ASB** | | | | |
| --- | --- | --- | --- | --- | --- | --- | --- | --- | --- | --- |
|  | **n** | **RR (95% CI)** | ***I^2^*** | ***Ph*^*^** | ***Ph*^†^** | **n** | **RR (95% CI)** | ***I^2^*** | ***Ph*^*^** | ***Ph*^†^** |
| **All studies** | 8 | 1.12 (1.08, 1.17) | 50.3 | 0.05 |  | 4 | 1.14 (1.10, 1.18) | 60.0 | 0.06 |  |
| **Sex** |  |  |  |  | 0.16 |  |  |  |  | 0.03 |
| Both | 5 | 1.17 (1.06, 1.29) | 50.8 | 0.09 |  | 1 | 1.17 (0.95, 1.45) | NA | NA |  |
| Men | 1 | 1.06 (0.99, 1.14) | NA | NA |  | 1 | 1.20 (1.14, 1.26) | NA | NA |  |
| Women | 2 | 1.14 (1.09, 1.19) | 42.2 | 0.19 |  | 2 | 1.11 (1.09, 1.14) | 0.0 | 0.70 |  |
| **Global region** |  |  |  |  | 0.20 |  |  |  |  | NC |
| United States | 5 | 1.11 (1.06, 1.16) | 56.9 | 0.06 |  | 4 | 1.14 (1.10, 1.18) | 60.0 | 0.06 |  |
| Europe | 1 | 1.34 (1.09, 1.65) | NA | NA |  | 0 | - | - | - |  |
| Asia | 1 | 1.21 (1.02, 1.45) | NA | NA |  | 0 | - | - | - |  |
| Brazil | 1 | 1.23 (0.99, 1.53) | NA | NA |  | 0 | - | - | - |  |
| **Follow-up** |  |  |  |  | 0.04 |  |  |  |  | 0.80 |
| <10y | 4 | 1.24 (1.12, 1.37) | 0.0 | 0.84 |  | 1 | 1.17 (0.95, 1.45) | NA | NA |  |
| ≥10y | 4 | 1.10 (1.06, 1.16) | 66.4 | 0.03 |  | 3 | 1.14 (1.09, 1.18) | 72.9 | 0.03 |  |
| **Assessment method** |  |  |  |  | NC |  |  |  |  | NC |
| FFQ | 8 | 1.12 (1.08, 1.17) | 50.3 | 0.05 |  | 4 | 1.14 (1.10, 1.18) | 60.0 | 0.06 |  |
| Diet record | 0 | - | - | - |  | 0 | - | - | - |  |
| **Body mass index** |  |  |  |  | 0.86 |  |  |  |  | NC |
| Adjustment | 4 | 1.13 (1.07, 1.19) | 61.1 | 0.05 |  | 4 | 1.14 (1.10, 1.18) | 60.0 | 0.06 |  |
| Unadjustment | 4 | 1.12 (1.03, 1.22) | 30.6 | 0.23 |  | 0 | - | - | - |  |
| **Study quality score** |  |  |  |  | 0.61 |  |  |  |  | NC |
| High | 6 | 1.14 (1.09, 1.19) | 41.3 | 0.13 |  | 4 | 1.14 (1.10, 1.18) | 60.0 | 0.06 |  |
| Medium | 2 | 1.10 (0.97, 1.24) | 42.0 | 0.19 |  | 0 | - | - | - |  |
| **Important confounders^#^** |  |  |  |  | 0.08 |  |  |  |  | 0.007 |
| Control | 3 | 1.15 (1.09, 1.22) | 50.6 | 0.13 |  | 2 | 1.11 (1.09, 1.14) | 0.0 | 0.70 |  |
| Not control | 5 | 1.08 (1.03, 1.13) | 13.0 | 0.33 |  | 2 | 1.20 (1.14, 1.26) | 0.0 | 0.82 |  |

^*^ P for heterogeneity within each subgroup;

^†^ P for heterogeneity between subgroups with a meta-regression analysis;

^#^ Important confounders included age, smoking, alcohol drinking, body mass index, physical activity, family history or past history;

FFQ, food frequency questionnaire; NA, not applicable (because only 1 study); NC, not calculated;

RR, relative risk; SSB, sugar-sweetened beverage; 95% CI, 95% confidence intervals.

**Supplementary Table 20.** **Stratified analysis of** **the association between consumption of SSBs and cardiovascular disease**

|  | **Coronary heart disease** | | | | | **Stroke** | | | | |
| --- | --- | --- | --- | --- | --- | --- | --- | --- | --- | --- |
|  | **n** | **RR (95% CI)** | ***I^2^*** | ***Ph*^*^** | ***Ph*^†^** | **n** | **RR (95% CI)** | ***I^2^*** | ***Ph*^*^** | ***Ph*^†^** |
| **All studies** | 7 | 1.17 (1.09, 1.25) | 0.0 | 0.83 |  | 7 | 1.09 (1.00, 1.17) | 6.9 | 0.38 |  |
| **Sex** |  |  |  |  | 0.42 |  |  |  |  | 0.07 |
| Both | 4 | 1.10 (1.00, 1.24) | 0.0 | 0.94 |  | 4 | 1.00 (0.91, 1.11) | 0.0 | 0.77 |  |
| Men | 1 | 1.18 (1.06, 1.31) | NA | NA |  | 1 | 1.08 (0.82, 1.42) | NA | NA |  |
| Women | 2 | 1.26 (1.07, 1.47) | 0.0 | 0.40 |  | 2 | 1.20 (1.07, 1.35) | 0.0 | 0.89 |  |
| **Global region** |  |  |  |  | 0.55 |  |  |  |  | 0.09 |
| United States | 5 | 1.19 (1.10, 1.29) | 0.0 | 0.80 |  | 5 | 1.16 (1.05, 1.29) | 0.0 | 0.80 |  |
| Europe | 1 | 1.12 (0.97, 1.29) | NA | NA |  | 1 | 1.04 (0.92, 1.18) | NA | NA |  |
| Asia | 1 | 1.01 (0.71, 1.43) | NA | NA |  | 1 | 0.92 (0.75, 1.13) | NA | NA |  |
| **Follow-up** |  |  |  |  | 0.70 |  |  |  |  | 0.71 |
| <10y | 2 | 1.13 (0.93, 1.36) | 0.0 | 0.75 |  | 1 | 1.00 (0.65, 1.54) | NA | NA |  |
| ≥10y | 5 | 1.17 (1.09, 1.26) | 0.0 | 0.63 |  | 6 | 1.09 (1.00, 1.19) | 20.6 | 0.28 |  |
| **Assessment method** |  |  |  |  | NC |  |  |  |  | NC |
| FFQ | 7 | 1.17 (1.09, 1.25) | 0.0 | 0.83 |  | 7 | 1.17 (1.09, 1.25) | 0.0 | 0.83 |  |
| Diet record | 0 | - | - | - |  | 0 | - | - | - |  |
| **Body mass index** |  |  |  |  | 0.72 |  |  |  |  | 0.56 |
| Adjustment | 5 | 1.16 (1.07, 1.26) | 0.0 | 0.92 |  | 6 | 1.09 (1.00, 1.18) | 18.1 | 0.30 |  |
| Unadjustment | 2 | 1.20 (1.01, 1.43) | 46.7 | 0.17 |  | 1 | 0.88 (0.43, 1.79) | NA | NA |  |
| **Study quality score** |  |  |  |  | 0.82 |  |  |  |  | NC |
| High | 6 | 1.17 (1.09, 1.26) | 0.0 | 0.73 |  | 7 | 1.17 (1.09, 1.25) | 0.0 | 0.83 |  |
| Medium | 1 | 1.14 (0.93, 1.40) | NA | NA |  | 0 | - | - | - |  |
| **Important confounders^#^** |  |  |  |  | 0.72 |  |  |  |  | 0.83 |
| Control | 5 | 1.16 (1.07, 1.26) | 0.0 | 0.92 |  | 3 | 1.06 (0.86, 1.30) | 56.4 | 0.10 |  |
| Not control | 2 | 1.20 (1.01, 1.43) | 46.7 | 0.17 |  | 4 | 1.08 (0.99, 1.19) | 0.0 | 0.61 |  |

^*^ P for heterogeneity within each subgroup;

^†^ P for heterogeneity between subgroups with a meta-regression analysis;

^#^ Important confounders included age, smoking, alcohol drinking, body mass index, physical activity, family history or past history;

ASB, artificially sweetened beverage; FFQ, food frequency questionnaire; NA, not applicable (because only 1 study); NC, not calculated; RR, relative risk; SSB, sugar-sweetened beverage; 95% CI, 95% confidence intervals.

**Supplementary Table 21.** **Stratified analysis of the association between consumption of SSBs, ASBs and all-cause mortality**

|  | **SSB** | | | | | **ASB** | | | | |
| --- | --- | --- | --- | --- | --- | --- | --- | --- | --- | --- |
|  | **n** | **RR (95% CI)** | ***I^2^*** | ***Ph*^*^** | ***Ph*^†^** | **n** | **RR (95% CI)** | ***I^2^*** | ***Ph*^*^** | ***Ph*^†^** |
| **All studies** | 14 | 1.11 (1.05, 1.16) | 74.8 | <0.001 |  | 7 | 1.12 (1.04, 1.21) | 81.9 | <0.001 |  |
| **Sex** |  |  |  |  | 0.91 |  |  |  |  | 0.06 |
| Both | 10 | 1.09 (1.03, 1.16) | 72.2 | <0.001 |  | 4 | 1.15 (1.01, 1.31) | 87.6 | <0.001 |  |
| Men | 1 | 1.12 (1.00, 1.26) | NA | NA |  | 1 | 0.99 (0.91, 1.07) | NA | NA |  |
| Women | 3 | 1.12 (0.97, 1.29) | 77.9 | 0.01 |  | 2 | 1.17 (1.00, 1.37) | 65.9 | 0.09 |  |
| **Global region** |  |  |  |  | 0.01 |  |  |  |  | 0.01 |
| United States | 8 | 1.10 (1.04, 1.17) | 74.4 | <0.001 |  | 5 | 1.07 (1.00, 1.16) | 78.6 | 0.001 |  |
| Europe | 5 | 1.18 (1.05, 1.33) | 74.4 | 0.004 |  | 2 | 1.28 (1.14, 1.43) | 17.5 | 0.27 |  |
| Asia | 1 | 0.92 (0.81, 1.04) | NA | NA |  | 0 | - | - | - |  |
| **Follow-up** |  |  |  |  | 0.60 |  |  |  |  | 0.56 |
| <10y | 6 | 1.13 (1.02, 1.25) | 80.6 | <0.001 |  | 3 | 1.20 (0.93, 1.55) | 87.3 | <0.001 |  |
| ≥10y | 8 | 1.09 (1.03, 1.17) | 71.4 | 0.001 |  | 4 | 1.11 (1.03, 1.20) | 76.9 | 0.005 |  |
| **Assessment method** |  |  |  |  | 0.43 |  |  |  |  | 0.87 |
| FFQ | 12 | 1.09 (1.04, 1.15) | 64.4 | 0.001 |  | 5 | 1.13 (1.05, 1.22) | 74.9 | 0.003 |  |
| Diet record | 2 | 1.37 (0.79, 2.37) | 94.5 | <0.001 |  | 2 | 1.16 (0.80, 1.70) | 88.5 | 0.003 |  |
| **Body mass index** |  |  |  |  | NC |  |  |  |  | NC |
| Adjustment | 14 | 1.11 (1.05, 1.16) | 74.8 | <0.001 |  | 7 | 1.12 (1.04, 1.21) | 81.9 | <0.001 |  |
| Unadjustment | 0 | - | - | - |  | 0 | - | - | - |  |
| **Study quality score** |  |  |  |  | NC |  |  |  |  | NC |
| High | 14 | 1.11 (1.05, 1.16) | 74.8 | <0.001 |  | 7 | 1.12 (1.04, 1.21) | 81.9 | <0.001 |  |
| Medium | 0 | - | - | - |  | 0 | - | - | - |  |
| **Important confounders^#^** |  |  |  |  | 0.70 |  |  |  |  | <0.001 |
| Control | 6 | 1.12 (1.05, 1.20) | 79.4 | <0.001 |  | 4 | 1.05 (0.98, 1.13) | 78.2 | 0.003 |  |
| Not control | 8 | 1.09 (1.00, 1.20) | 74.1 | <0.001 |  | 3 | 1.27 (1.17, 1.38) | 0.0 | 0.53 |  |

^*^ P for heterogeneity within each subgroup;

^†^ P for heterogeneity between subgroups with a meta-regression analysis;

^#^ Important confounders included age, smoking, alcohol drinking, body mass index, physical activity, family history or past history;

ASB, artificially sweetened beverage; FFQ, food frequency questionnaire; NA, not applicable (because only 1 study); NC, not calculated; RR, relative risk; SSB, sugar-sweetened beverage; 95% CI, 95% confidence intervals.

**Supplementary Table 22.** **Stratified analysis of** **the association between consumption of SSBs, ASBs and cardiovascular disease mortality**

|  | **SSB** | | | | | **ASB** | | | | |
| --- | --- | --- | --- | --- | --- | --- | --- | --- | --- | --- |
|  | **n** | **RR (95% CI)** | ***I^2^*** | ***Ph*^*^** | ***Ph*^†^** | **n** | **RR (95% CI)** | ***I^2^*** | ***Ph*^*^** | ***Ph*^†^** |
| **All studies** | 9 | 1.13 (1.05, 1.20) | 14.0 | 0.32 |  | 4 | 1.08(1.00, 1.16) | 43.8 | 0.15 |  |
| **Sex** |  |  |  |  | 0.85 |  |  |  |  | 0.26 |
| Both | 5 | 1.10 (1.03, 1.18) | 0.0 | 0.94 |  | 1 | 0.99 (0.87, 1.12) | NA | NA |  |
| Men | 1 | 1.19 (0.95, 1.49) | NA | NA |  | 1 | 1.06 (0.91, 1.24) | NA | NA |  |
| Women | 3 | 1.13 (0.88, 1.45) | 70.1 | 0.04 |  | 2 | 1.22 (0.98, 1.53) | 34.3 | 0.22 |  |
| **Global region** |  |  |  |  | 0.42 |  |  |  |  | NC |
| United States | 8 | 1.13 (1.06, 1.22) | 19.3 | 0.28 |  | 4 | 1.08(1.00, 1.16) | 43.8 | 0.15 |  |
| Europe | 0 | - | - | - |  | 0 | - | - | - |  |
| Asia | 1 | 1.03 (0.82, 1.29) | NA | NA |  | 0 | - | - | - |  |
| **Follow-up** |  |  |  |  | 0.35 |  |  |  |  | 0.83 |
| <10y | 5 | 1.10 (1.02, 1.17) | 0.0 | 0.66 |  | 2 | 1.16 (0.78, 1.73) | 73.3 | 0.05 |  |
| ≥10y | 4 | 1.18 (1.03, 1.34) | 41.5 | 0.16 |  | 2 | 1.11 (1.01, 1.23) | 0.0 | 0.43 |  |
| **Assessment method** |  |  |  |  | 0.77 |  |  |  |  | 0.12 |
| FFQ | 8 | 1.13 (1.03, 1.24) | 23.1 | 0.25 |  | 3 | 1.14 (1.01, 1.28) | 23.8 | 0.27 |  |
| Diet record | 1 | 1.11 (1.02, 1.20) | NA | NA |  | 1 | 0.99 (0.87, 1.12) | NA | NA |  |
| **Body mass index** |  |  |  |  | 0.45 |  |  |  |  | NC |
| Adjustment | 8 | 1.14 (1.05, 1.22) | 20.0 | 0.27 |  | 4 | 1.08(1.00, 1.16) | 43.8 | 0.15 |  |
| Unadjustment | 1 | 1.05 (0.87, 1.27) | NA | NA |  | 0 | - | - | - |  |
| **Study quality score** |  |  |  |  | NC |  |  |  |  | NC |
| High | 9 | 1.13 (1.05, 1.20) | 14.0 | 0.32 |  | 4 | 1.08(1.00, 1.16) | 43.8 | 0.15 |  |
| Medium | 0 | - | - | - |  | 0 | - | - | - |  |
| **Important confounders^#^** |  |  |  |  | 0.50 |  |  |  |  | NC |
| Control | 7 | 1.14 (1.04, 1.24) | 30.6 | 0.20 |  | 4 | 1.08(1.00, 1.16) | 43.8 | 0.15 |  |
| Not control | 2 | 1.07 (0.92, 1.25) | 0.0 | 0.64 |  | 0 | - | - | - |  |

^*^ P for heterogeneity within each subgroup;

^†^ P for heterogeneity between subgroups with a meta-regression analysis;

^#^ Important confounders included age, smoking, alcohol drinking, body mass index, physical activity, family history or past history;

ASB, artificially sweetened beverage; FFQ, food frequency questionnaire; NA, not applicable (because only 1 study); NC, not calculated; RR, relative risk; SSB, sugar-sweetened beverage; 95% CI, 95% confidence intervals.

**Supplementary figures**


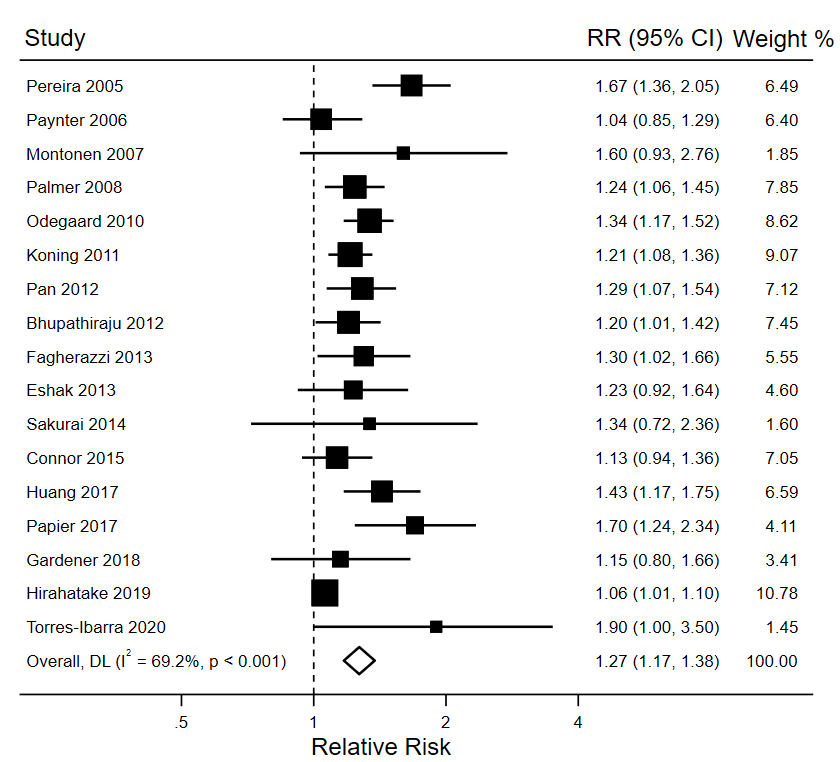


**Supplementary Figure 1.** Forest plot of type 2 diabetes for the highest versus lowest categories of sugar-sweetened beverages consumption

RR，relative risk; 95% CI, 95% confidence intervals.


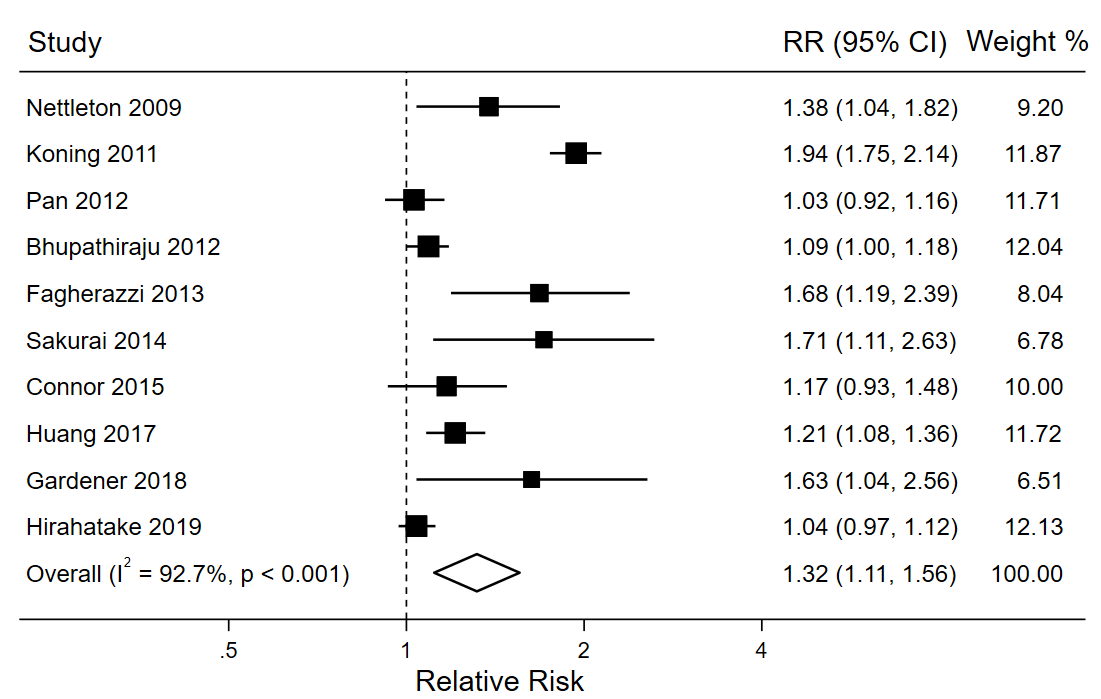


**Supplementary Figure 2.** Forest plot of type 2 diabetes for the highest versus lowest categories of artificially sweetened beverages consumption

RR，relative risk; 95% CI, 95% confidence intervals.


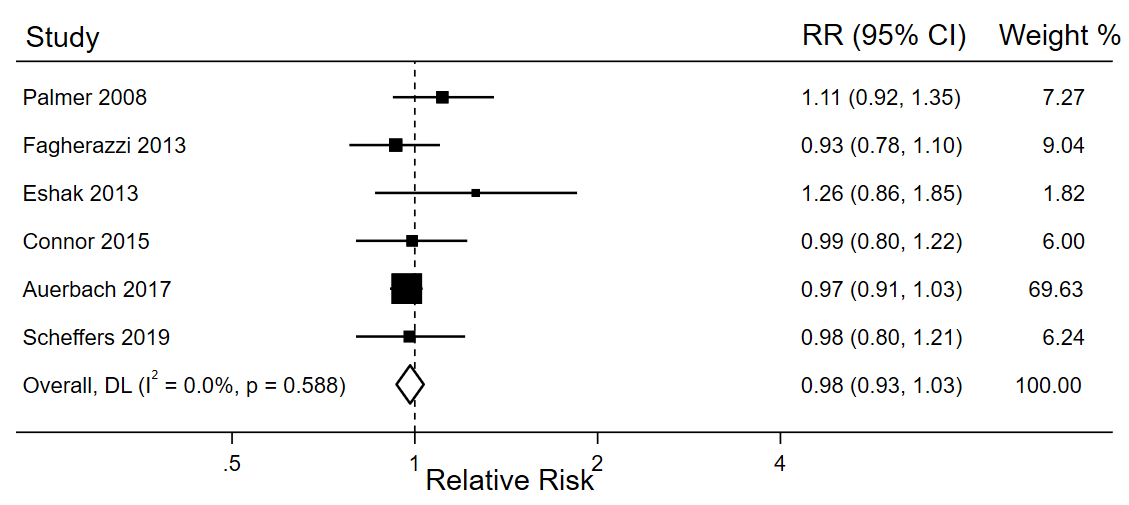


**Supplementary Figure 3.** Forest plot of type 2 diabetes for the highest versus lowest categories of fruit juice consumption

RR，relative risk; 95% CI, 95% confidence intervals.


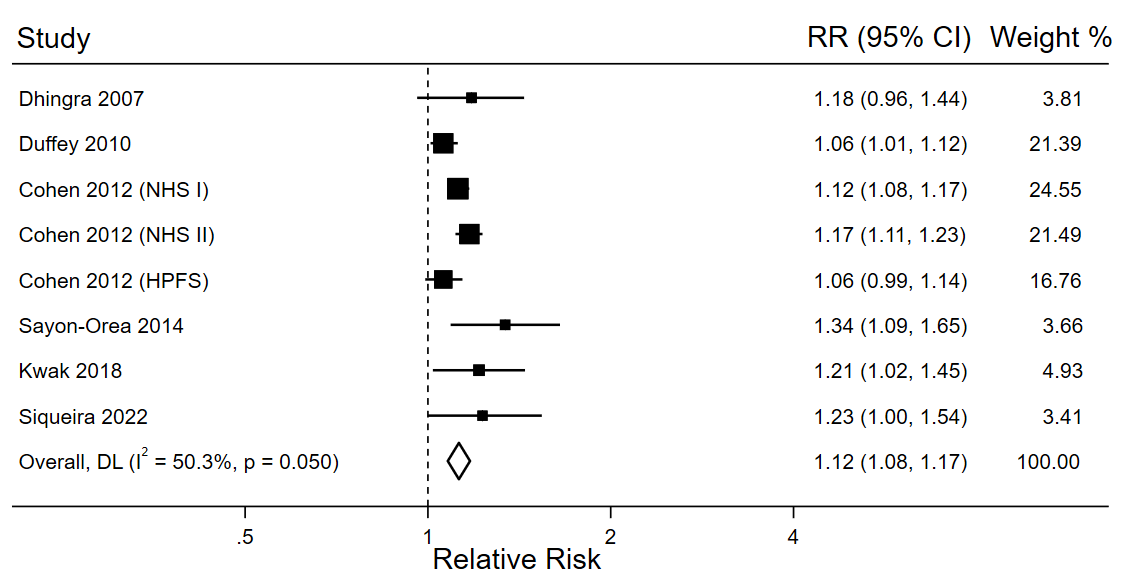


**Supplementary Figure 4.** Forest plot of hypertension for the highest versus lowest categories of sugar-sweetened beverages consumption

HPFS, Health Professionals Follow-Up Study; NHS I, Nurses’ Health Study; NHS II, Nurses’ Health Study II; RR，relative risk; 95% CI, 95% confidence intervals.


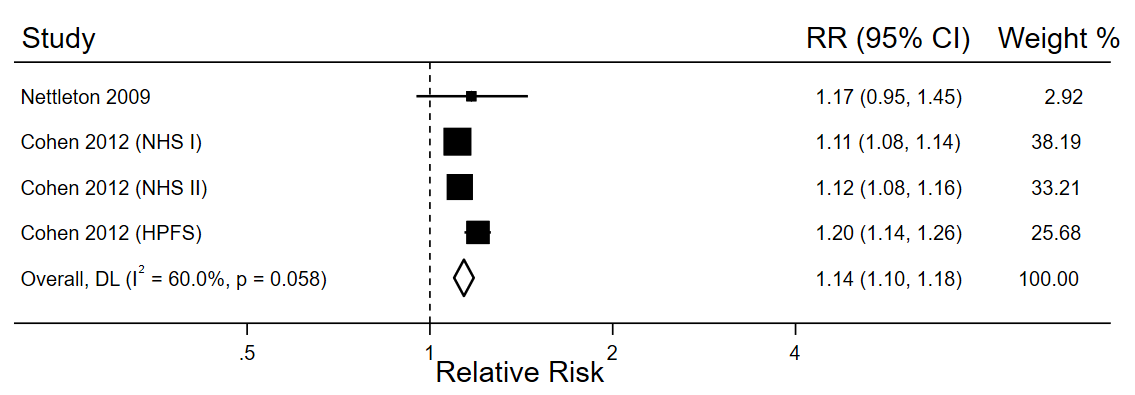


**Supplementary Figure 5.** Forest plot of hypertension for the highest versus lowest categories of artificially sweetened beverages consumption

HPFS, Health Professionals Follow-Up Study; NHS I, Nurses’ Health Study; NHS II, Nurses’ Health Study II; RR，relative risk; 95% CI, 95% confidence intervals.


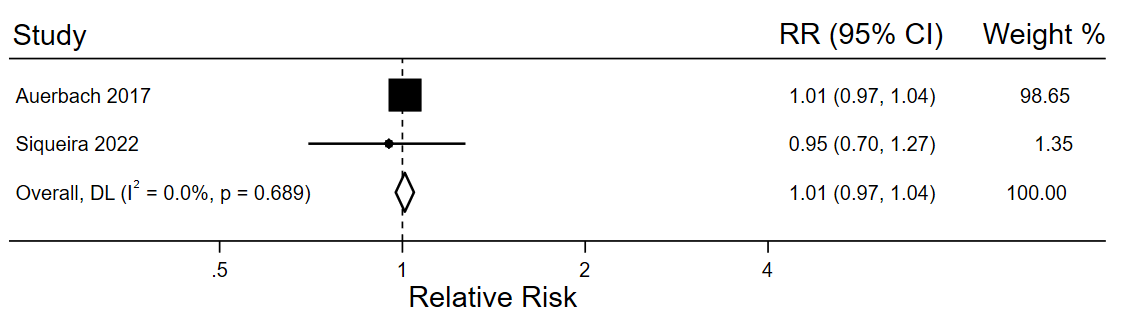


**Supplementary Figure 6.** Forest plot of hypertension for the highest versus lowest categories of fruit juice consumption

RR，relative risk; 95% CI, 95% confidence intervals.


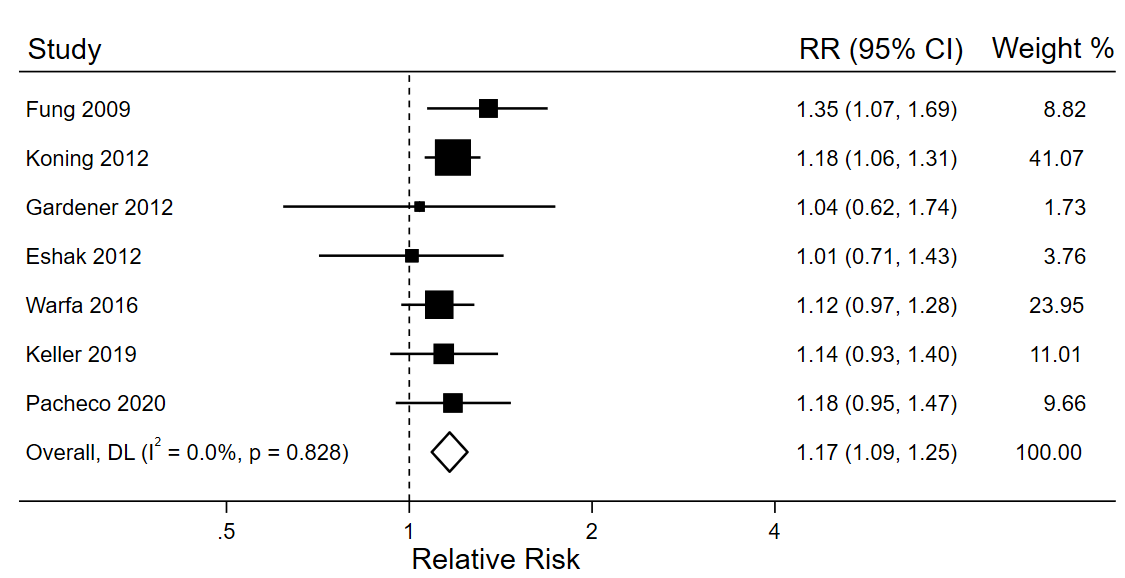


**Supplementary Figure 7.** Forest plot of coronary heart disease for the highest versus lowest categories of sugar-sweetened beverages consumption

RR，relative risk; 95% CI, 95% confidence intervals.


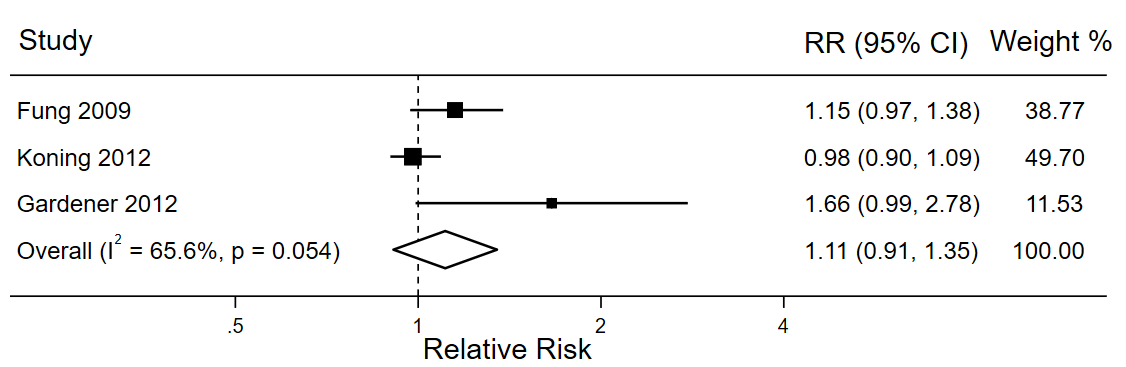


**Supplementary Figure 8.** Forest plot of coronary heart disease for the highest versus lowest categories of artificially sweetened beverages consumption

RR，relative risk; 95% CI, 95% confidence intervals.


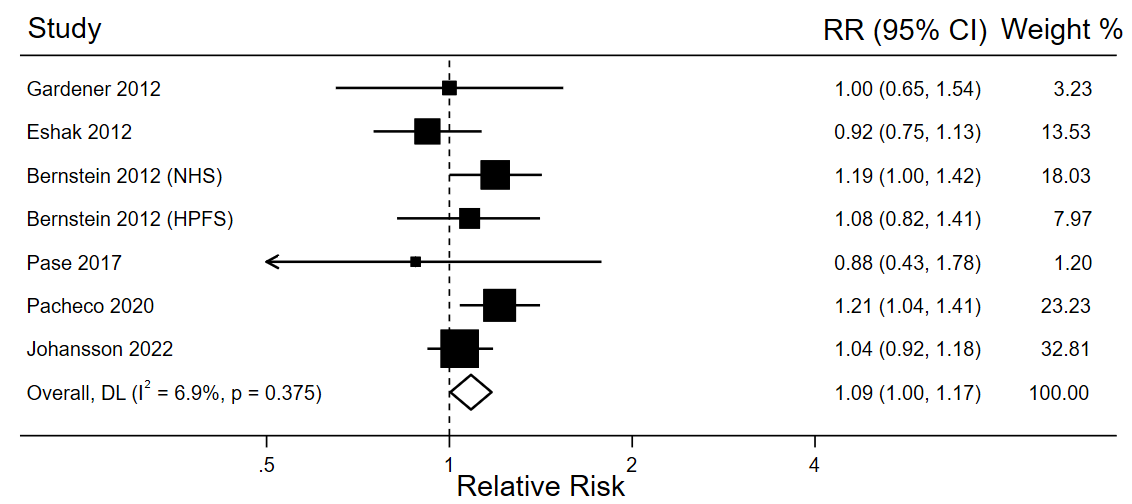


**Supplementary Figure 9.** Forest plot of stroke for the highest versus lowest categories of sugar-sweetened beverages consumption

CSM, Cohort of Swedish Men; HPFS, Health Professionals Follow-Up Study; NHS, Nurses’ Health Study; RR，relative risk; SMC, Swedish Mammography Cohort; 95% CI, 95% confidence intervals.


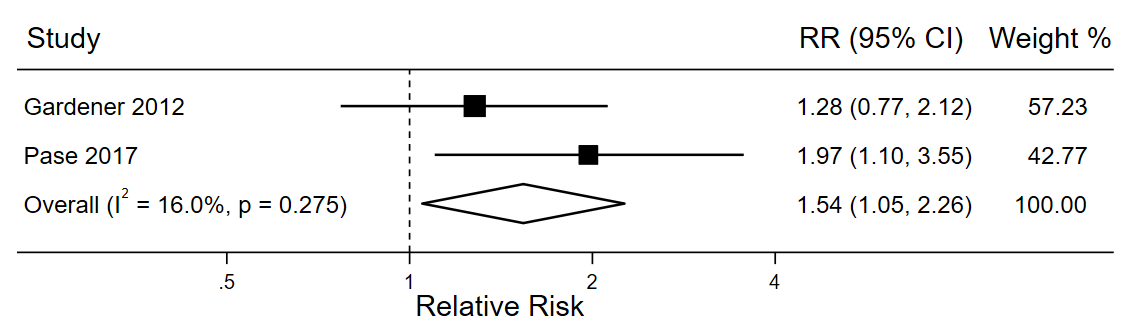


**Supplementary Figure 10.** Forest plot of stroke for the highest versus lowest categories of artificially sweetened beverages consumption

RR，relative risk; 95% CI, 95% confidence intervals.


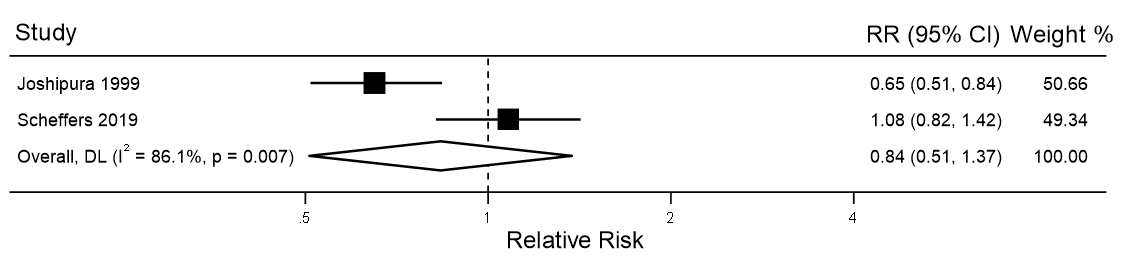


**Supplementary Figure 11.** Forest plot of stroke for the highest versus lowest categories of fruit juice consumption

RR，relative risk; 95% CI, 95% confidence intervals.


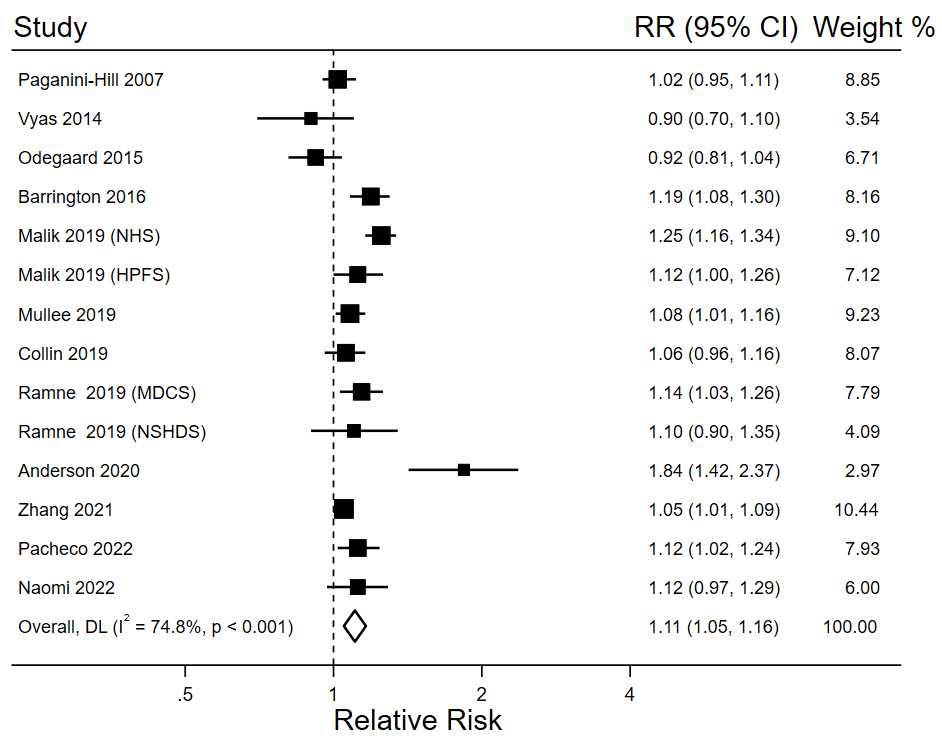


**Supplementary Figure 12.** Forest plot of all-cause mortality for the highest versus lowest categories of sugar-sweetened beverages consumption

HPFS, Health Professionals Follow-Up Study; NHS, Nurses’ Health Study; RR，relative risk; 95% CI, 95% confidence intervals.


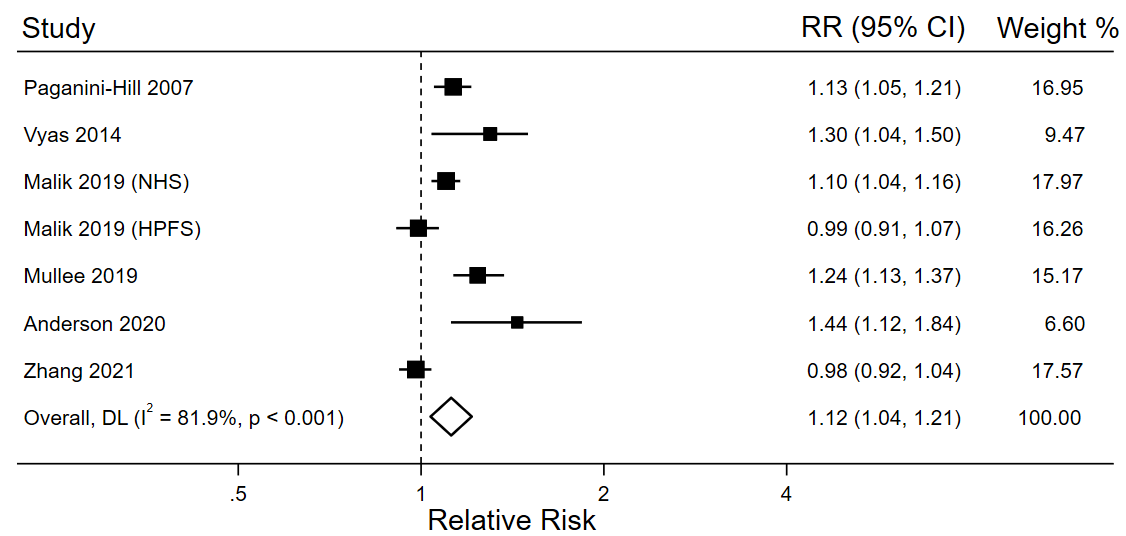


**Supplementary Figure 13.** Forest plot of all-cause mortality for the highest versus lowest categories of artificially sweetened beverages consumption

HPFS, Health Professionals Follow-Up Study; NHS, Nurses’ Health Study; RR，relative risk; 95% CI, 95% confidence intervals.


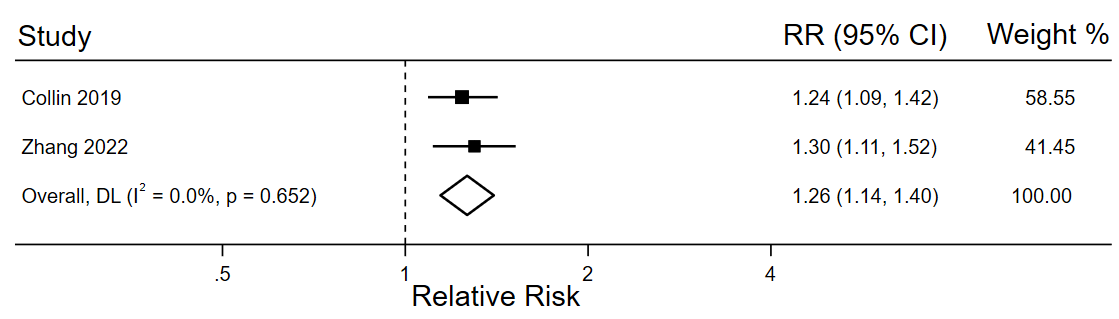


**Supplementary Figure 14.** Forest plot of all-cause mortality for the highest versus lowest categories of fruit juice consumption

RR，relative risk; 95% CI, 95% confidence intervals.


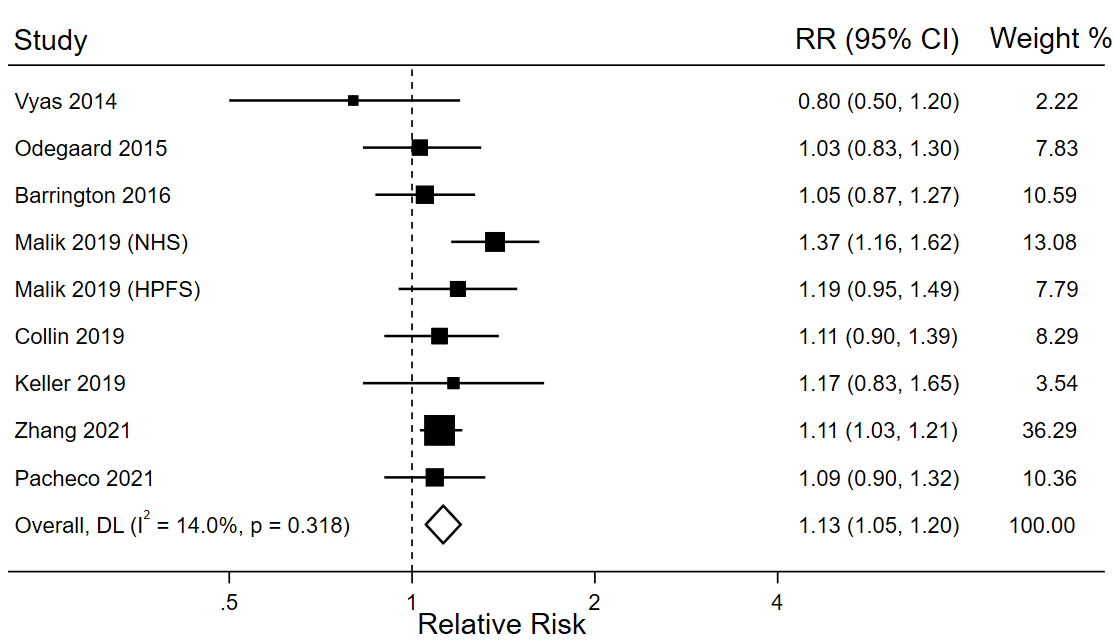


**Supplementary Figure 15.** Forest plot of cardiovascular disease mortality for the highest versus lowest categories of sugar-sweetened beverages consumption

HPFS, Health Professionals Follow-Up Study; NHS, Nurses’ Health Study; RR，relative risk; 95% CI, 95% confidence intervals.


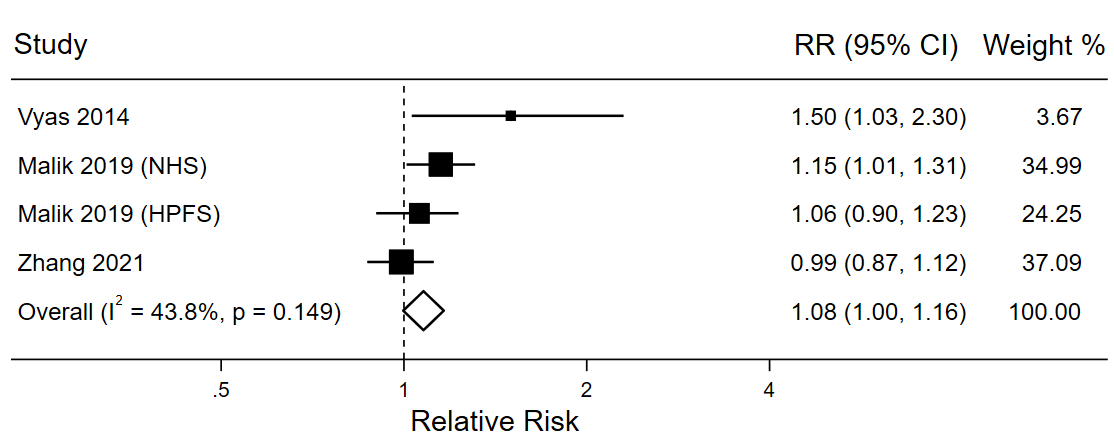


**Supplementary Figure 16.** Forest plot of cardiovascular disease mortality for the highest versus lowest categories of artificially sweetened beverages consumption

HPFS, Health Professionals Follow-Up Study; NHS, Nurses’ Health Study; RR，relative risk; 95% CI, 95% confidence intervals.


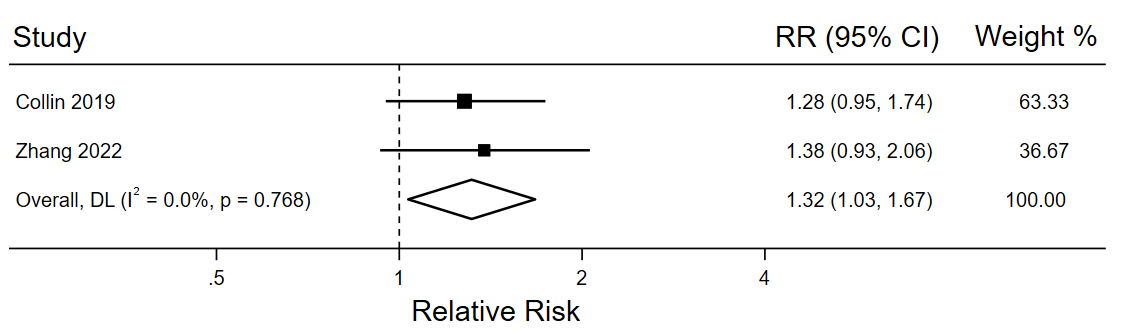


**Supplementary Figure 17.** Forest plot of cardiovascular disease mortality for the highest versus lowest categories of fruit juice consumption

RR，relative risk; 95% CI, 95% confidence intervals.


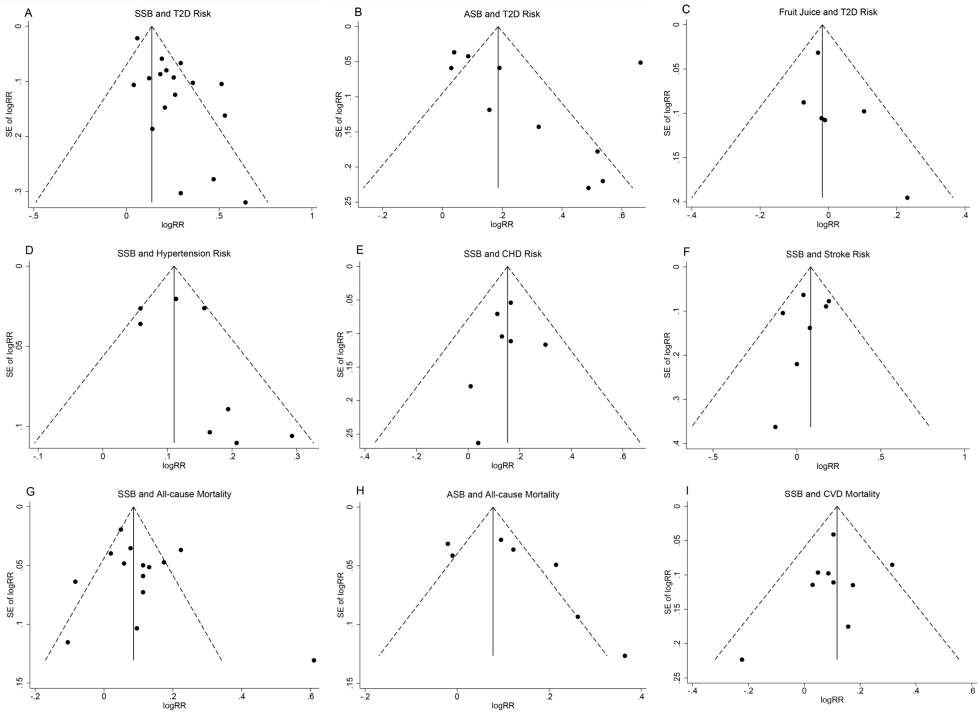


**Supplementary Figure 18.** Funnel plots with SSB, ASB or fruit juice intake and risk of T2D (A, B, and C), hypertension (D), CHD (E), stroke (F), all-cause mortality (G and H) and CVD mortality (I).

ASB, artificially sweetened beverage; CHD, coronary heart disease; CVD, cardiovascular disease; RR, relative risk; SE, standard error; SSB, sugar-sweetened beverage; T2D, type 2 diabetes.


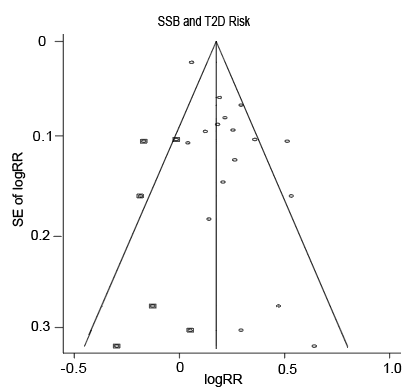


**Supplementary Figure 19.** Trim and fill funnel plot showing correction of publication bias for SSB intake and risk of T2D after addition of seven missing hypothetical studies.

RR, relative risk; SE, standard error; SSB, sugar-sweetened beverage; T2D, type 2 diabetes.
